# Molecular Systematics and Distribution Pattern Evolution of *Triplophysa* Species in China: A Comparison of Widespread and Narrow-Range Taxa

**DOI:** 10.3390/biology15181667

**Published:** 2026-09-20

**Authors:** Yixuan Chen, Minyi Wang, Ziyi Wang, Xiao Chen, Shengnan Li, Conghui Yang, Dengqiang Wang, Xinbin Duan, Huiwu Tian, Qianhong Gu

**Affiliations:** 1 Engineering Research Center of Polyploid Fish Reproduction and Breeding of the State Education Ministry, College of Life Sciences, Hunan Normal University, Changsha 410081, China; 202230233009@hunnu.edu.cn (Y.C.); 202230233004@hunnu.edu.cn (M.W.); 202130233024@hunnu.edu.cn (Z.W.); chenxiao01s@163.com (X.C.); shnli@hunnu.edu.cn (S.L.); yangch@hunnu.edu.cn (C.Y.); 2 Fishes Conservation and Utilization in the Upper Reaches of the Yangtze River Key Laboratory of Sichuan Province, Yangtze River Fisheries Research Institute, Chinese Academy of Fishery Sciences, Neijiang 641100, China; wdq@yfi.ac.cn (D.W.); duan@yfi.ac.cn (X.D.); 3 Yangtze River Fisheries Research Institute, Chinese Academy of Fishery Sciences, Wuhan 430223, China

**Keywords:** *Triplophysa*, phylogenetics, species distribution model, Qinghai–Tibet Plateau, conservation genetics

## Abstract

The genus *Triplophysa* (Nemacheilidae), a dominant component of the Qinghai–Tibet Plateau (QTP) fish fauna, remains poorly understood in its evolutionary relationships and phylogeography. We integrated mitochondrial phylogenomics and population genetics (*COI* and *cytb*) with MaxEnt modeling across four time periods—the present, the Last Interglacial (LIG), the Last Glacial Maximum (LGM), and the Mid-Holocene Optimum (MHO)—to examine 12 *Triplophysa* species, ranging from widely distributed to narrowly endemic taxa. Phylogenetic analyses recovered a monophyletic *Triplophysa* with a clear split between cave- and non-cave-dwelling lineages. Six range-restricted species (*T. stenura*, *T. dalaica*, *T. siluroides*, *T. obscura*, *T. hsutschouensis*, and *T. minxianensis*) formed reciprocally monophyletic clades, whereas widespread species (*T. leptosoma*, *T. stoliczkae*, *T. brevicauda*, *T. orientalis*, and *T. bleekeri*) exhibited non-monophyletic patterns in mitochondrial gene trees, likely reflecting incomplete lineage sorting or mitochondrial capture, though distinguishing these processes requires nuclear data. A positive association between genetic diversity and range size was observed for *COI* nucleotide diversity when two bottleneck-affected species were excluded. Paleoclimatic reconstructions revealed that suitable habitats expanded to their maximum during the LGM and contracted during interglacials, highlighting two climatically stable refugia in the central Loess Plateau–northern Qinling foothills and the eastern QTP margin–northern Hengduan Mountains. Our results demonstrate a tight coupling between phylogenetic patterns and range dynamics, with narrowly distributed endemics exhibiting both monophyly and reduced genetic diversity. These findings identify genetically vulnerable species as conservation priorities and provide a scientific framework for safeguarding *Triplophysa* diversity in a warming world.

## Simple Summary

*Triplophysa* loaches are a diverse fish group that dominates the rivers and lakes of the Qinghai–Tibet Plateau, yet their evolutionary history and distribution dynamics remain poorly understood. We studied 12 species with contrasting range sizes—from widespread plateau inhabitants to narrow-range endemics—using genetic data and habitat models across four time periods. Narrowly distributed species formed distinct genetic groups and had low genetic diversity, making them more vulnerable to environmental change, whereas widespread species showed complex genetic patterns. Habitat models showed that suitable habitats expanded during the last ice age and shrank as the climate warmed, leaving only a few stable refuges in the central Loess Plateau and along the eastern plateau edge. These refuges likely served as long-term shelters through past climate fluctuations. Our findings identify narrow-range endemics with low genetic diversity as priority conservation targets and highlight key areas that deserve protection to safeguard this unique fish group under ongoing climate warming.

## 1. Introduction

The genus *Triplophysa* (Cypriniformes: Nemacheilidae) is the most species-rich nemacheilid group and one of the three principal fish components of the Qinghai–Tibet Plateau (QTP) [1,2,3]. As some of the highest-dwelling freshwater fishes on Earth, *Triplophysa* species, together with schizothoracin fishes, dominate the QTP fish fauna and play a central role in its river and lake ecosystems [1].

*Triplophysa* ranges widely across the Central Asian highlands. In China, it is concentrated in the upper reaches of the Yellow River, Yangtze River, Lancang River, Nu River, and Yarlung Zangbo River, as well as in endorheic basins including the Qaidam Basin and the Tarim River [2,3]. Beyond the main plateau, some species inhabit southwestern karst caves and show troglomorphic traits (e.g., eye regression, depigmentation); 43 cave-dwelling species have been described, mostly from the Xijiang River system of the Pearl River basin [4,5,6]. The genus originated at the Oligocene–Miocene boundary, and its radiation proceeded in concert with QTP uplift [7,8]. This diversification created striking disparities in species range sizes. For instance, *T. stoliczkae* is exceptionally widespread. It reaches a record altitude of 5200 m at Longcuo Hot Spring in Tibet (http://english.ihb.cas.cn/research/achievements/202011/t20201126_253626.html, accessed on 24 June 2026). By contrast, most congeneric species are narrowly distributed. They are restricted to single drainages or even specific caves [4,6]. They exhibit pronounced local endemism. These distributional contrasts make *Triplophysa* an ideal system for studying speciation, dispersal, and isolation.

The phylogeny of *Triplophysa* remains contentious, and morphology-based taxonomy has long been confounded by character plasticity [9]. He et al. (2006) recovered the genus as non-monophyletic using the *cytb* gene, whereas mitogenomic analyses support its monophyly [3,7]. Recent studies consistently resolve separate epigean and cave clades, a pattern suggesting a single origin of the cave phenotype [10,11]. However, persistent mito-nuclear discordance and widespread interspecific introgression create strong phylogenetic conflict. Genome-wide data confirm introgression as the primary driver of this conflict, with effects far outweighing those of incomplete lineage sorting (ILS) [12,13]. Consequently, several fundamental systematic issues remain unresolved: the monophyly of the genus, relationships among major clades, and the phylogenetic placement of widespread versus narrow-range species.

With over 100 species recognized worldwide and 164 valid species currently listed in FishBase (accessed 24 June 2026), *Triplophysa* is distributed predominantly in China. Over 70 new species have been described in the past two decades, a taxonomic proliferation driven largely by morphological conservatism, simple body architecture, and drainage fragmentation that restricts dispersal and gene flow, all of which promote extensive cryptic diversity [14,15]. Despite its rich and growing diversity, the genus faces acute conservation threats: multiple species are nationally protected in China, roughly one-fifth of assessed taxa are threatened in the national red list, and several are listed as Endangered or Critically Endangered by the IUCN, with some once feared extinct [16]. Slow growth and low reproductive potential prevail across *Triplophysa* species, rendering population recovery extremely difficult once populations become depleted [17,18].

These conservation threats are further amplified by global warming. As high-elevation cold-water specialists, *Triplophysa* species are highly sensitive to climate change [8,19,20,21,22,23]. Ongoing warming on the QTP has driven accelerated glacier melt, permafrost degradation, and profound shifts in lake physicochemistry. Between 1986 and 2022, lakes > 1 km^2^ across the plateau expanded markedly in area; mean surface water temperature rose by 1.33 °C, water transparency increased, and mean salinity fell by nearly half, directly degrading habitats, foraging grounds and spawning sites [24]. Population genomic analyses of sympatric species reveal distinct salinity-adaptation mechanisms across taxa [25], indicating species-specific responses to environmental change. Species distribution models show suitable habitat for widespread species peaked during the Last Glacial Maximum and contracted with postglacial warming [23]. This counter-intuitive pattern reflects the cold-adapted nature of *Triplophysa*: colder glacial climates expanded the extent of high-altitude cold-water habitats suitable for these fishes, whereas postglacial warming reduced such habitats, particularly in lower-elevation areas. The demographic histories of multiple species are tightly linked to QTP uplift and Quaternary glacial–interglacial cycles [13,26,27]. Projected future warming will likely further shrink their suitable habitat [28]. These climate impacts are compounded by anthropogenic stressors including dam construction, water pollution, overfishing and exotic fish introductions, jointly accelerating population declines. Developing science-based conservation strategies for *Triplophysa* under climate change is thus an urgent priority.

To tackle these pressing conservation challenges, species distribution models (SDMs), especially MaxEnt, function as potent analytical tools that generate reliable projections of climate-induced range shifts, even when working with limited occurrence datasets [29]. Molecular phylogenetics and phylogeography complement these spatial analyses by revealing evolutionary histories, genetic diversity patterns and population dynamics, insights that directly inform conservation prioritization [30,31,32]. For *Triplophysa*, Wu et al. (2020) linked its Pliocene diversification to rapid uplift of the Qinling and Taihang Mountains, and attributed reshaped genetic structure to Early Pleistocene dispersal events in the middle Yellow River [8]. Convergent phylogeographic evidence further confirms that QTP orogenesis and Quaternary glacial–interglacial cycles jointly drove lineage divergence and population demography across the genus [15,23,27], with widespread interspecific introgression also fueling its reticulate adaptive radiation alongside plateau uplift [13]. In conservation genetics, a robust body of empirical work demonstrates a significant positive correlation between genetic diversity and both population fitness and long-term viability [33,34], underscoring the conservation value of integrating genetic evidence with spatial distribution projections. Nevertheless, current *Triplophysa* research remains heavily skewed toward taxonomic species description, and systematic investigations that couple distributional modeling with evolutionary phylogenetic perspectives remain scarce.

To address this research gap, the present study focuses on 12 *Triplophysa* species across China and pursues three core objectives. First, we reconstruct the phylogenetic relationships of these species using mitochondrial genomic data to resolve their evolutionary affinities. We explicitly acknowledge that these mitochondrial phylogenies represent gene trees rather than species trees, and that resolving the true species relationships will require nuclear data. Second, we integrate population genetic analyses—including genetic diversity, population structure, haplotype networks, and demographic history—to characterize the genetic architecture and historical dynamics of these species. Third, we implement MaxEnt ecological niche modeling to project potential distributions under contemporary and three paleoclimatic periods (LIG, LGM, and MHO), revealing spatiotemporal shifts in suitable habitat. By integrating molecular phylogenetic, population genetic, and distributional evidence, we aim to examine the coupling between evolutionary history, genetic diversity patterns, and geographic range dynamics, identify climatically stable refugia and genetically vulnerable populations, and provide a scientific foundation for safeguarding *Triplophysa* species diversity in a warming world.

## 2. Materials and Methods

### 2.1. Samples and Sequence Data Preparation

To further test the monophyly of *Triplophysa* in molecular phylogenetic trees, we downloaded all currently available mitogenome data of *Triplophysa* fishes from GenBank for phylogenetic reconstruction. To perform a comprehensive phylogenetic analysis, we also retrieved mitogenome sequences from Nemacheilidae (*n* = 159), Botiidae (*n* = 18), Ellopostomatidae (*n* = 1), Catostomidae (*n* = 22), Cobitidae (*n* = 36), Gonorynchidae (*n* = 1), Gyrinocheilidae (*n* = 3), Balitoridae (*n* = 13), Alestidae (*n* = 1), and Vaillantellidae (*n* = 1), yielding a total of 255 sequences. Among these, 93 sequences belong to *Triplophysa* species, including some duplicate conspecific samples (Table S1) that allow verification of the reliability of species identifications. To supplement the representation of cave-dwelling *Triplophysa*, one new mitogenome of *Triplophysa erythraea* was sequenced using standard PCR-based Sanger sequencing with 15 overlapping primer pairs designed from published *Triplophysa* mitogenomes (the methods for DNA extraction, amplification, and sequencing are provided in Additional File S1 in the Supplementary Materials).

In addition, we downloaded 746 *COI* and 877 *cytb* gene sequences of 12 *Triplophysa* species from GenBank (Table S2). These sequences were used for molecular phylogenetic and genetic diversity analyses of the 12 *Triplophysa* species. For these 12 species, we compiled population genetic studies and associated sample distribution records, and gathered occurrence data (Table S3) for each species from the Global Biodiversity Information Facility (GBIF.org, https://www.gbif.org (accessed on 28 March 2026) GBIF Occurrence Download, https://doi.org/10.15468/dl.xdzbmd; GBIF.org (accessed on 25 March 2026) GBIF Occurrence Download https://doi.org/10.15468/dl.tfkcjg; GBIF.org (accessed on 24 March 2026) GBIF Occurrence Download https://doi.org/10.15468/dl.y8cjun; GBIF.org (accessed on 24 March 2026) GBIF Occurrence Download https://doi.org/10.15468/dl.s2dzt5; GBIF.org (accessed on 24 March 2026) GBIF Occurrence Download https://doi.org/10.15468/dl.wqqehd; GBIF.org (accessed on 24 March 2026) GBIF Occurrence Download https://doi.org/10.15468/dl.en6k2f) and published literature, to be used in species distribution modeling. It should be emphasized that the 12 focal species analyzed in the population genetic and distribution modeling components are all epigean (non-cave-dwelling) taxa. Cave-dwelling *Triplophysa* species were included only in the broader mitogenomic phylogenetic analysis (Section 3.1) to assess genus monophyly and the phylogenetic position of cave lineages.

It should be noted that this study adopts an integrative analytical framework rather than a traditional phylogeographic approach. Molecular phylogenetic and population genetic analyses rely on sequences from GenBank, while distribution models use occurrence records from GBIF and published literature. Both datasets are organized at the species level; accordingly, the coupling between genetic and distributional patterns is explored through species-level associations, rather than through direct gene–geography links at the individual or population level.

### 2.2. Phylogeny Inference

Based on the 255 mitogenome sequences, two datasets were extracted: (1) the 13 protein-coding genes (PCGs: *ATP6*, *ATP8*, *COI*, *COII*, *COIII*, *Cytb*, *ND1*, *ND2*, *ND3*, *ND4*, *ND4L*, *ND5*, *ND6*) and (2) 15 genes (13 PCGs + *12S rRNA* and *16S rRNA*). The 13 PCGs were aligned individually using MAFFT v7.0 [35] under codon alignment mode, while the 15 genes were aligned under the default mode. Base saturation was assessed according to Xia et al. (2018) in DAMBE v7.3.11 [36]. And these alignments were concatenated by species in PhyloSuite v1.2.3 [37] to form a supermatrix. The optimal partitioning scheme and best-fit nucleotide substitution models (Table S4) were determined with PartitionFinder v2.1.1 [38], applying a partition strategy by gene and codon position and selecting the best scheme using the corrected Akaike Information Criterion (AICc).

Phylogenetic trees were inferred using two approaches: Maximum Likelihood (ML) in IQ-TREE v2.0 [39] with 1000 ultrafast bootstrap replicates [40], and Bayesian Inference (BI) in MrBayes v3.2.7 [41] with two independent MCMC runs of 2 × 10^7^ generations, sampling every 2000 generations and discarding the first 25% as burn-in. Convergence was assessed in Tracer v1.7 [42] (ESS > 200). Bootstrap values ≥ 70% or posterior probabilities ≥ 0.95 were regarded as strong support. Tree visualization and annotation were performed in FigTree v1.4.4 and iTOL v6 [43].

### 2.3. Population Genetics

A total of 746 *COI* and 877 *cytb* sequences of the 12 *Triplophysa* species were compiled from GenBank (see Section 2.1; Table S2). These sequences served as the basis for the following analyses.

#### 2.3.1. Genetic Diversity

Genetic diversity parameters were calculated for each species/population using DnaSP v6 [44], including: (1) haplotype diversity (Hd), (2) nucleotide diversity (π), and (3) the number of polymorphic sites (S). 

#### 2.3.2. Population Structure

Population genetic structure was assessed using the following approaches: (1) Analysis of molecular variance (AMOVA) implemented in Arlequin v3.5 [45] to calculate pairwise population differentiation indices (*F*_ST_). These results were further integrated with the phylogenetic tree and haplotype network described below to elucidate the relationships among species and populations.

#### 2.3.3. Haplotype Network Reconstruction

Haplotypes were identified separately for COI and cytb using DnaSP v6, and haplotype networks were constructed for each gene using the Median-Joining (MJ) algorithm [46] in PopART v1.7 [47]. In the network, each circle represents a haplotype, with its size proportional to the number of individuals carrying it, and connecting lines indicate the number of mutational steps. This network provides an intuitive visualization of the genealogical relationships and genetic differentiation among the 12 *Triplophysa* species.

#### 2.3.4. Phylogenetic Tree Inference

The *cytb* and *COI* sequences were aligned separately using MAFFT with the L-INS-i (accurate) algorithm, implemented in PhyloSuite v1.2.3. Ambiguously aligned positions were trimmed with Gblocks v1.0 [48] under less stringent settings. The optimal partitioning scheme and best-fit substitution models were selected using ModelFinder [49] based on the corrected Akaike Information Criterion (AICc). Phylogenetic trees were reconstructed using Maximum Likelihood and Bayesian Inference, following the procedures described above.

Unrooted phylogenetic networks were reconstructed separately for *COI* and *cytb* in SplitsTree v6.0 [50], as the GenBank-derived sequences were not necessarily from the same individuals and therefore could not be concatenated. The Neighbor-net was applied with Kimura 2-parameter distances. Outgroup taxa were excluded to obtain an unrooted representation of relationships among the 12 *Triplophysa* species.

To quantify genetic differentiation, pairwise genetic distances were calculated from the gene *COI* using the Kimura 2-parameter (K2P) model in MEGA 11 [51]. Mean intraspecific distances were computed for all species with multiple individuals, and mean interspecific distances were obtained for all species pair.

#### 2.3.5. Demographic History

Population demographic history was inferred using the following combined approaches: (1) Neutrality tests (Tajima’s *D* and Fu’s *F*s) calculated in DnaSP v6.0; (2) Bayesian Skyline Plot (BSP) analysis conducted in BEAST v2.6.7 to reconstruct the trajectory of effective population size changes over time. BSP analyses were performed separately for *COI* and *cytb* under an uncorrelated lognormal relaxed clock, with substitution rates fixed at 1.5 × 10^−8^ and 1.0 × 10^−8^ substitutions/site/year, respectively, consistent with the mean rate for fish mitochondrial protein-coding genes. Best-fit substitution models were selected using ModelFinder. The coalescent Bayesian Skyline prior was applied. Four independent MCMC runs of 20 million generations were performed, sampling every 2000 generations and discarding the first 10% as burn-in; convergence was confirmed in Tracer v1.7 (ESS > 200 for all parameters). No internal calibration was applied; time estimates rely solely on the assumed substitution rates and should therefore be treated as approximate.

### 2.4. Species Distribution Modeling

Occurrence records for the 12 *Triplophysa* species were obtained from GBIF and published literature and then subjected to the following filtering steps: (1) duplicate records and points with obviously erroneous coordinates were removed; (2) spatial thinning was performed using the R package spThin with a minimum distance threshold (approximately 10 km), whereby one point was randomly retained when the geographic distance between two records fell below the threshold; (3) only species with ≥10 valid occurrence points were retained, as MaxEnt maintains high predictive accuracy even with small sample sizes [52,53]. Nineteen bioclimatic variables (bio1–bio19) were downloaded from the WorldClim database (http://www.worldclim.org/, accessed on 21 May 2026). A multi-step variable selection was conducted in R: (1) Pearson correlation analysis was used to calculate pairwise correlation coefficients, and among variables with |r| ≥ 0.8, the one with lower contribution was removed; (2) variance inflation factor (VIF) diagnostics were performed using the vifstep function in the usdm package, and variables with VIF > 10 were excluded; (3) variable importance was evaluated using the randomForest package, and variables with low importance scores were removed in conjunction with ecological considerations.

With the retained occurrence points and environmental variables, species distribution models were constructed using MaxEnt v3.4.1 [54]. Feature combinations (FC), including linear (L), quadratic (Q), product (P), threshold (T), and hinge (H) features, were selected based on sample size and the spatial arrangement of occurrence points. The regularization multiplier was set to 1, and the maximum number of background points was set to 10,000. Twenty-five percent of the data were randomly selected as the test set to validate the model. The bootstrap method was used as the resampling procedure, with 10 replicate runs per species, and the output format was logistic probability. The maximum number of iterations was set to 500, with a convergence threshold of 0.00001 and a random seed applied. Model performance was evaluated using the area under the receiver operating characteristic curve (AUC): (1) AUC > 0.9 was considered excellent, and 0.8–0.9 good [55]; (2) the importance of each environmental variable was assessed using jackknife tests. The optimized models calibrated on current conditions were then projected onto three paleoclimatic layers—the Last Interglacial (LIG), the Last Glacial Maximum (LGM), and the Mid-Holocene (MHO)—to reconstruct potential distribution patterns under different climatic scenarios.

### 2.5. Integrated Analysis of Population Genetics and Distribution

To examine the relationship between genetic diversity and distribution range size, species-level genetic diversity indices—haplotype diversity (Hd) and nucleotide diversity (π)—were correlated with the current extent of suitable habitat derived from MaxEnt models. For each of the 12 species, the continuous logistic-probability output was imported into ArcGIS v10.8 (ESRI) and converted to a raster. To convert the continuous logistic probabilities into discrete suitability classes, we examined the range of logistic values at training occurrence points across all 12 species. The minimum probability covering 90% of the training points was used as the lower bound for suitable habitat, while the remaining probability range was divided into moderate and high suitability. Accordingly, the raster was reclassified into three classes: low suitability (logistic probability < 0.38), moderate suitability (0.38–0.62), and high suitability (0.62–0.99).

After projecting to an equal-area projection (Asia North Albers Equal Area Conic), the areas of the moderate- and high-suitability classes were calculated using the Tabulate Area tool. To assess the robustness of the diversity–range relationships, we conducted two complementary sets of correlation analyses. In the primary analysis, *T. siluroides* and *T. hsutschouensis* were excluded because their extremely low nucleotide diversity (π < 0.007) despite moderate range size probably reflects recent genetic bottlenecks or founder effects, which could confound the association between range size and genetic diversity. In a sensitivity analysis, all 12 species were included to evaluate whether the exclusion of these two taxa influenced the overall patterns. Suitable habitat areas were log_10_-transformed prior to analysis to correct for right-skewed distribution. Pearson and Spearman correlation coefficients were then computed in R v4.3 (R Core Team, 2024) to assess associations between these area variables and genetic diversity estimates, testing the hypothesis that species with larger geographic ranges harbour higher genetic diversity.

## 3. Results

### 3.1. Phylogenetic Inference of Triplophysa

**Figure 1:**
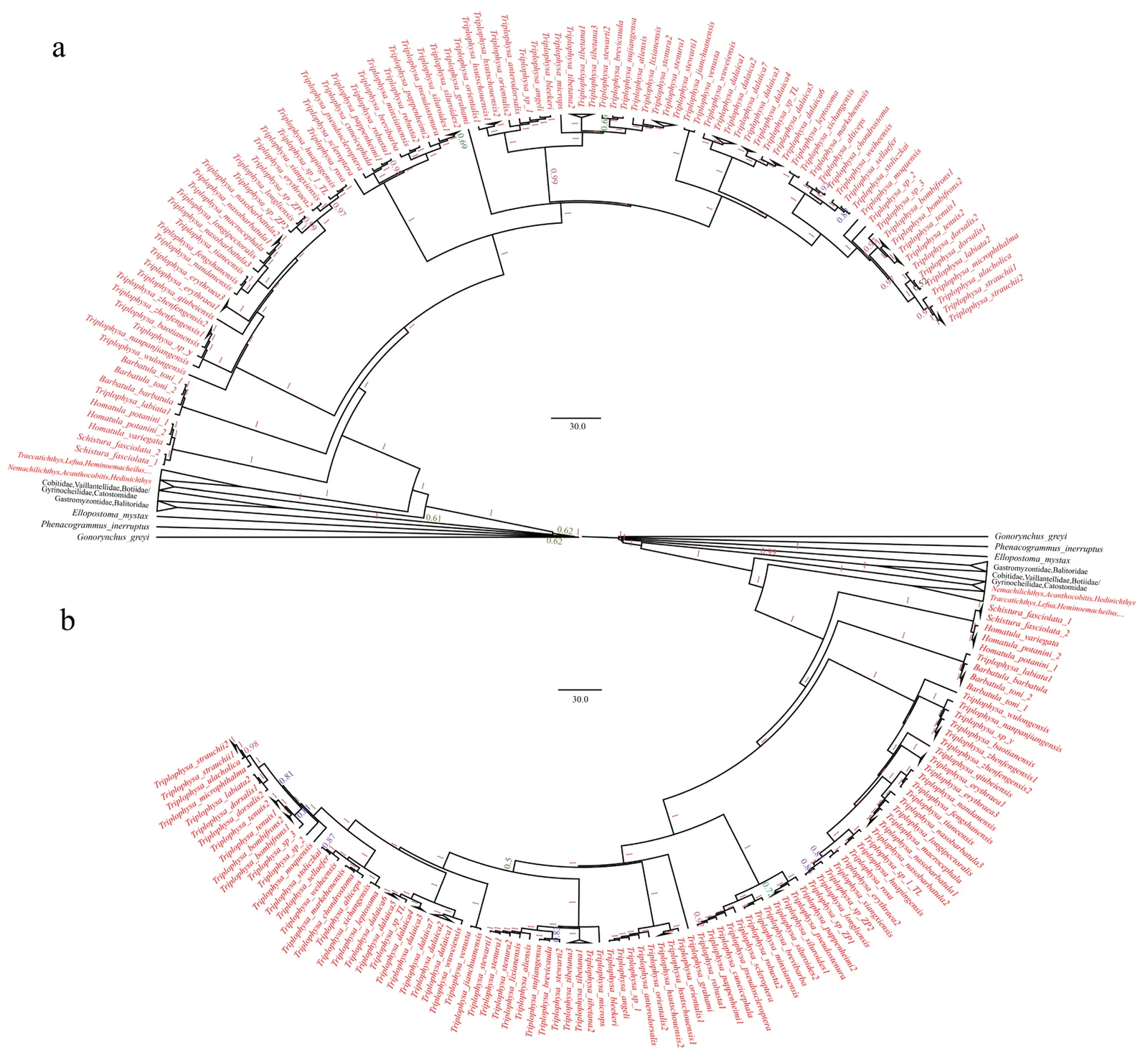
Bayesian phylogenetic trees of *Triplophysa* inferred from (**a**) 15 mitochondrial genes and (**b**) 13 protein-coding genes (PCGs). Red represents species of Nemacheilidae. Branche labels indicate posterior probabilities. Scale bars represent substitutions per site.

For both ML and BI trees, the 13-PCG and 15-gene topologies were largely congruent, but node support differed between datasets: the 13-PCG dataset consistently received higher support at deep nodes than the 15-gene dataset. The 15-gene tree (Figure 1a) exhibited lower support at several deep nodes, with posterior probabilities around 0.60, whereas the 13-PCGs tree (Figure 1b) showed distinctly higher deep-node support, with posterior probabilities mostly reaching 1.00. Within *Triplophysa*, internal node support varied locally: the clade (*T. stewarti*, *T. brevicauda*, *T. nujiangensa*) received PP = 0.87 (13-PCG) vs. 0.66 (15-gene), whereas (*T. xiangxiensis*, *T.* sp.1, *T. huapingensis*, *T. rosa*) was PP = 0.97 (15-gene) vs. 0.87 (13-PCG).

In the Bayesian tree inferred from the 13 PCGs, all major *Triplophysa* clades received PP = 1.00 except one (0.50); only eight shallow nodes had PP between 0.50 and 0.95, with the rest reaching 1.00. In contrast, the 15-gene Bayesian tree showed no major clade with such low support; all major clades received PP > 0.95. The ML tree was highly congruent with the BI topology, although branch support was generally lower than that of the Bayesian tree (Figure S1). Notably, multiple conspecific individuals, including those from the same locality, failed to form monophyletic groups. For instance, of the three *T. erythraea* specimens, one was placed very close to *T. xiangxiensis* rather than clustering with the other two (Figure 1). This could reflect taxonomic misidentification in public databases (GenBank), historical mitochondrial capture, or a combination of both. The genus was clearly split into two major lineages—cave-dwelling and non-cave-dwelling—which diverged from the most recent common ancestor.

The phylogenetic placements of these 12 species (except for *T. obscura*) addressed in this study were as follows: *T. siluroides* was recovered as the sister taxon to *T. pseudostenura*; *T. robusta*, *T. minxianensis*, and *T. brevicauda* formed a clade, with divergent *T. robusta* individuals; *T. orientalis* was placed as sister to *T. hsutschouensis* (also with intraspecific divergence); *T. dalaica* showed pronounced intraspecific divergence; *T. bleekeri* was recovered as the closest relative of *T. angeli*; *T. brevicauda* was sister to *T. nujiangensa*; *T. stenura* grouped with *T. stewarti* and *T. lixianensis*; *T. leptosoma* was recovered as the sister taxon to *T. xichangensis*; *T. stoliczkae* was placed as sister to *T. sellaefer*. All these relationships received high statistical support (Figure 1).

### 3.2. Population Genetic Diversity and Structure of These 12 Triplophysa Species

#### 3.2.1. Population Genetic Diversity

After alignment, the *COI* gene sequence was 840 bp in length, and the *cytb* gene sequence was 1141 bp. Genetic diversity varied considerably among the 12 *Triplophysa* species (Table 1). *T. leptosoma* and *T. stoliczkae* exhibited the highest nucleotide diversity (π: *COI* 0.0530 and 0.0542, *cytb* 0.0694 and 0.0901, respectively) together with high haplotype diversity (Hd > 0.95). *T. bleekeri* and *T. brevicauda* also showed high Hd (>0.93) and high *COI* π (0.0422–0.0509) but moderate *cytb* π (0.0245–0.0296). *T. robusta* and *T. stenura* had high Hd (>0.92) and moderate π (0.0123–0.0184); *T. obscura*, represented only by *COI*, fell within the same range (Hd = 0.996, π = 0.0136). *T. siluroides* and *T. hsutschouensis* displayed moderate to high Hd (0.874–0.933) but low π (<0.007). *T. dalaica* was the only species with low Hd (<0.6) yet moderate π (Table 2). Discordant marker patterns were evident in *T. minxianensis* (*COI*: Hd = 1.000, *cytb*: Hd = 0.879; low π in both) and *T. orientalis* (*COI*: π = 0.0569; *cytb*: Hd = 1.000, π = 0.0037).

#### 3.2.2. Population Genetic Structure

Each species was treated as a single group for the purpose of AMOVA; thus, the analysis partitions genetic variation among species (i.e., interspecific divergence). AMOVA revealed that the majority of genetic variation resided among species (*COI*: 73.96%; *cytb*: 88.65%; Table 2), with pairwise *F*_ST_ values ranging from 0.2012 to 0.9333 for *COI* and from 0.3702 to 0.9881 for *cytb* (Table S5).

**Figure 2:**
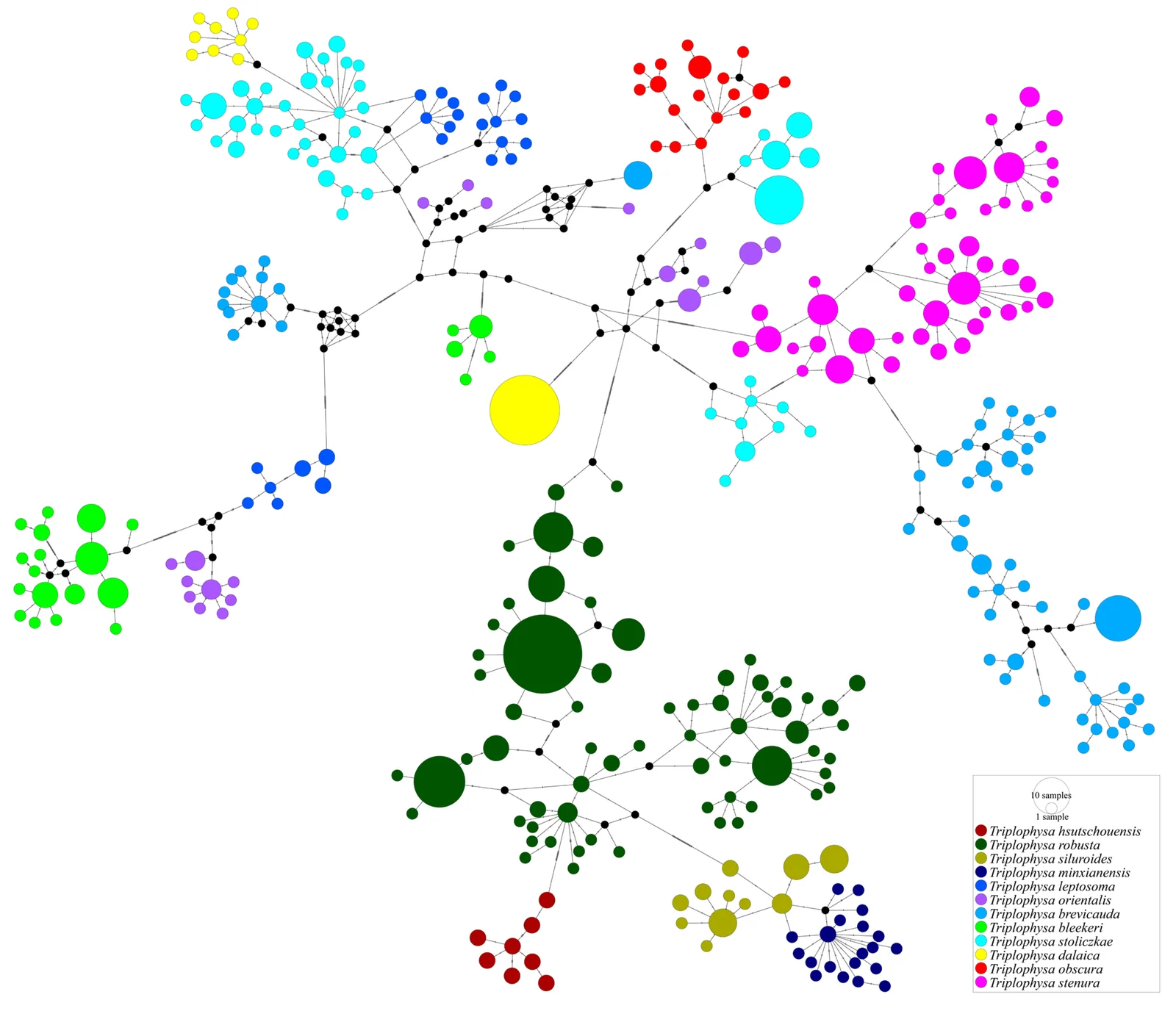
Median-joining haplotype network of the 12 *Triplophysa* species based on *COI* sequences. Each circle represents a distinct haplotype, with circle size proportional to haplotype frequency; colors correspond to species. Small black circles indicate median vectors (unsampled or extinct haplotypes). Each connecting line represents a single nucleotide substitution, and each little short line represents mutated position.

Haplotype networks based on *COI* and *cytb* revealed species-specific haplogroups with no shared haplotypes, indicating strong interspecific genetic differentiation. Mutational divergence between species was generally high, except that *T. siluroides* and *T. minxianensis* were separated by very few steps in both *COI* MJ (Figure 2) and *cytb* TCS networks (Figure S2). Apart from these two, all species showed pronounced intraspecific divergence, with multiple mutational steps among conspecific haplotypes.

*Triplophysa minxianensis* displayed a high Hd, low π pattern (*COI* Hd = 1, π = 0.0040; *cytb* π = 0.0025, Table 1) indicative of recent expansion, a pattern also shared by *T. siluroides* and *T. hsutschouensis*. By contrast, *T. orientalis* showed discordant signals: high π in *COI* (π = 0.0569, Hd = 0.961) but extremely low π in *cytb* (π = 0.0037, Hd = 1.000).

**Figure 3:**
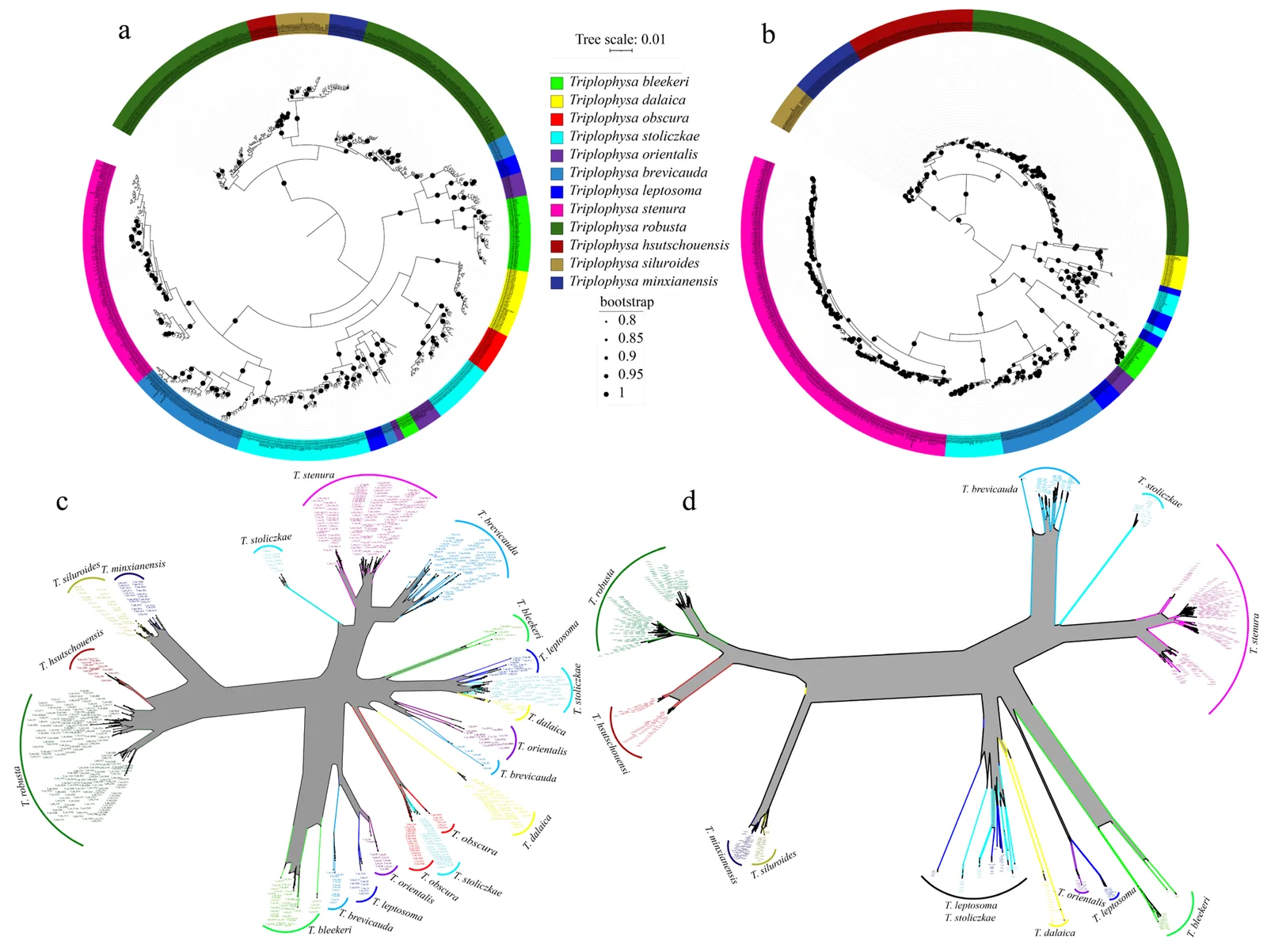
Mitochondrial gene trees and genetic clustering among 12 *Triplophysa* species based on mitochondrial *COI* and *cytb* genes. (**a**) Bayesian inference (BI) tree constructed from *COI* sequences; (**b**) BI tree from *cytb* sequences; SplitsTree network based on K2P genetic distances from *COI* (**c**) and *cytb* (**d**) sequences. Node labels indicate posterior probabilities (PP); species are color-coded as shown in the legend.

Mitochondrial gene trees strongly corroborated the haplotype results, with no interspecific admixture. Six species—*T. stenura*, *T. dalaica*, *T. siluroides*, *T. obscura*, *T. hsutschouensis*, and *T. minxianensis*—were reciprocally monophyletic in both gene trees (Figure 3a,b; Figure S3). The remaining species showed paraphyletic or polyphyletic relationships. Subclades of *T. leptosoma*, *T. stoliczkae*, *T. brevicauda*, *T. orientalis*, and *T. bleekeri* were recovered in several mixed clusters: one *T. leptosoma* subclade grouped with *T. stoliczkae*; another with *T. orientalis*; a *T. stoliczkae* subclade clustered with *T. brevicauda* and *T. stenura*; and subclades of *T. leptosoma*, *T. brevicauda*, *T. orientalis*, and *T. bleekeri* together formed an additional mixed cluster. *T. robusta* was split into two clades in the *COI* tree, one of which clustered with *T. hsutschouensis*, *T. siluroides*, and *T. minxianensis*, whereas it was monophyletic in the *cytb* tree.

SplitsTree analysis based on K2P genetic distances further supported these phylogenetic patterns (Figure 3c,d). The six reciprocally monophyletic species formed well-separated clusters with clear genetic differentiation in the SplitsTree network. *T. robusta* and *T. stenura* each formed distinct branches, separate from other species. Among the remaining paraphyletic or non-monophyletic species, *T. leptosoma*, *T. stoliczkae*, *T. brevicauda* and *T. orientalis* were distributed across multiple adjacent clusters without forming complex reticulate networks. *Triplophysa siluroides* and *T. minxianensis* clustered together, whereas different lineases of *T. leptosoma* formed separate clusters with *T. stoliczkae* and *T. orientalis*, respectively. Overall, the SplitsTree analysis is consistent with the phylogenetic results, with non-monophyletic species forming multiple adjacent but separate clusters; however, given the strictly matrilineal inheritance of mitochondrial DNA, this pattern cannot be interpreted as evidence against historical introgression or reticulate evolution.

Based on the *COI* gene, mean intraspecific genetic distances ranged from 0.0040 (*T. minxianensis*) to 0.0614 (*T. orientalis*) (Table S6). Other species with low intraspecific distances included *T. siluroides* (0.0046) and *T. hsutschouensis* (0.0058). Species with relatively high intraspecific distances included *T. leptosoma* (0.0581), *T. stoliczkae* (0.0575), and *T. brevicauda* (0.0539).

Mean interspecific distances ranged from 0.0076 (*T. siluroides* vs. *T. minxianensis*) to 0.1451 (*T. hsutschouensis* vs. *T. leptosoma*) (Table S6). Two species pairs showed relatively low interspecific distances: *T. siluroides* vs. *T. minxianensis* (0.0076 ± 0.0021), *T. robusta* vs. *T. hsutschouensis* (0.0351 ± 0.0058). Most species pairs showed interspecific distances substantially exceeding intraspecific distances.

#### 3.2.3. Demographic History

Based on BSPs reconstructed from *COI* and *cytb* sequences, the demographic histories of twelve *Triplophysa* species were inferred. Both markers yielded largely consistent signals across the majority of taxa (Figure S4a). *Triplophysa leptosoma* and *T. brevicauda* each maintained a stable effective population size prior to ~0.5 Ma, followed by a slight contraction that lasted until ~0.12 Ma and a subsequent rapid expansion. The BSP profile for *T. stoliczkae* indicated a stable effective population size before ~1.0 Ma, followed by a decline at ~0.12 Ma and a rapid expansion thereafter. *Triplophysa robusta* and *T. stenura* exhibited long-term stability with recent, pronounced expansions initiating at ~0.12 Ma and ~0.17 Ma, respectively. *T. minxianensis* and *T. siluroides* displayed relatively constant effective population sizes throughout the examined period, with no evidence of significant demographic expansions or contractions. *T. bleekeri* maintained a stable overall trend, with a mild contraction between ~0.4 and 0.1 Ma followed by a slight expansion. For the remaining four species, *T. hsutschouensis*, *T. dalaica*, and *T. orientalis*, minor discrepancies between the two markers were observed in some details; however, the relative magnitude and direction of effective population size changes showed no fundamental conflict, and the overall concordance between *COI* and *cytb* skyline trajectories remained predominant; while *T. obscura* was only resolved by *COI* (Figure S4b).

### 3.3. Species Distribution Models of Triplophysa Species

After data cleaning, effective occurrence localities of *Triplophysa* species are listed in Table 3 and were used to compare current and historical distributions of the 12 species. Following multi-step variable selection in R, bio1 and bio16 were excluded, and the remaining 17 bioclimatic variables were retained for MaxEnt modeling, with species-specific subsets detailed in Table 3. Mean AUC values exceeded 0.85 for *T. brevicauda* and *T. orientalis* and 0.90 for the other ten species.

Based on the jackknife tests of AUC on test data (Figure S5), the most influential variable when used alone for each species was identified as: bio4 for *T. bleekeri*; bio3 for *T. brevicauda*, *T. hsutschouensis* and *T. stoliczkae*; bio5 for *T. dalaica*; bio2 for *T. leptosoma*, *T. orientalis*, and *T. siluroides*; bio15 for *T. minxianensis*, *T. obscura*, and *T. robusta*; and bio5 for *T. stenura*. The jackknife test of variable importance further revealed that the omission of specific variables resulted in the largest AUC declines as follows: bio4 (*T. bleekeri*), bio14 (*T. brevicauda*), bio5 (*T. dalaica*, *T. stenura*, and *T. robusta*), bio3 (*T. hsutschouensis*), bio19 (*T. leptosoma*), bio2 (*T. orientalis* and *T. siluroides*), and bio15 (*T. minxianensis*, *T. stoliczkae*, and *T. obscura*).

#### 3.3.1. Distribution of Potentially Suitable Habitats in the Current Period

**Figure 4:**
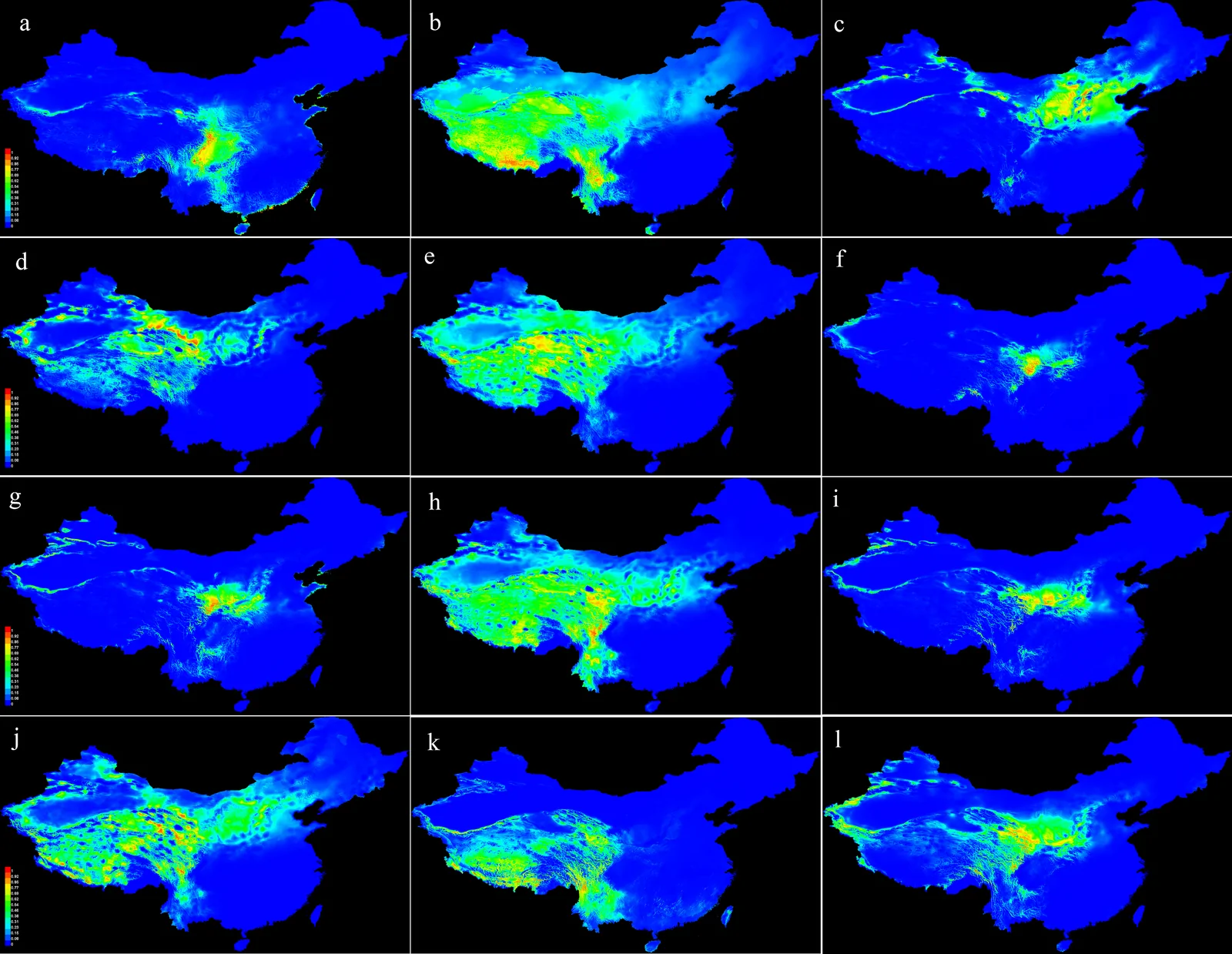
Current MaxEnt potential habitat models for the 12 *Triplophysa* species. (**a**) *T. bleekeri*; (**b**) *T. brevicauda*; (**c**) *T. dalaica*; (**d**) *T. hsutschouensis*; (**e**) *T. leptosoma*; (**f**) *T. minxianensis*; (**g**) *T. obscura*; (**h**) *T. orientalis*; (**i**) *T. robusta*; (**j**) *T. siluroides*; (**k**) *T. stenura*; (**l**) *T. stoliczkae*. Note: Red and yellow areas denote core potential habitats, green and cyan areas denote marginal potential habitats, and blue areas indicate unsuitable regions.

MaxEnt models predicted potential suitable habitats that were broadly consistent with documented occurrence records (Figure 4 and Figure S6). The 12 species were grouped into five ecogeographic groups based on their potential habitat patterns. The Qinghai–Tibet Plateau widespread group (*T. brevicauda*, *T. leptosoma*, *T. orientalis*) had the largest core habitats across the main plateau, western Sichuan Plateau, and Gannan Plateau; secondary habitats reached northwestern Yunnan via the Hengduan Mountains and the eastern Hexi Corridor and Qilian piedmont. 

The upper-river endemic group (*T. bleekeri*, *T. minxianensis*, *T. robusta*, *T. obscura*, *T. siluroides*, *T. stoliczkae*) was the most narrowly distributed. *T. bleekeri* and *T. minxianensis* were largely confined to the Qinling–Minshan mountains, *T. obscura* and *T. siluroides* to the Gannan–western Loess Plateau along the upper Yellow River, and *T. robusta* displayed an east–west banded pattern from the Qinling–Taihang Mountains to the northeastern plateau, linking the Loess and Qinghai–Tibet Plateaus via a corridor along the northern Qinling, southern Taihang, and Qilian Mountains; *T. stoliczkae* spanned more broadly from the upper Yellow River to the northeastern plateau margin. In all six species, secondary habitats expanded only marginally along watercourses.

The semi-arid northern plateau endemic *T. dalaica* concentrated in lake clusters of central-eastern Inner Mongolia and the West Liao and Luan basins, forming a belt along the Yinshan–Yanshan ranges, with secondary habitats covering the central Inner Mongolian and northern Loess Plateaus.

*T. hsutschouensis*, an arid northwest endemic, formed narrow core belts along the East Tianshan–eastern Xinjiang–Hexi–Qilian corridor, with secondary habitats extending to the margins of Xinjiang’s major basins, the Alxa Plateau, Qilian Mountains, and Qaidam Basin periphery, and being nearly absent from eastern plains.

The Hengduan longitudinal valley group (*T. stenura*) tracked the north–south drainages, with ribbon-like core habitats along the Jinsha, Lancang, and Nu Rivers and tributaries, and secondary habitats covering northwestern Yunnan, western Sichuan, southeastern Tibet, and the western Yunnan–Guizhou Plateau.

#### 3.3.2. Paleoclimatic Species Distribution Modeling

**Figure 5:**
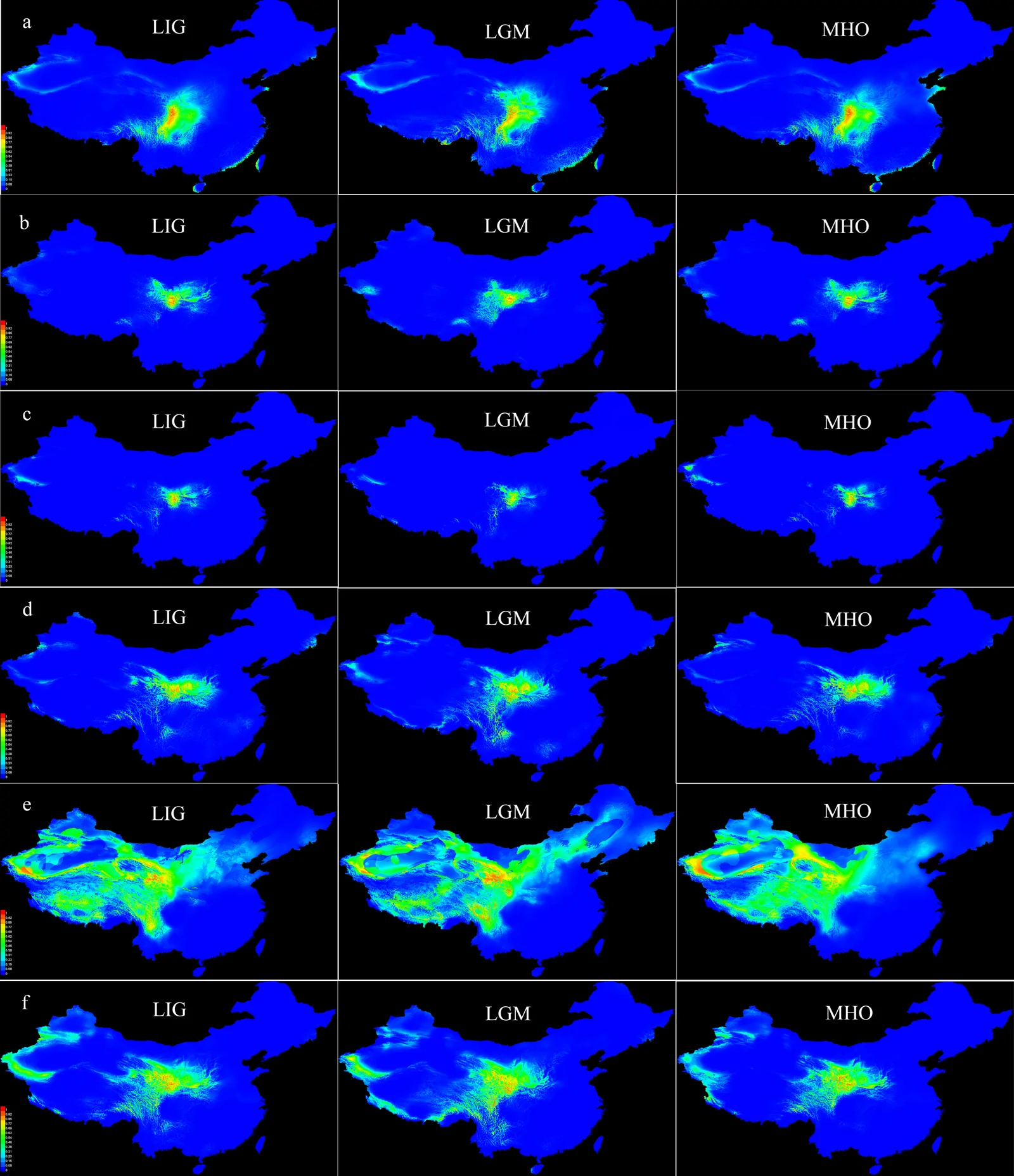
Paleoclimatic MaxEnt potential habitat models for the upper-river endemic group of *Triplophysa.* (**a**) *T. bleekeri*; (**b**) *T. minxianensis*; (**c**) *T. obscura*; (**d**) *T. robusta*; (**e**) *T. siluroides*; (**f**) *T. stoliczkae*.

**Figure 6:**
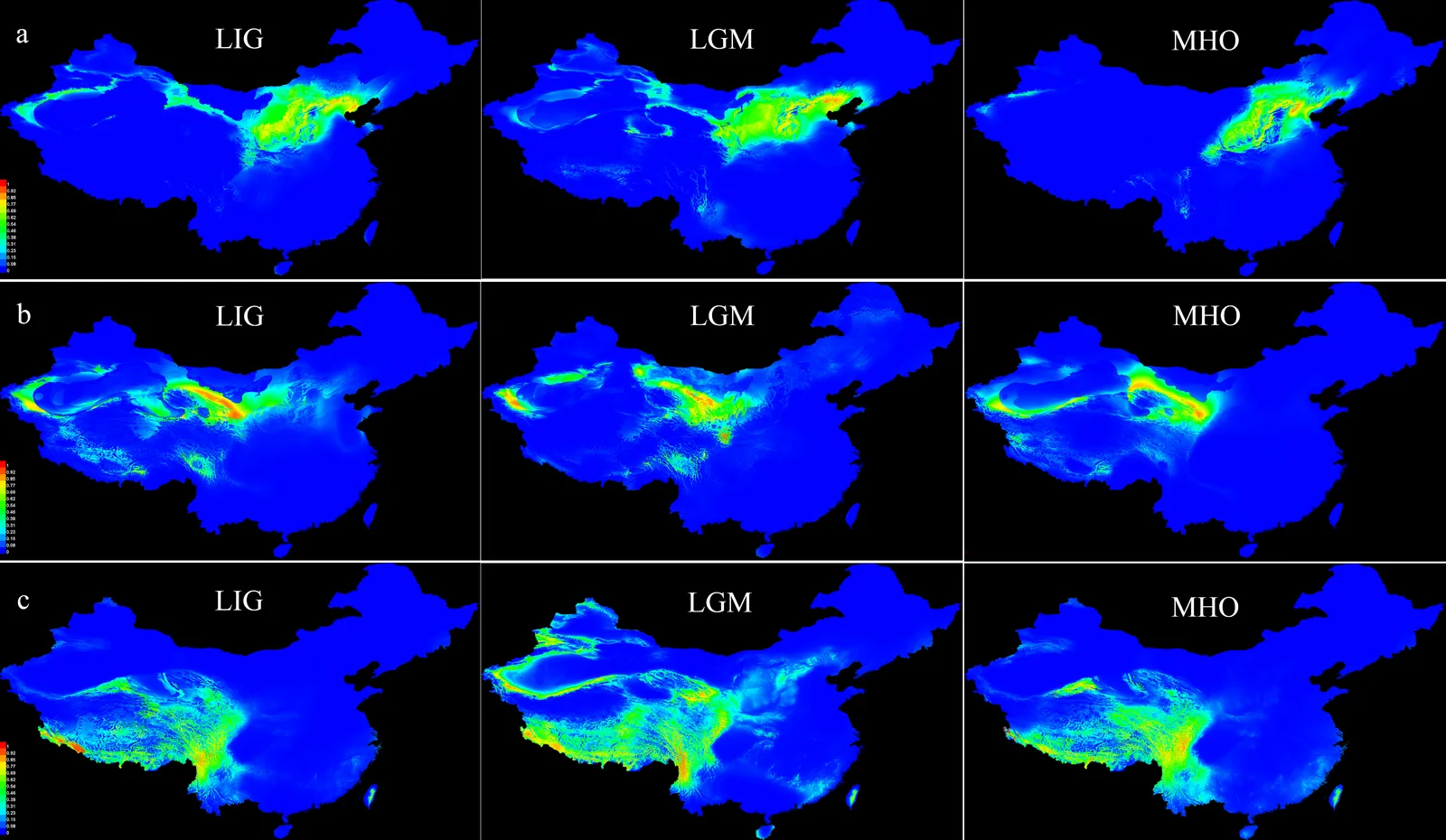
Paleoclimatic MaxEnt distribution models for three narrow-ranged endemic *Triplophysa* species. (**a**) *T. dalaica*; (**b**) *T. hsutschouensis*; (**c**) *T. stenura*.

**Figure 7:**
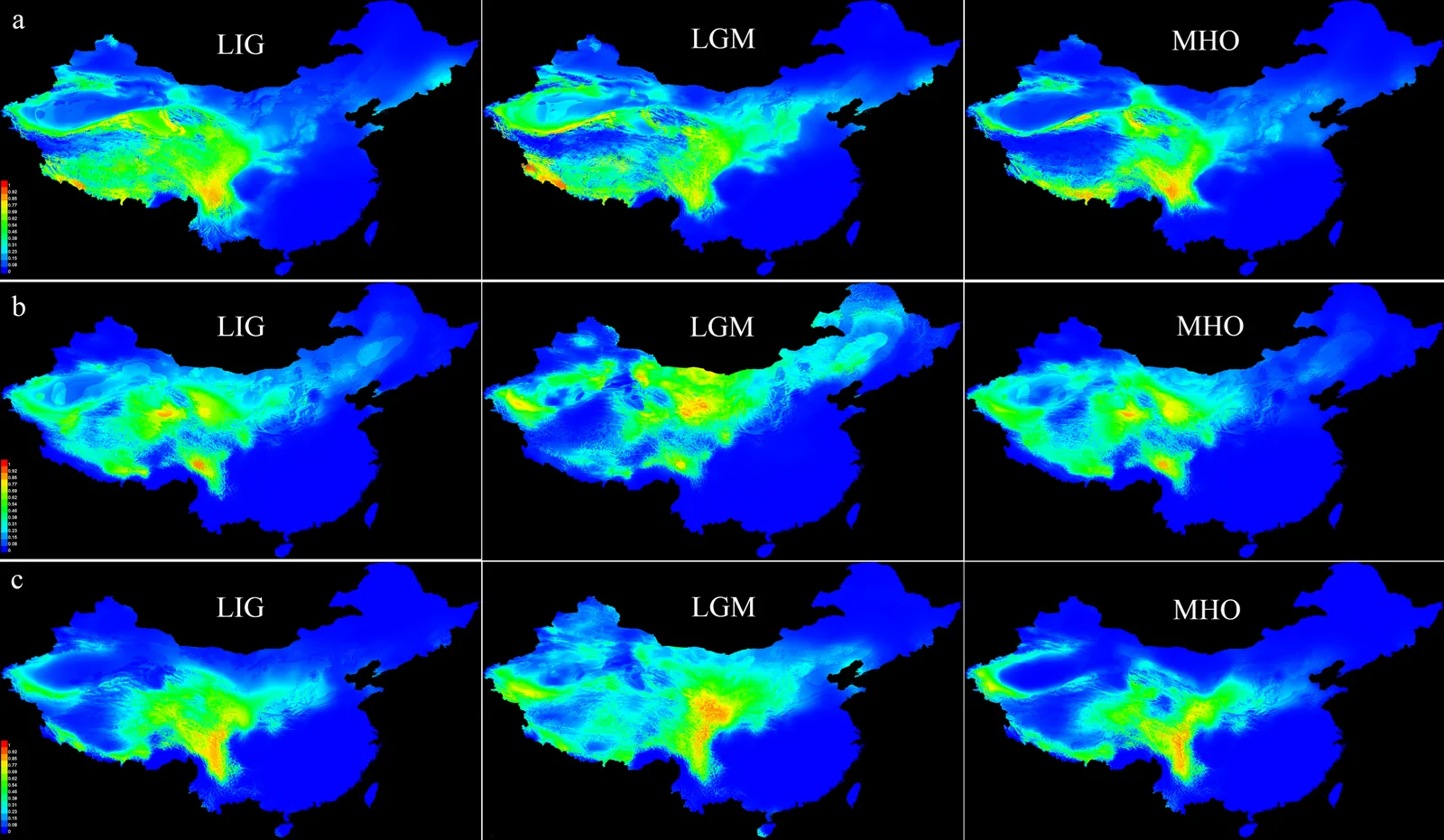
Paleoclimatic MaxEnt distribution models for the plateau-widespread group. (**a**) *T. brevicauda*; (**b**) *T. leptosoma*; (**c**) *T. orientalis*.

Paleoclimatic reconstructions across three key periods (LIG, LGM, MHO) revealed that the potential habitat dynamics of all 12 *Triplophysa* species closely tracked glacial–interglacial climate fluctuations: suitable habitats were most restricted and fragmented during warm periods (LIG, MHO) and expanded into continuous ranges during the cold LGM (Figure 5, Figure 6 and Figure 7).

During the LIG, species persisted as isolated refugia. The upper-river group was restricted to the central Loess Plateau, central Gansu, and northern Qinling foothills (Figure 5); *T. stoliczkae* additionally reached the northeastern plateau and eastern Hexi Corridor, and *T. robusta* showed a two-center pattern (central Loess and northeastern plateau). The core suitable habitats of plateau-widespread and Hengduan groups were largely confined to the eastern plateau margin and southern Hengduan Mountains. *T. hsutschouensis* and *T. dalaica* retained only narrow belts along the northeastern plateau margin and eastern Loess Plateau (Figure 6), respectively. During the LGM, habitats expanded into broad, continuous belts. The upper-river group formed a Loess Plateau–North China belt reaching western North China and southern Inner Mongolia. The plateau-widespread and Hengduan groups occupied the central-eastern and southern plateau and the entire Hengduan Mountains with high connectivity (Figure 7). Beyond the Loess and Inner Mongolian Plateaus, *T. dalaica* extended into the Northeast China Plain. While the *T. hsutschouensis* formed three separate suitable habitat patches in eastern Xinjiang, the Hexi Corridor, and the Loess Plateau–southern Inner Mongolia region. 

During the MHO, warming caused widespread contraction and fragmentation. The upper-river group retreated to the central Loess Plateau, eastern Gansu–northern Shaanxi, and northern Qinling foothills; *T. robusta* retained a larger area in the northeastern plateau. The plateau-widespread and Hengduan groups shrank to the eastern and southeastern plateau margin and northern Hengduan Mountains. The core suitable habitats of *T. dalaica* became more fragmented yet retained a narrow belt from the northeastern plateau to central-eastern Inner Mongolia, whereas *T. hsutschouensis* reverted to narrow belts along the northeastern plateau margin as in the LIG, but more restricted.

Notably, *T. obscura* showed no marked range shifts across the LIG, LGM, and MHO, with its core suitable habitat persistently confined to the upper Yellow and Bailong watersheds and always smaller than the current range.

### 3.4. Correspondence Between Population Genetics and Distribution Patterns

In the primary analysis excluding *T. siluroides* and *T. hsutschouensis*, *COI* haplotype diversity (Hd) was not significantly correlated with distribution area (*p* > 0.05), whereas π showed a significant correlation with area (Pearson r = 0.776, *p* = 0.0084; Spearman ρ = 0.776, *p* = 0.0242). For *cytb*, π was not significantly correlated with area (Pearson *p* = 0.4227; Spearman *p* = 0.7081), but Hd displayed a discrepancy between the Pearson (r = 0.206, *p* = 0.5945) and Spearman (ρ = 0.828, *p* = 0.0058) correlations.

In the sensitivity analysis including all 12 species, the associations generally weakened: *COI* Hd (Pearson r = 0.074, *p* = 0.820; Spearman ρ = −0.123, *p* = 0.704), *COI* π (r = 0.561, *p* = 0.058; ρ = 0.566, *p* = 0.059), *cytb* Hd (r = 0.235, *p* = 0.486; ρ = 0.597, *p* = 0.053), and *cytb* π (r = 0.118, *p* = 0.730; ρ = 0.109, *p* = 0.755) were all non-significant at *p* < 0.05, with only *cytb* Hd approaching significance under Spearman correlation. 

## 4. Discussion

This study integrated mitochondrial phylogenomics, population genetics, and ecological niche modeling to elucidate the coupling between phylogenetic relationships, genetic diversity, and distribution patterns across 12 *Triplophysa* species, ranging from widespread to narrow-range endemics. *Triplophysa* was recovered as monophyletic, with a clear split into cave-dwelling and non-cave-dwelling lineages. Although these inferences are based on mitochondrial gene trees, which may not always correspond to species trees, they are consistent with prior mitogenomic work; however, our study is novel in integrating phylogenetic, population genetic, and distribution modeling approaches to compare widespread and narrow-range species. Genetic diversity correlated positively with range size. Widespread species expanded markedly during the LGM, while narrow-range endemics remained stable or declined, mirroring the glacial expansion and interglacial contraction of suitable habitats. These findings demonstrate that range size not only shapes genetic diversity but also fundamentally influences phylogenetic patterns and demographic histories.

### 4.1. Phylogenetic Reconstruction and Interspecific Divergence of Triplophysa

The phylogenetic trees reconstructed from the 13 protein-coding genes (PCGs) and the 15 mitochondrial genes were largely congruent in topology, but the 13-PCG dataset provided markedly higher support at deep nodes. This improvement likely arises from the exclusion of the *12S* and *16S rRNA* genes, which evolve slowly and can introduce phylogenetic noise due to saturation effects or alignment ambiguity [56,57,58]. In contrast, protein-coding genes exhibit more homogeneous substitution rates across codon positions and thus deliver a stronger phylogenetic signal for resolving deep divergences [58,59,60]. The high posterior probabilities (mostly 1.00) and bootstrap support values indicate that the inferred relationships are robust. Our mitogenomic phylogeny recovers *Triplophysa* as monophyletic, consistent with other mitogenome-based studies [7,61,62]. A single anomalous placement—*Triplophysa labiata*1 clustering within *Barbatula*—mirrors the pattern reported by Šlechtová et al. (2025) [63] and likely reflects taxonomic misidentification, whereas *Triplophysa labiata*2 was resolved correctly within *Triplophysa*. Although nuclear gene analyses have revealed pervasive cytonuclear discordance and extensive ancient introgression, with introgression contributing more to phylogenetic conflict than incomplete lineage sorting [9,13], these studies have not rejected the monophyly of the genus. Such widespread cytonuclear conflict introduces uncertainties in the taxonomic status of many morphologically defined species [9], suggesting that misidentification may not be uncommon. These discrepancies reflect differences in the phylogenetic information content between mitochondrial and nuclear markers, shaped by both ILS and gene flow [9,13,26]. 

Additionally, *Triplophysa* clearly splits into cave-dwelling and non-cave-dwelling lineages, a result strongly supported in our tree and corroborating earlier identifications of epigean and subterranean clades [10,11,64]. This bifurcation may suggest a single origin of the cave phenotype, followed by further diversification within the cave lineage.

Interspecific genetic distances further supported the above phylogenetic patterns. The intraspecific and interspecific K2P distances for *COI* display a clear distinction overall, consistent with the range reported for multiple *Triplophysa* species [8,65], confirming the utility of the *COI* marker in this group. However, two species pairs show anomalously low differentiation, revealing that mitochondrial gene trees can mask true evolutionary history. The interspecific distance between *T. siluroides* and *T. minxianensis* (0.0076, or 0.76%) is particularly noteworthy. This value falls well below the standard 2–3% *COI* barcoding threshold commonly used for species delimitation in teleost fishes and is even lower than the intraspecific distances of several widespread species (e.g., *T. orientalis* = 0.0614; *T. leptosoma* = 0.0581). This anomalously low divergence suggests one of three possibilities: (1) recent speciation, with insufficient time for mitochondrial lineage sorting; (2) ongoing or historical interspecific gene flow/introgression; or (3) questionable taxonomic validity of these two nominal species. We emphasize that this result is based on mitochondrial data alone; nuclear markers and morphological re-evaluation are needed to resolve the taxonomic status of *T. siluroides* and *T. minxianensis*. The distance between *T. robusta* and *T. hsutschouensis* (0.0351) is also among the lowest within the genus. These patterns align with molecular work on the *T. robusta* complex, which revealed that even in genetically depauperate lineages, widespread historical gene flow can cause cytonuclear discordance and dilute interspecific differentiation [66]. Therefore, a single-marker distance threshold may fail to delimit taxa with reticulate evolutionary histories, and integrative taxonomic testing with multiple nuclear genes is urgently needed.

Unsurprisingly, many species in our study were recovered as paraphyletic or polyphyletic. *Triplophysa leptosoma*, *T. stoliczkae*, *T. brevicauda*, *T. orientalis*, and *T. bleekeri* formed mixed clusters rather than reciprocally monophyletic groups, a result broadly consistent with other studies [8,66]. This pattern can arise from multiple processes: ILS due to recent divergence, mitochondrial capture via historical hybridization, or ongoing/historical gene flow among lineages [9,13,26,66]. Median-joining haplotype networks and SplitsTree analyses revealed no pronounced reticulate signals among the non-monophyletic species, a topology consistent with ILS. However, because these inferences rely exclusively on mitochondrial markers (*COI* and *cytb*), they cannot unambiguously discriminate ILS from historical introgression or mitochondrial capture—processes that can generate convergent genealogical patterns in single-locus datasets. Accordingly, while the observed topology does not contradict ILS, we refrain from invoking it as the predominant mechanism. Disentangling the relative contributions of these competing evolutionary scenarios will require genome-wide nuclear markers and coalescent-based hypothesis testing of gene flow versus ILS.

Multiple conspecific individuals, including those from the same locality, were not recovered as monophyletic. For instance, one of the three *T. erythraea* specimens grouped with *T. xiangxiensis* instead of its conspecifics. A possible explanation is species misidentification, especially for morphologically similar or recently described species [6,67,68]. Alternatively, mitochondrial capture through hybridization with sympatric relatives can cause species-level paraphyly or polyphyly, a phenomenon reported in over 20% of animal species [69]. Even moderate levels of introgression can mislead phylogenomic inference [12]. Although some relationships still require nuclear gene validation, this study provides the most comprehensive mitogenomic framework to date for widespread *Triplophysa*, clarifying the phylogenetic positions of cave-dwelling and non-monophyletic taxa and advancing our understanding of fish diversification on the Qinghai–Tibet Plateau.

### 4.2. Coupling Between Phylogeographic Structure and Distribution Patterns of Triplophysa

Five of the six monophyletic species—*T. stenura*, *T. dalaica*, *T. siluroides*, *T. hsutschouensis*, and *T. minxianensis*—exhibit essentially non-overlapping core distributions, even during the LGM range expansion (Figure 5, Figure 6 and Figure 7), demonstrating a tight correspondence between phylogenetic independence and allopatric distribution. *T. stenura* is restricted to the longitudinal valleys of the Hengduan Mountains, and its monophyly is tightly linked to long-term isolation imposed by the north–south drainages, consistent with previous findings that its phylogeographic structure strictly follows the longitudinal river systems and that its lineage divergence *coi*ncided with the Mid-Pleistocene climate transition [27]. *T. dalaica* is confined to the central-eastern Inner Mongolian Plateau, the West Liao River basin, and the Dali Lake area, and its monophyly points to isolation-driven divergence within the northern plateau lake cluster [70]. *Triplophysa siluroides* and *T. minxianensis* are distributed in the Gannan Plateau and western Loess Plateau along the upper Yellow River. They form a sister-group relationship with a K2P distance of only 0.0076, yet each retains reciprocal monophyly, corroborating earlier work that drainage dissection by the Qinling and Minshan Mountains promoted speciation without leading to mitochondrial sharing [8,23,66]. These monophyletic species share relatively narrow ranges, low habitat connectivity, and pronounced geographic isolation [26], conditions that facilitate lineage divergence and maintain genetic boundaries. The sixth monophyletic species, *T. obscura*, deserves special attention. Its core suitable habitat has remained consistently confined to the upper Yellow River and the Bailong River across all three paleoclimatic periods, and it forms a sister-group relationship with *T. stoliczkae* (Figure 3a). This pattern aligns closely with the convergence-driven taxonomic confusion documented for *T. stoliczkae* by Feng et al. (2019) [9], who demonstrated that morphologically similar *T. stoliczkae* individuals actually belong to genetically distinct lineages as a result of convergent evolution. However, the sister-group relationship between *T. obscura* and *T. stoliczkae* is more plausibly explained by shared mitochondrial ancestry or mitochondrial capture. Specifically, *T. obscura* may share a recent mitochondrial ancestor with one of the lineages within the *T. stoliczkae* complex, or it may have captured the mitochondrial genome of a *T. stoliczkae* lineage through historical hybridization. Morphological convergence cannot account for the molecular clustering of these two species in a mitochondrial gene tree. The upper Yellow River–Bailong River region likely acted as a long-term climatic refugium, enabling *T. obscura* to persist and evolve within a narrow niche. This stable area may harbor morphologically similar but genetically distinct cryptic species, a hypothesis that awaits testing through more detailed population genomic studies.

In contrast, five widespread species—*T. leptosoma*, *T. stoliczkae*, *T. brevicauda*, *T. orientalis*, and *T. bleekeri*—exhibit paraphyletic patterns *coi*nciding with their extensive ranges, with core habitats covering most of the QTP, the Qaidam Basin, the western Sichuan Plateau, and the Gannan Plateau (Figure 4, Figure 5, Figure 6 and Figure 7). Narrow-range endemics, with limited inter-population contact, rarely generate such topological conflicts (Figure 3 and Figure 4), whereas widespread species repeatedly experienced isolation and secondary contact during glacial–interglacial cycles, providing frequent opportunities for unidirectional mitochondrial introgression (mitochondrial capture) and causing cytonuclear discordance [71]. Our SplitsTree networks revealed no pronounced reticulation but rather multiple adjacent, well-delimited clusters (Figure 3c,d). However, because mitochondrial DNA is a single, non-recombining locus, the absence of reticulation in a SplitsTree network does not rule out introgression. Mitochondrial capture (introgression) transfers an entire intact haplogroup, resulting in clean, bifurcating gene trees without reticulate signals. Thus, single-locus mtDNA data cannot mathematically distinguish ILS from historical mitochondrial capture. The paraphyletic patterns observed in widespread species may therefore reflect either ILS or mitochondrial capture—or a combination of both. Instead, under the multispecies coalescent, the sorting time of ancestral polymorphism is proportional to effective population size; during rapid radiations, large populations retain ancestral polymorphisms that are stochastically allocated to descendant lineages, producing a stable signal of ILS [72]. Notably, genome-wide data have detected clear reticulate evolution in *Triplophysa* [13]. Our mitochondrial results provide a complementary perspective: as a single-locus marker, mtDNA is more prone to deviate from the species tree under ILS, yet cannot fully record nuclear gene flow. Therefore, the paraphyly of these five widespread species may reflect ILS during rapid radiation, potentially driven by QTP uplift and climatic oscillations, although historical introgression or mitochondrial capture cannot be ruled out.

*Triplophysa robusta* displays an intermediate pattern: it is divided into two clades in the *COI* tree, one of which clusters with *T. hsutschouensis*, *T. siluroides*, and *T. minxianensis*, whereas it remains monophyletic in the *cytb* tree (Figure 3). Its current distribution forms an east–west belt from the Qinling–Taihang Mountains to the northeastern QTP margin (Figure 4), with core habitats straddling the Loess Plateau–QTP transition zone [23]. This distributional pattern produced a two-center refugial configuration during the LIG (central Loess Plateau and northeastern QTP margin; Figure 5d), which likely facilitated historical contact and mitochondrial gene exchange with Loess Plateau species such as *T. hsutschouensis*, resulting in the *COI* paraphyly [66]. However, the discordance between the *COI* and *cytb* trees for *T. robusta* is unlikely to reflect a gene-specific biological process, because both markers belong to the same non-recombining mitochondrial genome. More plausible explanations include differences in sampling composition, incomplete lineage sorting, or insufficient phylogenetic resolution. Nuclear markers and expanded sampling are needed to determine the cause of this incongruence.

**Figure 8:**
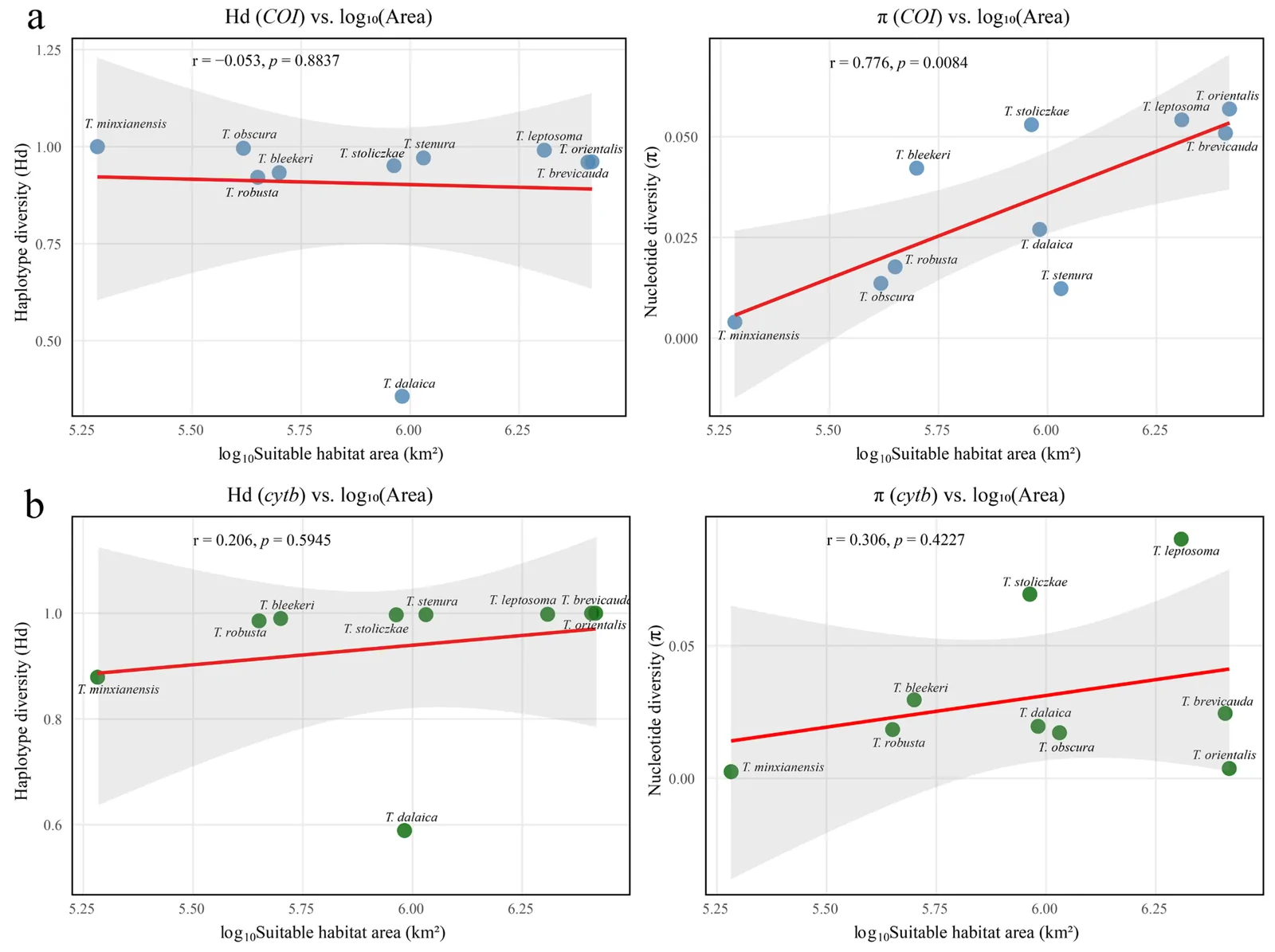
Correlation between nucleotide diversity (π) and suitable habitat area for 10 *Triplophysa* species, excluding two outlier species (*T. siluroides* and *T. hsutschouensis*). (**a**) *COI* π vs. area; (**b**) *cytb* π vs. area. Pearson correlation coefficients (r) and associated *p*-values are shown for each plot.

Genetic diversity was partially associated with range size and demographic history, with the relationship varying among markers and species subsets. The significant positive association was detected primarily for *COI* nucleotide diversity when the two putatively bottleneck-affected species (*T. siluroides* and *T. hsutschouensis*) were excluded, and we explicitly note that the relationship was not significant for *cytb* π or for all 12 species combined. Consistent with this partial association, species with larger suitable habitats tended to harbour higher *COI* nucleotide diversity, although the trend was marker-dependent (Figure 8), consistent with the central-marginal hypothesis, which posits that large, well-connected populations retain greater genetic variation owing to larger effective population sizes and stronger gene flow [73,74,75,76]. The COI π–range correlation was significant only after excluding *T. siluroides* and *T. hsutschouensis*, and its attenuation when all species were included is consistent with the expectation that bottlenecks or founder effects weaken the diversity–range association. Although uneven sampling could influence diversity estimates, we found no significant sample size effect on genetic diversity (*p* > 0.07,), indicating that differential sampling intensity is unlikely to explain the observed diversity–range relationships. This discrepancy—despite both markers residing on the same non-recombining mitochondrial genome—may reflect inherent differences in substitution rate and selective regime between the two genes. The significant *COI* correlation is therefore interpreted as a gene-specific signal and is not generalized to the entire mitochondrial genome. The most widely distributed species—*T. leptosoma*, *T. stoliczkae*, and *T. brevicauda*—exhibited the highest nucleotide and haplotype diversities (Table 1), corresponding to their largest core suitable areas (Figure 4b,e,h). These species underwent pronounced population expansions during the LGM, with the greatest increases in effective population size (Figure 7), and maintained large effective population sizes during interglacials, mirroring the extensive, contiguous habitats identified by species distribution models. In contrast, species with low genetic diversity (*T. siluroides*, *T. hsutschouensis*, *T. minxianensis*) possess significantly smaller and more fragmented current suitable habitats [26]; their core habitats are restricted to local watersheds of the upper Yellow River, with secondary habitats only marginally expanding along watercourses (Figure 4d,f,j). Such restricted ranges reduce effective population sizes and intensify genetic drift, leading to the ongoing loss of nucleotide diversity [77,78,79]. The distinctive pattern of *T. dalaica*—low Hd with moderate π—matches its distribution in isolated plateau lakes, where limited gene flow among lakes depresses haplotype diversity, while prolonged isolation permits moderate nucleotide divergence to accumulate [70].

Effective population size changes reconstructed by BSPs are temporally synchronized with habitat shifts revealed by distribution models, showing contrasting dynamics between expanding and stable species. Species that experienced substantial habitat expansion during the LGM (e.g., *T. leptosoma*, *T. robusta*, *T. stenura*, *T. hsutschouensis*) also underwent synchronous rapid increases in effective population size (Figure 5d,f, Figure 7b and Figure S4), consistent with previous findings [23,26,27]. In particular, *T. stoliczkae* remained stable before ~1.0 Ma, underwent a bottleneck at ~0.12 Ma, and then expanded rapidly (Figure 4a), matching the model-inferred expansion toward the northeastern QTP margin and the eastern Hexi Corridor during the LGM (Figure 5f). Meanwhile, *T. minxianensis* and *T. siluroides* maintained relatively stable effective population size [26] and showed no marked range expansion signals in paleodistribution models (Figure S4a and Figure 5b). *T. obscura* even exhibited a unique pattern in which its distributions during the LIG, LGM, and MHO were all smaller than its current range, consistent with its extremely low genetic diversity and narrow niche (Table 1, Figure 5c).

The two persistent refugia identified by species distribution models—the central Loess Plateau–northern Qinling foothills and the eastern QTP margin–northern Hengduan Mountains—maintained suitable habitats across multiple glacial–interglacial cycles [8,23]. The species with the highest genetic diversity (e.g., *T. leptosoma*, *T. stoliczkae*) had their LIG suitable habitats concentrated in the eastern QTP margin and northern Hengduan Mountains, suggesting that these refugia not only ensured species persistence but also acted as genetic reservoirs, providing source populations for postglacial recolonization [27]. The central Loess Plateau–northern Qinling foothills provided scattered refugial patches for *T. bleekeri*, *T. minxianensis*, *T. robusta*, and *T. stoliczkae* during the LIG; after the LGM, these species expanded toward the main Loess Plateau and western North China (Figure 5b,d,f), a timing broadly matching the rapid effective population size increase in *T. robusta* at ~0.12–0.01 Ma (Figure S4a). This refugium-expansion dynamic demonstrates that long-term stable habitats not only sustain species persistence but also promote genetic diversity accumulation by maintaining large long-term effective population sizes [80,81].

These coupled patterns further imply that under current global warming, species with narrow distributions and low genetic diversity face elevated extinction risk, and that the identified persistent refugia should be prioritized for conservation action.

### 4.3. Conservation Implications for Triplophysa

Our integrative analysis identifies several priority targets for *Triplophysa* conservation. First, two climatically stable refugia—the central Loess Plateau–northern Qinling foothills and the eastern QTP margin–northern Hengduan Mountains—should be designated as priority conservation areas [26]. These regions sustained suitable habitats throughout glacial–interglacial cycles and likely harbor relict populations with unique genetic lineages; protecting these areas is essential for preserving the evolutionary potential of *Triplophysa* under future climate change.

Second, species with severely depleted genetic diversity require urgent intervention. *Triplophysa siluroides* (π < 0.005 in both genes; already listed as a second-class nationally protected animal in China [16], *T. hsutschouensis* (π < 0.007), and *T. dalaica* (the only species with Hd < 0.6) are particularly vulnerable. Such low genetic diversity erodes adaptive capacity and elevates extinction risk [33,34,82]. For these species, we recommend: (1) establishing species-specific monitoring programs to track census and effective population sizes as well as genetic parameters; (2) assessing habitat quality and identifying key threats; and (3) when wild populations continue to decline, implementing ex situ conservation strategies such as captive breeding and assisted gene flow to restore genetic variation and reduce inbreeding depression.

Third, the discordant signals observed in *T. minxianensis* and *T. orientalis*—where *COI* and *cytb* exhibit contrasting diversity patterns—highlight the need for additional nuclear markers to accurately assess their genetic status. Without robust estimates of genome-wide diversity, the conservation prioritization of these species remains uncertain, potentially misdirecting limited resources.

Fourth, the strong positive correlation between habitat suitability and genetic diversity indicates that habitat protection alone may be insufficient; maintaining landscape connectivity to sustain gene flow is equally critical [82]. Species distribution models project further contraction of suitable habitats under future warming, especially for narrow-range endemics [28]. Conservation planning must therefore integrate climate-change projections to identify future refugia, potential migration corridors, and opportunities for genetic rescue.

Finally, the widespread paraphyly and polyphyly observed in multiple species, together with evidence of historical mitochondrial capture [13], suggest that some *Triplophysa* lineages require taxonomic revision. Accurate species delimitation is fundamental to effective conservation, as misclassification can lead to inappropriate management strategies that fail to protect evolutionarily distinct units [66,83,84]. Integrative taxonomic approaches combining molecular, morphological, and ecological data are urgently needed to resolve these uncertainties and define conservation units.

Despite limitations—including reliance on a single-locus mitochondrial marker that may mask nuclear genomic complexity and mitochondrial capture, the equilibrium assumption of MaxEnt models, and limited sample sizes for some species, sample size and spatial autocorrelation limitations. Some species had <20 occurrence records, which may cause overfitting and inflated AUC. Spatial thinning (spThin, ~10 km) reduced but did not eliminate spatial autocorrelation. Future work should use spatial block cross-validation and Moran’s I tests. Together with the equilibrium assumption of MaxEnt, these limitations mean that LGM projections should be treated as provisional. Furthermore, our distribution models also relied exclusively on terrestrial bioclimatic variables (WorldClim bio1–bio19), which may not fully capture the hydrological conditions required by stream-dwelling fishes. Riverine taxa respond primarily to instream flow, water temperature, stream order, and basin topology rather than atmospheric variables alone, and this limitation may partly explain the counter-intuitive LGM habitat expansion. This study represents a significant step toward integrating molecular phylogenetics, population genetics, and species distribution modeling for *Triplophysa* conservation. Future work should focus on: (1) incorporating nuclear markers to validate phylogenetic and population genetic inferences; (2) projecting species distribution models under 2050 and 2070 climate scenarios; (3) assessing the adaptive potential of genetically depauperate populations through functional genomics; (4) incorporating stream-specific hydrological variables (e.g., HydroRIVERS) into future distribution models; and (5) establishing long-term monitoring programs to track population dynamics and genetic viability. Integrating these approaches is essential for developing science-based conservation strategies for *Triplophysa* in a rapidly warming world.

## 5. Conclusions

This study integrates mitochondrial phylogenomics, population genetics, and ecological niche modeling to investigate 12 *Triplophysa* species spanning a continuum of range sizes. Phylogenetic analyses based on mitochondrial gene trees suggest that the genus is monophyletic, with a clear split between cave-dwelling and non-cave-dwelling lineages, although these inferences should be interpreted with caution as they may not fully reflect species relationships. These analyses also reveal a consistent pattern: narrowly distributed endemics form reciprocally monophyletic clades, whereas widespread species are often paraphyletic or polyphyletic, a result consistent with incomplete lineage sorting in large populations. A positive association between *COI* nucleotide diversity and range size was detected after excluding two bottleneck-affected species, though this pattern was not consistent across all markers or when all species were included. Species distribution models identify two climatically stable refugia that likely served as genetic reservoirs for postglacial expansions. We identify three *Triplophysa* species as priority conservation targets and recommend the two refugia as priority protection areas. This study provides an integrative framework linking evolutionary history, genetic diversity, and range dynamics to inform evidence-based conservation of freshwater fishes in biodiversity hotspots facing rapid climate change.

## Figures and Tables

**Table 1:** Genetic diversity parameters of the 12 *Triplophysa* species.

Species	Gene	N	nH	Hd	π	S	Tajima’s *D*	Fu’s *F*s
*T. bleekeri*	*COI*	51	22	0.933	0.0422	101	0.3758	3.7433
	*cytb*	25	23	0.990	0.0296	135	−0.2623	−2.9046
*T. brevicauda*	*COI*	84	59	0.96	0.0509	122	0.9927	−8.7353 *
	*cytb*	70	69	1.000	0.0245	135	−0.2144	−24.0554 ***
*T. dalaica*	*COI*	46	10	0.357	0.0270	55	1.0034	11.4623
	*cytb*	22	6	0.589	0.0196	127	−1.5480 *	14.9710
*T. hsutschouensis*	*COI*	16	8	0.933	0.0059	11	0.2868	−0.9524
	*cytb*	83	37	0.892	0.0064	48	−0.8810	−13.4109 **
*T. leptosoma*	*COI*	27	24	0.991	0.0542	83	1.8319	−3.2394
	*cytb*	36	35	0.998	0.0901	218	2.6860	−4.1312
*T. minxianensis*	*COI*	21	21	1.000	0.0040	23	−2.3369 **	−26.2079 ***
	*cytb*	44	18	0.879	0.0025	30	−2.1111 **	−7.2944 **
*T. obscura*	*COI*	23	22	0.996	0.0136	29	0.1156	−6.4104 *
	*cytb*	—	—	—	—	—	—	—
*T. orientalis*	*COI*	31	20	0.961	0.0569	104	1.6602	2.7200
	*cytb*	13	13	1.000	0.0037	22	−1.8018 *	−9.3285 ***
*T. robusta*	*COI*	195	68	0.921	0.0177	70	−0.3408	−23.0945 ***
	*cytb*	235	122	0.9856	0.0184	153	−0.5437	−23.6904 **
*T. siluroides*	*COI*	30	11	0.894	0.0041	11	−0.3673	−3.2027 *
	*cytb*	29	12	0.874	0.0049	25	−0.7805	−0.9128
*T. stenura*	*COI*	130	52	0.971	0.0123	56	−0.7229	−18.6827 ***
	*cytb*	263	173	0.9973	0.0172	175	−0.9992	−23.6834 **
*T. stoliczkae*	*COI*	92	48	0.951	0.0530	94	2.2824	−1.6205
	*cytb*	57	53	0.997	0.0694	259	1.4192	−0.3211

N, the number of sequences; nH, the number of Haplotypes; Hd, the Haplotype diversity; π, Nucleotide diversity; S, Variable sites. * means *p* < 0.05, ** means *p* < 0.01, *** means *p* < 0.001.

**Table 2:** Analysis of molecular variance (AMOVA) for 12 *Triplophysa* species based on *COI* and *cytb* genes.

	Source of Variation	d.f.	Sum of Squares	Variance Components	Percentage of Variation
*COI*	Among species	11	19,375.653	30.08619 Va	73.96
	Within species	734	7776.372	10.59451 Vb	26.04
	Total	745	27,152.025	40.6807	100.00
*cytb*	Among species	10	75,933.719	106.47031 Va	88.65
	Within species	866	11,806.31	13.63315 Vb	11.35
	Total	876	87,740.029	120.10346	100.00

Note: Va = variance among species; Vb = variance within species. All variance components were statistically significant (*p* < 0.001), This analysis treats each species as a single group and thus partitions genetic variation at the interspecific level.

**Table 3:** Summary of occurrence records, retained bioclimatic variables, and mean AUC values for MaxEnt modeling of the 12 *Triplophysa* species.

Species	Locus	Bioclimate	Average AUC Value
*T. bleekeri*	25	bio3, bio4, bio5, bio14, bio15, bio18	0.960
*T. brevicauda*	17	bio2, bio3, bio9, bio10, bio14, bio15	0.878
*T. dalaica*	43	bio2, bio3, bio5, bio7, bio15, bio17	0.959
*T. hsutschouensis*	20	bio2, bio3, bio6, bio7, bio14, bio15, bio19	0.976
*T. leptosoma*	18	bio2, bio3, bio6, bio7, bio14, bio15, bio19	0.910
*T. minxianensis*	31	bio3, bio4, bio14, bio15, bio17, bio18, bio19	0.990
*T. obscura*	24	bio3, bio5, bio7, bio13, bio15	0.986
*T. orientalis*	27	bio2, bio7, bio9, bio14, bio15	0.893
*T. robusta*	65	bio3, bio5, bio7, bio15, bio18	0.975
*T. siluroides*	21	bio2, bio8, bio9, bio14, bio19	0.922
*T. stenura*	45	bio2, bio3, bio5, bio9, bio18	0.941
*T. stoliczkae*	43	bio3, bio7, bio9, bio15, bio17, bio18	0.948

## Data Availability

The data are contained within the article, and all genes can be obtained by contacting the author (gqh@hunnu.edu.cn).

## Supplementary Materials

The following supporting information can be downloaded at: https://www.mdpi.com/article/10.3390/biology15181667/s1, Additional File S1: DNA extraction, PCR amplification, cloning and sequencing of the mitochondrial genome [85,86] of *Triplophysa erythraea*. Table S1: Mitochondrial genome sequences and taxonomic information of taxa used for phylogenetic reconstruction. Table S2: Summary of *COI* and *cytb* gene sequences downloaded from GenBank for 12 *Triplophysa* species, comprising 746 *COI* and 877 *cytb* sequences used for molecular phylogenetic and genetic diversity analyses. Table S3: Geographic occurrence records (longitude and latitude) of 12 *Triplophysa* species used for MaxEnt modeling. Table S4: The optimal nucleotide substitution model is supported by nine genes, with AICc, and BIC values calculated accordingly. Table S5: Pairwise genetic differentiation (*F*_ST_) among 12 *Triplophysa* species based on *COI* (lower triangle) and *cytb* (upper triangle) sequences. Table S6: Mean pairwise genetic distances (K2P) among 12 *Triplophysa* species based on *COI* gene. Intraspecific distances (mean ± SE) are shown on the diagonal (bold), and interspecific distances (mean ± SE) are shown below the diagonal. SE means the Standard Error. Figure S1: Maximum likelihood (ML) phylogenetic trees of *Triplophysa* inferred from (a) 15 mitochondrial genes and (b) 13 protein-coding genes (PCGs). Bootstrap support values are indicated at the branches. Figure S2: TCS haplotype network of 11 *Triplophysa* species based on *cytb* sequences. Circle size reflects haplotype frequency; colors denote species. The network shows species-specific haplogroups with no shared haplotypes. Figure S3: Phylogenetic relationships and genetic clustering among 12 *Triplophysa* species based on mitochondrial *COI* and *cytb* genes. (a) ML tree constructed from *COI* sequences; (b) ML tree from *cytb* sequences; Bootstrap support values are indicated at the branches. Figure S4: Bayesian Skyline Plots (BSP) of effective population size (*N*_e_) trajectories for 12 *Triplophysa* species based on *COI* and *cytb* sequences. (a) Species with concordant signals between the two markers; (b) Species showing minor marker discrepancies (*T. hsutschouensis*, *T. dalaica*, and *T. orientalis*), and *T. obscura* (only *COI* data). Time is shown in millions of years ago (Ma); 0 denotes the present. Solid lines and shaded areas represent median estimates and 95% HPD intervals, respectively. Figure S5: Jackknife tests of variable importance (AUC on test data) for MaxEnt models of 12 *Triplophysa* species. The plots show the variables with the highest predictive power when used alone and the variables whose omission caused the greatest reduction in model performance, identifying temperature- and precipitation-related variables as the primary drivers of species distribution. a, *T. bleekeri*; b, *T*. *brevicauda*; c, *T. dalaica*; d, *T. hsutschouensis*; e, *T. leptosoma*; f, *T. minxianensisi*; g, *T. obscura*; h, *T. orientalis*; i, *T. robusta*; j, *T. siluroides*; k, *T. stenura*; l, *T. stoliczkae*. Figure S6: Occurrence localities of the 12 *Triplophysa* species used for MaxEnt modeling, mapped onto a digital elevation model of China. Colors denote species as shown in the legend. The distribution points are derived from GBIF and published literature, and correspond to the five ecogeographic groups identified in the current distribution models (Figure 4).
