# Supplementary material for: Molecular Systematics and Distribution Pattern Evolution of Triplophysa Species in China: A Comparison of Widespread and Narrow-Range Taxa
Source: Biology (Basel). 2026 Sep 20;15(18):1667. doi: 10.3390/biology15181667 (PMC13604416; doi:10.3390/biology15181667)
Supplement: Supplementary file 1 [file biology-15-01667-s001.zip › Additional File S1.pdf]

Additional file S1

Molecular Systematics and Distribution Pattern Evolution of *Triplophysa* Species in China: A Comparison of Widespread and Narrow-Range Taxa

Yixuan Chen[1,†], Minyi Wang[1,†], Ziyi Wang[1,†], Xiao Chen[1], Shengnan Li[1,], Conghui Yang[1,], Dengqiang Wang[2], Xinbin Duan[2,3], Huiwu Tian[2,3,*], Qianhong Gu[1,*]

DNA extraction, PCR amplification, cloning and sequencing of the mitochondrial genome of Triplophysa erythraea

Total genomic DNA was isolated from peripheral blood cells of *Triplophysa erythraea* using standard phenol–chloroform extraction procedures [85]. The complete mitochondrial genome was amplified in 22 contiguous and overlapping fragments using a set of highly conserved, newly designed primers (You et al., 2014). PCR amplifications were performed in 50 μL reactions containing 10–30 ng of template DNA, 1.5 mM $MgCl_2$, 250 μM dNTPs, 0.4 μM of each primer, and 1.25 U of Taq polymerase (TaKaRa, Dalian, China). The thermal cycling profile consisted of an initial denaturation at 94°C for 5 min, followed by 30 cycles of denaturation at 94°C for 35 s, annealing at 50–60°C for 60 s, and extension at 72°C for 60–150 s, with a final extension at 72°C for 10 min. Most amplification products were sequenced directly; fragments that could not be sequenced directly were cloned into the pMD18-T vector (TaKaRa, Dalian, China) and transformed into *Escherichia coli* DH5*α*. Plasmid DNA was purified, and at least three clones per amplicon were sequenced bidirectionally with vector-specific primers using a primer-walking strategy on an ABI 3730XL automated sequencer (Applied Biosystems, CA, USA). Sequence assembly, editing, and homology assessment were carried out using BioEdit [86] and the DNAstar 5.0 software package (DNAstar Inc.).

References

85. Sambrook, J.; Russell, D.W. *Molecular Cloning: A Laboratory Manual*; Cold Spring Harbor Laboratory Press: New York, NY, USA, 1989; Volume 3.
86. Hall, T.A. BioEdit: A user-friendly biological sequence alignment editor and analysis program for Windows 95/98/NT. In *Nucleic Acids Symposium Series*; Information: London, UK, 1999.
