# Supplementary material for: Molecular Systematics and Distribution Pattern Evolution of Triplophysa Species in China: A Comparison of Widespread and Narrow-Range Taxa
Source: Biology (Basel). 2026 Sep 20;15(18):1667. doi: 10.3390/biology15181667 (PMC13604416; doi:10.3390/biology15181667)
Supplement: Supplementary file 1 [file biology-15-01667-s001.zip › Fig. S2.pdf]

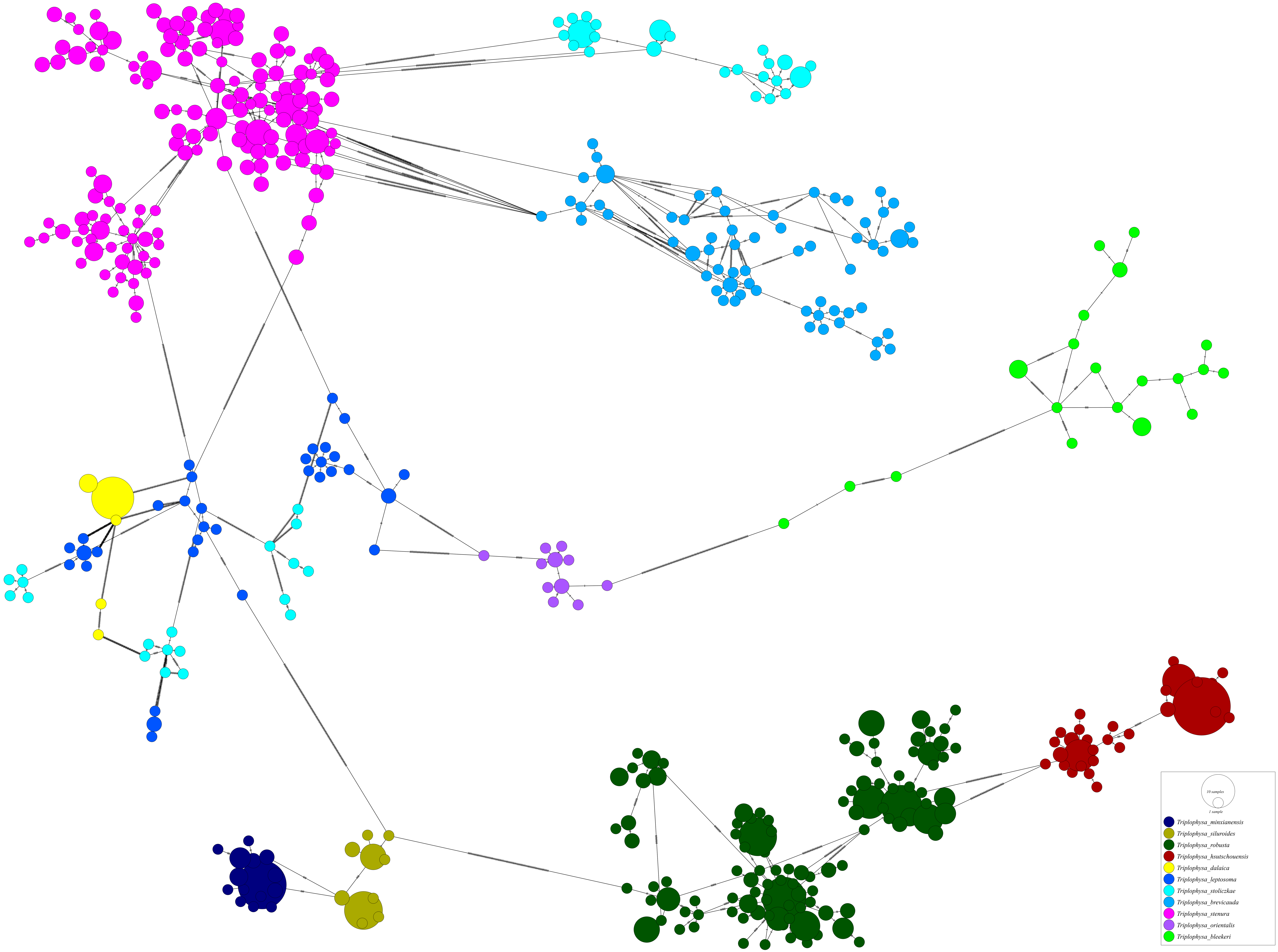
10 samples
1 sample
Triplophysa_minxianensis
Triplophysa_siluroides
Triplophysa_robusta
Triplophysa_hsutschouensis
Triplophysa_dalaica
Triplophysa_leptosoma
Triplophysa_stoliczkae
Triplophysa_brevicauda
Triplophysa_stenura
Triplophysa_orientalis
Triplophysa_bleekeri
