# Supplementary material for: Molecular Systematics and Distribution Pattern Evolution of Triplophysa Species in China: A Comparison of Widespread and Narrow-Range Taxa
Source: Biology (Basel). 2026 Sep 20;15(18):1667. doi: 10.3390/biology15181667 (PMC13604416; doi:10.3390/biology15181667)
Supplement: Supplementary file 1 [file biology-15-01667-s001.zip › Fig. S3.pdf]

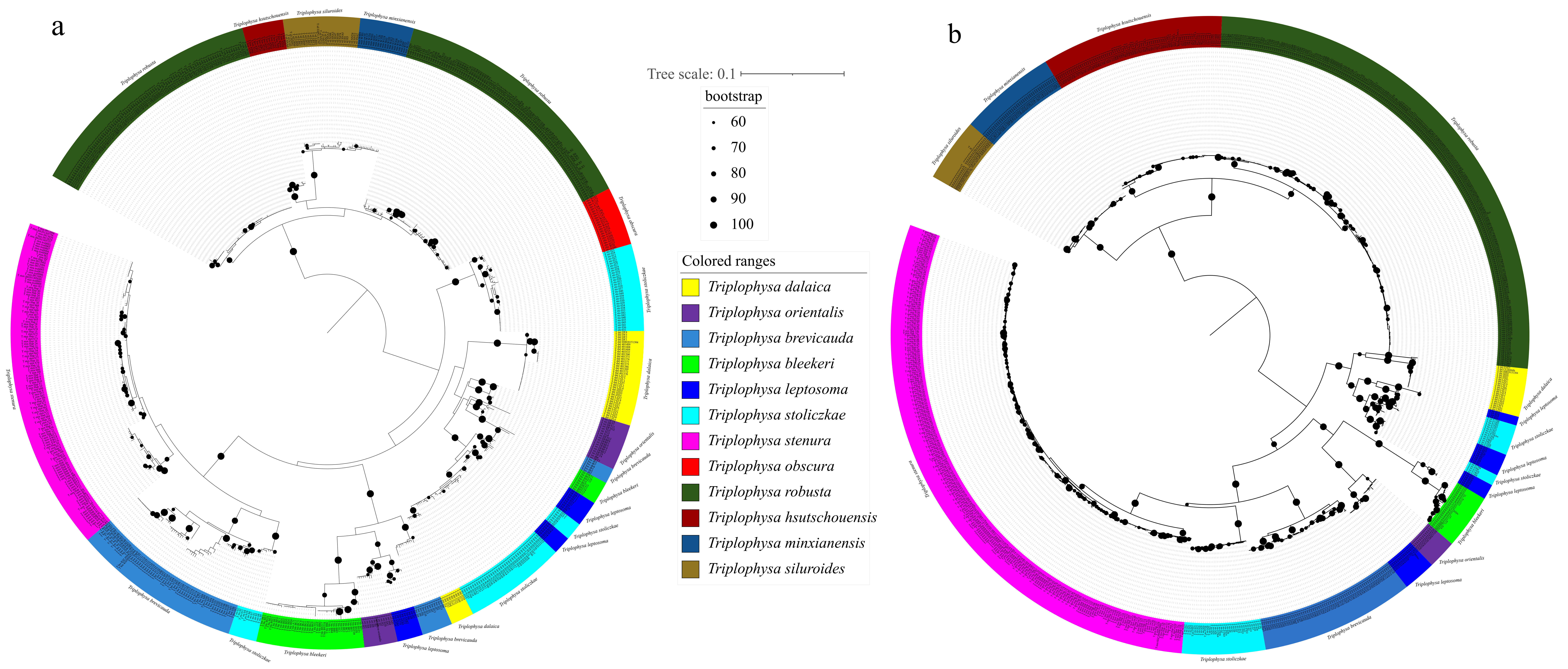
a
b
Tree scale: 0.1
bootstrap
60
70
80
90
100
Colored ranges
Triplophysa dalaica
Triplophysa orientalis
Triplophysa brevicauda
Triplophysa bleekeri
Triplophysa leptosoma
Triplophysa stoliczkae
Triplophysa stenura
Triplophysa obscura
Triplophysa robusta
Triplophysa hsutschouensis
Triplophysa minxianensis
Triplophysa siluroides
