# Supplementary material for: Molecular Systematics and Distribution Pattern Evolution of Triplophysa Species in China: A Comparison of Widespread and Narrow-Range Taxa
Source: Biology (Basel). 2026 Sep 20;15(18):1667. doi: 10.3390/biology15181667 (PMC13604416; doi:10.3390/biology15181667)
Supplement: Supplementary file 1 [file biology-15-01667-s001.zip › Fig. S4a.pdf]

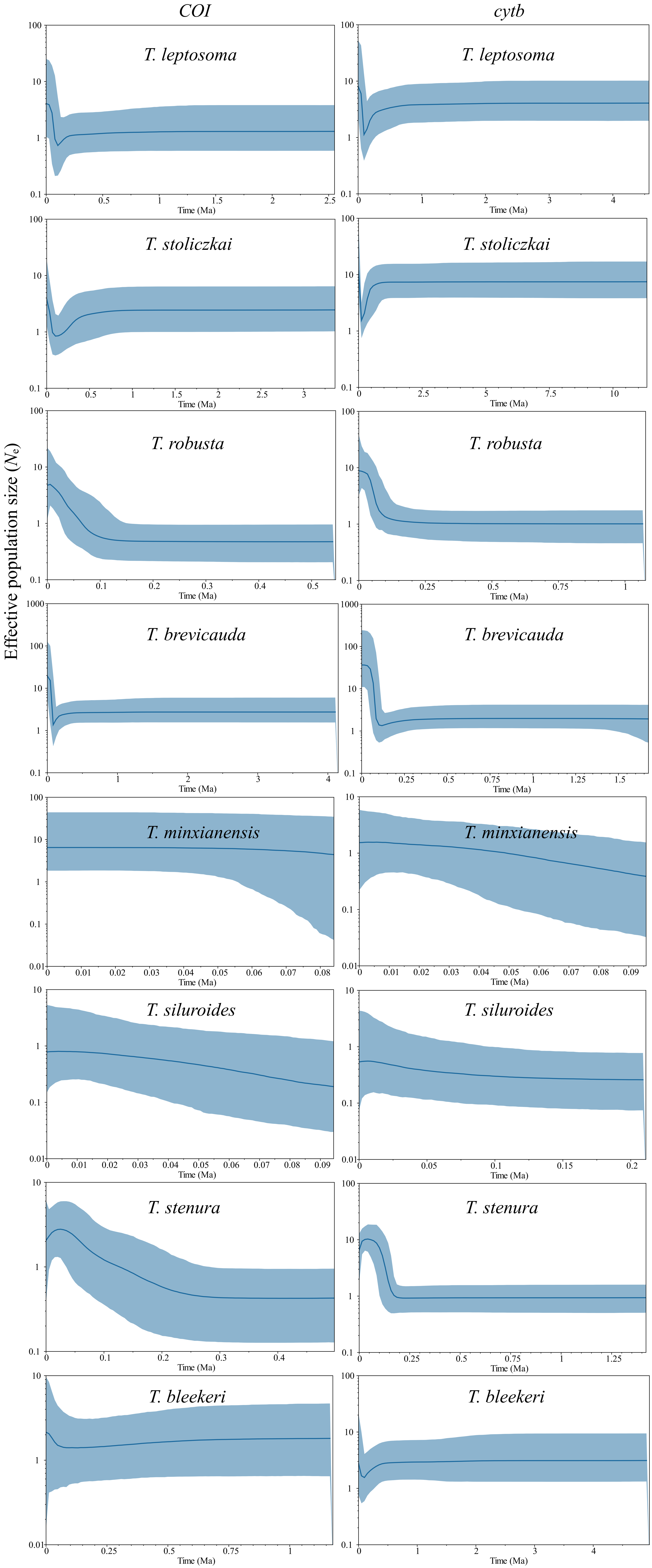

COI
cytb
Effective population size (N_e)
T. leptosoma
T. stoliczkai
T. robusta
T. brevicauda
T. minxianensis
T. siluroides
T. stenura
T. bleekeri
Time (Ma)
