# Supplementary material for: Molecular Systematics and Distribution Pattern Evolution of Triplophysa Species in China: A Comparison of Widespread and Narrow-Range Taxa
Source: Biology (Basel). 2026 Sep 20;15(18):1667. doi: 10.3390/biology15181667 (PMC13604416; doi:10.3390/biology15181667)
Supplement: Supplementary file 1 [file biology-15-01667-s001.zip › Table S1-6.pdf]

Table S1. Mitochondrial genome sequences and taxonomic information of taxa used for phylogenetic reconstruction.

Specimen ID	GenBank	Species name	Genus	Family
Phenacogrammus inerruptus	AB054129	*Phenacogrammus inerruptus*	*Phenacogrammus*	Alestidae
Homaloptera parclitella	AP011438	*Homaloptera parclitella*	*Homaloptera*	Balitoridae
Jinshaia abbreviata1	KJ754936	*Jinshaia abbreviata*	*Jinshaia*	Balitoridae
Jinshaia abbreviata2	AP013302	*Jinshaia abbreviata*	*Jinshaia*	Balitoridae
Jinshaia sinensis1	KJ739867	*Jinshaia sinensis*	*Jinshaia*	Balitoridae
Jinshaia sinensis2	JX155733	*Jinshaia sinensis*	*Jinshaia*	Balitoridae
Lepturichthys fimbriata1	MN882155	*Lepturichthys fimbriata*	*Lepturichthys*	Balitoridae
Lepturichthys fimbriata2	KJ830772	*Lepturichthys fimbriata*	*Lepturichthys*	Balitoridae
Pseudogastromyzon fasciatus	KX101229	*Pseudogastromyzon fasciatus*	*Pseudogastromyzon*	Balitoridae
Sewellia lineolata	AP011292	*Sewellia lineolata*	*Sewellia*	Balitoridae
Sinogastromyzon puliensis1	AP011298	*Sinogastromyzon puliensis*	*Sinogastromyzon*	Balitoridae
Sinogastromyzon puliensis2	FJ605359	*Sinogastromyzon puliensis*	*Sinogastromyzon*	Balitoridae
Sinogastromyzon szechuanensis1	MW006667	*Sinogastromyzon szechuanensis*	*Sinogastromyzon*	Balitoridae
Sinogastromyzon szechuanensis2	KY352772	*Sinogastromyzon szechuanensis*	*Sinogastromyzon*	Balitoridae
Botia dario	MG751071	*Botia dario*	*Botia*	Botiidae
Botia lohachata	KP729183	*Botia lohachata*	*Botia*	Botiidae
Leptobotia elongata1	KY307845	*Leptobotia elongata*	*Leptobotia*	Botiidae
Leptobotia elongata2	JQ230103	*Leptobotia elongata*	*Leptobotia*	Botiidae
Leptobotia microphthalma1	KY307846	*Leptobotia microphthalma*	*Leptobotia*	Botiidae
Leptobotia microphthalma2	KC865424	*Leptobotia microphthalma*	*Leptobotia*	Botiidae
Leptobotia pellegrini	AP011350	*Leptobotia pellegrini*	*Leptobotia*	Botiidae
Leptobotia rubrilabris1	KY307847	*Leptobotia rubrilabris*	*Leptobotia*	Botiidae
Leptobotia rubrilabris2	KF534784	*Leptobotia rubrilabris*	*Leptobotia*	Botiidae

Specimen ID	GenBank	Species name	Genus	Family
Leptobotia taeniops1	AP013304	*Leptobotia taeniops*	*Leptobotia*	Botiidae
Leptobotia taeniops2	KM386686	*Leptobotia taeniops*	*Leptobotia*	Botiidae
Parabotia lijiangensis1	MT850133	*Parabotia lijiangensis*	*Parabotia*	Botiidae
Parabotia lijiangensis2	MT323118	*Parabotia lijiangensis*	*Parabotia*	Botiidae
Sinibotia pulchra1	AP012125	*Sinibotia pulchra*	*Sinibotia*	Botiidae
Sinibotia pulchra2	KT362179	*Sinibotia pulchra*	*Sinibotia*	Botiidae
Sinibotia robusta	KT374012	*Sinibotia robusta*	*Sinibotia*	Botiidae
Sinibotia superciliaris1	MN709617	*Sinibotia superciliaris*	*Sinibotia*	Botiidae
Sinibotia superciliaris2	KY307848	*Sinibotia superciliaris*	*Sinibotia*	Botiidae
Carpiodes carpio1	AY366087	*Carpiodes carpio*	*Carpiodes*	Catostomidae
Carpiodes carpio2	AB126083	*Carpiodes carpio*	*Carpiodes*	Catostomidae
Carpiodes cyprinus1	MW856859	*Carpiodes cyprinus*	*Carpiodes*	Catostomidae
Carpiodes cyprinus2	MW856858	*Carpiodes cyprinus*	*Carpiodes*	Catostomidae
Catostomus catostomus1	MG570442	*Catostomus catostomus*	*Catostomus*	Catostomidae
Catostomus catostomus2	MG570441	*Catostomus catostomus*	*Catostomus*	Catostomidae
Catostomus commersonii1	MH324426	*Catostomus commersonii*	*Catostomus*	Catostomidae
Catostomus commersonii2	MH324427	*Catostomus commersonii*	*Catostomus*	Catostomidae
Cycleptus elongatus	AB126082	*Cycleptus elongatus*	*Cycleptus*	Catostomidae
Deltistes luxatus1	MT501375	*Deltistes luxatus*	*Deltistes*	Catostomidae
Deltistes luxatus2	MT501374	*Deltistes luxatus*	*Deltistes*	Catostomidae
Erimyzon oblongus1	MG570411	*Erimyzon oblongus*	*Erimyzon*	Catostomidae
Erimyzon oblongus2	MG570410	*Erimyzon oblongus*	*Erimyzon*	Catostomidae
Ictiobus cyprinellus	KP306894	*Ictiobus cyprinellus*	*Ictiobus*	Catostomidae
Minytrema melanops1	MG570430	*Minytrema melanops*	*Minytrema*	Catostomidae
Minytrema melanops2	MG570436	*Minytrema melanops*	*Minytrema*	Catostomidae

**Specimen ID**	**GenBank**	**Species name**	**Genus**	**Family**
Moxostoma anisurum1	MW856894	*Moxostoma anisurum*	*Moxostoma*	Catostomidae
Moxostoma anisurum2	MW856880	*Moxostoma anisurum*	*Moxostoma*	Catostomidae
Myxocyprinus asiaticus1	AY986503	*Myxocyprinus asiaticus*	*Myxocyprinus*	Catostomidae
Myxocyprinus asiaticus2	AY526869	*Myxocyprinus asiaticus*	*Myxocyprinus*	Catostomidae
Xyrauchen texanus1	MT501364	*Xyrauchen texanus*	*Xyrauchen*	Catostomidae
Xyrauchen texanus2	MT501363	*Xyrauchen texanus*	*Xyrauchen*	Catostomidae
Cobitis choii1	EU333980	*Cobitis choii*	*Cobitis*	Cobitidae
Cobitis choii2	EU656112	*Cobitis choii*	*Cobitis*	Cobitidae
Cobitis hankugensis1	MZ339224	*Cobitis hankugensis*	*Cobitis*	Cobitidae
Cobitis hankugensis2	MN841275	*Cobitis hankugensis*	*Cobitis*	Cobitidae
Cobitis macrostigma1	MK156771	*Cobitis macrostigma*	*Cobitis*	Cobitidae
Cobitis macrostigma2	MT259034	*Cobitis macrostigma*	*Cobitis*	Cobitidae
Cobitis striata1	AP010782	*Cobitis striata*	*Cobitis*	Cobitidae
Cobitis striata2	AB054125	*Cobitis striata*	*Cobitis*	Cobitidae
Cobitis takatsuensis1	AP011290	*Cobitis takatsuensis*	*Cobitis*	Cobitidae
Cobitis takatsuensis2	AP009306	*Cobitis takatsuensis*	*Cobitis*	Cobitidae
Iksookimia koreensis1	AP013308	*Iksookimia koreensis*	*Iksookimia*	Cobitidae
Iksookimia koreensis2	KT952321	*Iksookimia koreensis*	*Iksookimia*	Cobitidae
Iksookimia pacifica1	OM312055	*Iksookimia pacifica*	*Iksookimia*	Cobitidae
Iksookimia pacifica2	OK053203	*Iksookimia pacifica*	*Iksookimia*	Cobitidae
Kichulchoia multifasciata1	KR075135	*Kichulchoia multifasciata*	*Kichulchoia*	Cobitidae
Kichulchoia multifasciata2	AP011337	*Kichulchoia multifasciata*	*Kichulchoia*	Cobitidae
Koreocobitis naktongensis1	JN607252	*Koreocobitis naktongensis*	*Koreocobitis*	Cobitidae
Koreocobitis naktongensis2	HM535625	*Koreocobitis naktongensis*	*Koreocobitis*	Cobitidae
Koreocobitis rotundicaudata1	JN607253	*Koreocobitis rotundicaudata*	*Koreocobitis*	Cobitidae

Specimen ID	GenBank	Species name	Genus	Family
Koreocobitis rotundicaudata2	AP011339	*Koreocobitis rotundicaudata*	*Koreocobitis*	Cobitidae
Lefua costata	KT943751	*Lefua costata*	*Lefua*	Cobitidae
Lefua nikkonis	AP011300	*Lefua nikkonis*	*Lefua*	Cobitidae
Misgurnus anguillicaudatus1	DQ026434	*Misgurnus anguillicaudatus*	*Misgurnus*	Cobitidae
Misgurnus anguillicaudatus2	MF579257	*Misgurnus anguillicaudatus*	*Misgurnus*	Cobitidae
Misgurnus anguillicaudatus3	MT896815	*Misgurnus anguillicaudatus*	*Misgurnus*	Cobitidae
Misgurnus mizolepis1	MF579258	*Misgurnus mizolepis*	*Misgurnus*	Cobitidae
Misgurnus mizolepis2	MW304001	*Misgurnus mizolepis*	*Misgurnus*	Cobitidae
Misgurnus mohoity	KF386025	*Misgurnus mohoity*	*Misgurnus*	Cobitidae
Niwaella delicata1	AP011230	*Niwaella delicata*	*Niwaella*	Cobitidae
Niwaella delicata2	AP009308	*Niwaella delicata*	*Niwaella*	Cobitidae
Pangio oblonga	AP011336	*Pangio oblonga*	*Pangio*	Cobitidae
Paramisgurnus dabryanus1	KR349175	*Paramisgurnus dabryanus*	*Paramisgurnus*	Cobitidae
Paramisgurnus dabryanus2	KF771003	*Paramisgurnus dabryanus*	*Paramisgurnus*	Cobitidae
Protocobitis anteroventris	PQ591769	*Protocobitis anteroventris*	*Protocobitis*	Cobitidae
Protocobitis typhlops	OR713096	*Protocobitis typhlops*	*Protocobitis*	Cobitidae
Canthophrys gongota	AP011289	*Canthophrys gongota*	*Canthophrys*	Cobitidae
Ellopostoma mystax	AP011447	*Ellopostoma mystax*	*Ellopostoma*	Ellopostomatidae
Gonorynchus greyi	AB054134	*Gonorynchus greyi*	*Gonorynchus*	Gonorynchidae
Gyrinocheilus aymonieri1	DQ026432	*Gyrinocheilus aymonieri*	*Gyrinocheilus*	Gyrinocheilidae
Gyrinocheilus aymonieri2	AB242164	*Gyrinocheilus aymonieri*	*Gyrinocheilus*	Gyrinocheilidae
Gyrinocheilus pennocki	AP011226	*Gyrinocheilus pennocki*	*Gyrinocheilus*	Gyrinocheilidae
Acanthocobitis botia1	AP012139	*Acanthocobitis botia*	*Acanthocobitis*	Nemacheilidae
Acanthocobitis botia2	AP012138	*Acanthocobitis botia*	*Acanthocobitis*	Nemacheilidae
Barbatula barbatula	KP715096	*Barbatula barbatula*	*Barbatula*	Nemacheilidae

Specimen ID	GenBank	Species name	Genus	Family
Barbatula toni1	MK900633	*Barbatula toni*	*Barbatula*	Nemacheilidae
Barbatula toni2	AB242162	*Barbatula toni*	*Barbatula*	Nemacheilidae
Eonemachilus longidorsalis	OM732331	*Eonemachilus longidorsalis*	*Eonemachilus*	Nemacheilidae
Hedinichthys yarkandensis1	KP050360	*Heminoemacheilus parvus*	*Heminoemacheilus*	Nemacheilidae
Hedinichthys yarkandensis2	KT192439	*Heminoemacheilus parvus*	*Heminoemacheilus*	Nemacheilidae
Hedinichthys yarkandensis3	KT224367	*Heminoemacheilus parvus*	*Heminoemacheilus*	Nemacheilidae
Hedinichthys yarkandensis4	KX447641	*Heminoemacheilus parvus*	*Heminoemacheilus*	Nemacheilidae
Hedinichthys yarkandensis5	MN821008	*Heminoemacheilus parvus*	*Heminoemacheilus*	Nemacheilidae
Hedinichthys yarkandensis6	OP554813	*Heminoemacheilus parvus*	*Heminoemacheilus*	Nemacheilidae
Heminoemacheilus zhengbaoshani1	ON116530	*Heminoemacheilus zhengbaoshani*	*Heminoemacheilus*	Nemacheilidae
Heminoemacheilus zhengbaoshani2	OQ024379	*Heminoemacheilus zhengbaoshani*	*Heminoemacheilus*	Nemacheilidae
Heminoemacheilus parvus	ON116520	*Heminoemacheilus parvus*	*Heminoemacheilus*	Nemacheilidae
Homatula potanini1	KP749475	*Homatula potanini*	*Homatula*	Nemacheilidae
Homatula potanini2	KM017732	*Homatula potanini*	*Homatula*	Nemacheilidae
Homatula variegata	JX144893	*Homatula variegata*	*Homatula*	Nemacheilidae
Micronemacheilus pulcherrimus1	ON116493	*Micronemacheilus pulcherrimus*	*Micronemacheilus*	Nemacheilidae
Micronemacheilus pulcherrimus2	OQ024374	*Micronemacheilus pulcherrimus*	*Micronemacheilus*	Nemacheilidae
Nemachilichthys rueppelli	AP011305	*Nemachilichthys rueppelli*	*Nemachilichthys*	Nemacheilidae
Oreonectes anophthalmus	ON116506	*Oreonectes anophthalmus*	*Oreonectes*	Nemacheilidae
Oreonectes guananensis	ON116507	*Oreonectes guananensis*	*Oreonectes*	Nemacheilidae
Oreonectes luochengensis	ON116495	*Oreonectes luochengensis*	*Oreonectes*	Nemacheilidae
Oreonectes platycephalus	ON116528	*Oreonectes platycephalus*	*Oreonectes*	Nemacheilidae
Oreonectes polystigmus1	ON116517	*Oreonectes polystigmus*	*Oreonectes*	Nemacheilidae
Oreonectes polystigmus2	ON116514	*Oreonectes polystigmus*	*Oreonectes*	Nemacheilidae
Paranemachilus genilepis1	ON116497	*Paranemachilus genilepis*	*Paranemachilus*	Nemacheilidae

Specimen ID	GenBank	Species name	Genus	Family
Paranemachilus genilepis2	MW532082	*Paranemachilus genilepis*	*Paranemachilus*	Nemacheilidae
Paranemachilus genilepis3	MT845213	*Paranemachilus genilepis*	*Paranemachilus*	Nemacheilidae
Paranemachilus pingguoensis	ON116500	*Paranemachilus pingguoensis*	*Paranemachilus*	Nemacheilidae
Paranemachilus sp. r LD-2023	OQ024378	*Paranemachilus* sp.	*Paranemachilus*	Nemacheilidae
Schistura fasciolata1	MW192448	*Schistura fasciolata*	*Schistura*	Nemacheilidae
Schistura fasciolata2	MZ853159	*Schistura fasciolata*	*Schistura*	Nemacheilidae
Schistura notostigma	AP011308	*Schistura notostigma*	*Schistura*	Nemacheilidae
Traccatichthys pulcher	ON116516	*Traccatichthys pulcher*	*Traccatichthys*	Nemacheilidae
Traccatichthys taeniatus	KF765806	*Traccatichthys taeniatus*	*Traccatichthys*	Nemacheilidae
Traccatichthys zispi	ON116518	*Traccatichthys zispi*	*Traccatichthys*	Nemacheilidae
Triplophysa aliensis	KT213584	*Triplophysa aliensis*	*Triplophysa*	Nemacheilidae
Triplophysa alticeps	KT213585	*Triplophysa alticeps*	*Triplophysa*	Nemacheilidae
Triplophysa angeli	MZ325251	*Triplophysa angeli*	*Triplophysa*	Nemacheilidae
Triplophysa anterodorsalis	KJ739868	*Triplophysa anterodorsalis*	*Triplophysa*	Nemacheilidae
Triplophysa baotianensis	MT992550	*Triplophysa baotianensis*	*Triplophysa*	Nemacheilidae
Triplophysa bleekeri	JX135578	*Triplophysa bleekeri*	*Triplophysa*	Nemacheilidae
Triplophysa bombifrons1	MZ958819	*Triplophysa bombifrons*	*Triplophysa*	Nemacheilidae
Triplophysa bombifrons2	OP499856	*Triplophysa bombifrons*	*Triplophysa*	Nemacheilidae
Triplophysa brevibarba	KY971608	*Triplophysa brevibarba*	*Triplophysa*	Nemacheilidae
Triplophysa brevicauda	KT213588	*Triplophysa brevicauda*	*Triplophysa*	Nemacheilidae
Triplophysa chondrostoma	KT213589	*Triplophysa chondrostoma*	*Triplophysa*	Nemacheilidae
Triplophysa cuneicephala	KY945352	*Triplophysa cuneicephala*	*Triplophysa*	Nemacheilidae
Triplophysa dalaica1	KT213590	*Triplophysa dalaica*	*Triplophysa*	Nemacheilidae
Triplophysa dalaica2	KY945353	*Triplophysa dalaica*	*Triplophysa*	Nemacheilidae
Triplophysa dalaica3	OR857523	*Triplophysa dalaica*	*Triplophysa*	Nemacheilidae

Specimen ID	GenBank	Species name	Genus	Family
Triplophysa dalaica4	OR857524	*Triplophysa dalaica*	*Triplophysa*	Nemacheilidae
Triplophysa dalaica5	OR857525	*Triplophysa dalaica*	*Triplophysa*	Nemacheilidae
Triplophysa dalaica6	OR857526	*Triplophysa dalaica*	*Triplophysa*	Nemacheilidae
Triplophysa dalaica7	OR857527	*Triplophysa dalaica*	*Triplophysa*	Nemacheilidae
Triplophysa dorsalis1	KT213591	*Triplophysa dorsalis*	*Triplophysa*	Nemacheilidae
Triplophysa dorsalis2	KT241024	*Triplophysa dorsalis*	*Triplophysa*	Nemacheilidae
Triplophysa erythraea1	PP455386	*Triplophysa erythraea*	*Triplophysa*	Nemacheilidae
Triplophysa erythraea2	PQ040451	*Triplophysa erythraea*	*Triplophysa*	Nemacheilidae
Triplophysa erythraea3	HMGYQ	*Triplophysa erythraea*	*Triplophysa*	Nemacheilidae
Triplophysa fengshanensis	OQ998929	*Triplophysa fengshanensis*	*Triplophysa*	Nemacheilidae
Triplophysa grahami	PP114297	*Triplophysa grahami*	*Triplophysa*	Nemacheilidae
Triplophysa hsutschouensis1	KT213592	*Triplophysa hsutschouensis*	*Triplophysa*	Nemacheilidae
Triplophysa hsutschouensis2	OR730670	*Triplophysa hsutschouensis*	*Triplophysa*	Nemacheilidae
Triplophysa huapingensis	OQ998930	*Triplophysa huapingensis*	*Triplophysa*	Nemacheilidae
Triplophysa jianchuanensis	OQ603602	*Triplophysa jianchuanensis*	*Triplophysa*	Nemacheilidae
Triplophysa labiata1	KT192057	*Triplophysa labiata*	*Triplophysa*	Nemacheilidae
Triplophysa labiata2	OQ559481	*Triplophysa labiata*	*Triplophysa*	Nemacheilidae
Triplophysa langpingensis	ON116521	*Triplophysa langpingensis*	*Triplophysa*	Nemacheilidae
Triplophysa leptosoma	KT213593	*Triplophysa leptosoma*	*Triplophysa*	Nemacheilidae
Triplophysa lixianensis	KT966735	*Triplophysa lixianensis*	*Triplophysa*	Nemacheilidae
Triplophysa longibarbatus	MT361977	*Triplophysa longibarbatus*	*Triplophysa*	Nemacheilidae
Triplophysa longipectoralis	OQ998928	*Triplophysa longipectoralis*	*Triplophysa*	Nemacheilidae
Triplophysa longliensis	OQ998931	*Triplophysa longliensis*	*Triplophysa*	Nemacheilidae
Triplophysa macrocephala	PP204900	*Triplophysa macrocephala*	*Triplophysa*	Nemacheilidae
Triplophysa markehenensis	KT213594	*Triplophysa markehenensis*	*Triplophysa*	Nemacheilidae

Specimen ID	GenBank	Species name	Genus	Family
Triplophysa microphthalma	PP979136	*Triplophysa microphthalma*	*Triplophysa*	Nemacheilidae
Triplophysa microps	KT213595	*Triplophysa microps*	*Triplophysa*	Nemacheilidae
Triplophysa minxianensis	KT213596	*Triplophysa minxianensis*	*Triplophysa*	Nemacheilidae
Triplophysa moquensis	KT213597	*Triplophysa moquensis*	*Triplophysa*	Nemacheilidae
Triplophysa nandanensis	OQ998932	*Triplophysa nandanensis*	*Triplophysa*	Nemacheilidae
Triplophysa nanpanjiangensis	OQ274895	*Triplophysa nanpanjiangensis*	*Triplophysa*	Nemacheilidae
Triplophysa nasobarbatula1	MT361978	*Triplophysa nasobarbatula*	*Triplophysa*	Nemacheilidae
Triplophysa nasobarbatula2	ON116529	*Triplophysa nasobarbatula*	*Triplophysa*	Nemacheilidae
Triplophysa nasobarbatula3	OQ992507	*Triplophysa nasobarbatula*	*Triplophysa*	Nemacheilidae
Triplophysa nujiangensa	KT213598	*Triplophysa nujiangensa*	*Triplophysa*	Nemacheilidae
Triplophysa orientalis1	KJ631323	*Triplophysa orientalis*	*Triplophysa*	Nemacheilidae
Triplophysa orientalis2	KT213599	*Triplophysa orientalis*	*Triplophysa*	Nemacheilidae
Triplophysa pappenheimi1	KT213600	*Triplophysa pappenheimi*	*Triplophysa*	Nemacheilidae
Triplophysa pappenheimi2	KY419201	*Triplophysa pappenheimi*	*Triplophysa*	Nemacheilidae
Triplophysa pseudoscleroptera	KU587513	*Triplophysa pseudoscleroptera*	*Triplophysa*	Nemacheilidae
Triplophysa pseudostenura	KT213601	*Triplophysa pseudostenura*	*Triplophysa*	Nemacheilidae
Triplophysa qiubeiensis	PQ563459	*Triplophysa qiubeiensis*	*Triplophysa*	Nemacheilidae
Triplophysa robusta1	KM396312	*Triplophysa robusta*	*Triplophysa*	Nemacheilidae
Triplophysa robusta2	KM406486	*Triplophysa robusta*	*Triplophysa*	Nemacheilidae
Triplophysa rosa	JF268621	*Triplophysa rosa*	*Triplophysa*	Nemacheilidae
Triplophysa scleroptera	KT213602	*Triplophysa scleroptera*	*Triplophysa*	Nemacheilidae
Triplophysa sellaefer	KY851112	*Triplophysa sellaefer*	*Triplophysa*	Nemacheilidae
Triplophysa siluroides1	KJ781206	*Triplophysa siluroides*	*Triplophysa*	Nemacheilidae
Triplophysa siluroides2	KT213603	*Triplophysa siluroides*	*Triplophysa*	Nemacheilidae
Triplophysa sp.1	KT213586	*Triplophysa* sp.	*Triplophysa*	Nemacheilidae

**Specimen ID**	**GenBank**	**Species name**	**Genus**	**Family**
Triplophysa sp.1.TL	ON116519	*Triplophysa* sp.	*Triplophysa*	Nemacheilidae
Triplophysa sp.2	KT213587	*Triplophysa* sp.	*Triplophysa*	Nemacheilidae
Triplophysa sp.3	KT259195	*Triplophysa* sp.	*Triplophysa*	Nemacheilidae
Triplophysa sp.TL	KT456271	*Triplophysa* sp.	*Triplophysa*	Nemacheilidae
Triplophysa sp.y	PQ356185	*Triplophysa* sp.	*Triplophysa*	Nemacheilidae
Triplophysa sp.ZP1	KX376478	*Triplophysa* sp.	*Triplophysa*	Nemacheilidae
Triplophysa sp.ZP2	KX376479	*Triplophysa* sp.	*Triplophysa*	Nemacheilidae
Triplophysa stenura1	KT213604	*Triplophysa stenura*	*Triplophysa*	Nemacheilidae
Triplophysa stenura2	KX354975	*Triplophysa stenura*	*Triplophysa*	Nemacheilidae
Triplophysa stewarti1	KJ631324	*Triplophysa stewarti*	*Triplophysa*	Nemacheilidae
Triplophysa stewarti2	KT213605	*Triplophysa stewarti*	*Triplophysa*	Nemacheilidae
Triplophysa stoliczkai	JQ663847	*Triplophysa stoliczkai*	*Triplophysa*	Nemacheilidae
Triplophysa strauchii1	KP297875	*Triplophysa strauchii*	*Triplophysa*	Nemacheilidae
Triplophysa strauchii2	KP979754	*Triplophysa strauchii*	*Triplophysa*	Nemacheilidae
Triplophysa tenuis1	KT224363	*Triplophysa tenuis*	*Triplophysa*	Nemacheilidae
Triplophysa tenuis2	OR730671	*Triplophysa tenuis*	*Triplophysa*	Nemacheilidae
Triplophysa tianeensis	OQ998933	*Triplophysa tianeensis*	*Triplophysa*	Nemacheilidae
Triplophysa tibetana1	KM212178	*Triplophysa tibetana*	*Triplophysa*	Nemacheilidae
Triplophysa tibetana2	KT122845	*Triplophysa tibetana*	*Triplophysa*	Nemacheilidae
Triplophysa tibetana3	KT224364	*Triplophysa tibetana*	*Triplophysa*	Nemacheilidae
Triplophysa ulacholica	KT259194	*Triplophysa ulacholica*	*Triplophysa*	Nemacheilidae
Triplophysa venusta	KT008666	*Triplophysa venusta*	*Triplophysa*	Nemacheilidae
Triplophysa weiheensis	PP203140	*Triplophysa weiheensis*	*Triplophysa*	Nemacheilidae
Triplophysa wulongensis	PP025975	*Triplophysa wulongensis*	*Triplophysa*	Nemacheilidae
Triplophysa wuweiensis	KT224365	*Triplophysa wuweiensis*	*Triplophysa*	Nemacheilidae

**Specimen ID**	**GenBank**	**Species name**	**Genus**	**Family**
Triplophysa xiangxiensis	KT751089	*Triplophysa xiangxiensis*	*Triplophysa*	Nemacheilidae
Triplophysa xichangensis	KT224366	*Triplophysa xichangensis*	*Triplophysa*	Nemacheilidae
Triplophysa zhenfengensis1	MT992551	*Triplophysa zhenfengensis*	*Triplophysa*	Nemacheilidae
Triplophysa zhenfengensis2	OQ998934	*Triplophysa zhenfengensis*	*Triplophysa*	Nemacheilidae
Troglonectes acridorsalis	ON116515	*Troglonectes acridorsalis*	*Troglonectes*	Nemacheilidae
Troglonectes barbatus	NC_073130	*Troglonectes barbatus*	*Troglonectes*	Nemacheilidae
Troglonectes daqikongensis	ON116526	*Troglonectes daqikongensis*	*Troglonectes*	Nemacheilidae
Troglonectes donglanensis	ON116505	*Troglonectes donglanensis*	*Troglonectes*	Nemacheilidae
Troglonectes duanensis	ON116509	*Troglonectes duanensis*	*Troglonectes*	Nemacheilidae
Troglonectes elongatus	ON116502	*Troglonectes elongatus*	*Troglonectes*	Nemacheilidae
Troglonectes furcocaudalis1	ON116512	*Troglonectes furcocaudalis*	*Troglonectes*	Nemacheilidae
Troglonectes furcocaudalis2	ON116532	*Troglonectes furcocaudalis*	*Troglonectes*	Nemacheilidae
Troglonectes jiarongensis1	ON116523	*Troglonectes jiarongensis*	*Troglonectes*	Nemacheilidae
Troglonectes jiarongensis2	ON116524	*Troglonectes jiarongensis*	*Troglonectes*	Nemacheilidae
Troglonectes jiarongensis3	ON116527	*Troglonectes jiarongensis*	*Troglonectes*	Nemacheilidae
Troglonectes lihuensis	ON148332	*Troglonectes lihuensis*	*Troglonectes*	Nemacheilidae
Troglonectes macrolepis	ON116498	*Troglonectes macrolepis*	*Troglonectes*	Nemacheilidae
Troglonectes microphthalmus	ON116494	*Troglonectes microphthalmus*	*Troglonectes*	Nemacheilidae
Troglonectes shuilongensis	ON116522	*Troglonectes shuilongensis*	*Troglonectes*	Nemacheilidae
Troglonectes sp.2	ON116525	*Troglonectes* sp.	*Troglonectes*	Nemacheilidae
Troglonectes translucens	ON116510	*Troglonectes translucens*	*Troglonectes*	Nemacheilidae
Yunnanilus bailianensis	ON116504	*Yunnanilus bailianensis*	*Yunnanilus*	Nemacheilidae
Yunnanilus cruciatus	AP012142	*Yunnanilus cruciatus*	*Yunnanilus*	Nemacheilidae
Yunnanilus jiuchiensis	MW532080	*Yunnanilus jiuchiensis*	*Yunnanilus*	Nemacheilidae
Yunnanilus longibarbatus	ON116508	*Yunnanilus longibarbatus*	*Yunnanilus*	Nemacheilidae

Specimen ID	GenBank	Species name	Genus	Family
Yunnanilus nanpanjiangensis	PP079025	*Yunnanilus nanpanjiangensis*	*Yunnanilus*	Nemacheilidae
Yunnanilus niger	MW553077	*Yunnanilus niger*	*Yunnanilus*	Nemacheilidae
Yunnanilus pleurotaenia1	NC_063105	*Yunnanilus pleurotaenia*	*Yunnanilus*	Nemacheilidae
Yunnanilus pleurotaenia2	OQ024381	*Yunnanilus pleurotaenia*	*Yunnanilus*	Nemacheilidae
Yunnanilus pulcherrimus	MK387705	*Yunnanilus pulcherrimus*	*Yunnanilus*	Nemacheilidae
Yunnanilus retrodorsalis	NC_073137	*Yunnanilus retrodorsalis*	*Yunnanilus*	Nemacheilidae
Vaillantella maassi	AB242173	*Vaillantella maassi*	*Vaillantella*	Vaillantellidae

Table S2. Summary of *COI* and *cytb* gene sequences downloaded from GenBank for 12 *Triplophysa* species, comprising 746 *COI* and 877 *cytb* sequences used for molecular phylogenetic and genetic diversity analyses.

Species	GenBank *cytb*	Species	GenBank *COI*
Barbatula minxianensis	MG697523	*Barbatula minxianensis*	MT271396
Barbatula minxianensis	MG697522	*Barbatula minxianensis*	MT271395
Barbatula minxianensis	MG697520	*Barbatula minxianensis*	MT271394
Barbatula minxianensis	MG697519	*Barbatula minxianensis*	MT271393
Barbatula minxianensis	MG697518	*Barbatula minxianensis*	MT271392
Barbatula minxianensis	MG697517	*Barbatula minxianensis*	MT271391
Barbatula minxianensis	MG697516	*Barbatula minxianensis*	MT271390
Barbatula minxianensis	MG697514	*Barbatula minxianensis*	MT271389
Barbatula minxianensis	MG697513	*Barbatula minxianensis*	MT271388
Barbatula minxianensis	MG697511	*Barbatula minxianensis*	MT271387
Barbatula minxianensis	MG697510	*Barbatula minxianensis*	MT271386
Barbatula minxianensis	MG697509	*Barbatula minxianensis*	MT271385
Barbatula minxianensis	MG697508	*Barbatula minxianensis*	MT271384
Barbatula minxianensis	MG697507	*Barbatula minxianensis*	MT271383
Barbatula minxianensis	MG697505	*Barbatula minxianensis*	MT271382
Barbatula minxianensis	MG697506	*Barbatula minxianensis*	MT271380
Barbatula minxianensis	MG697504	*Barbatula minxianensis*	MT271379
Barbatula minxianensis	MG697503	*Barbatula minxianensis*	MT271378
Barbatula minxianensis	MG697502	*Barbatula minxianensis*	MT271377
Barbatula minxianensis	MG697501	*Barbatula minxianensis*	MT271376
Barbatula minxianensis	MG697500	*Barbatula minxianensis*	MT271374
Barbatula minxianensis	MG697499	*Triplophysa bleekeri*	MT572021
Barbatula minxianensis	MG697498	*Triplophysa bleekeri*	MT264969
Barbatula minxianensis	MG697497	*Triplophysa bleekeri*	MF123337
Barbatula minxianensis	MG697496	*Triplophysa bleekeri*	MF123335
Barbatula minxianensis	MG697495	*Triplophysa bleekeri*	MF123334
Barbatula minxianensis	MG697494	*Triplophysa bleekeri*	MF123333
Barbatula minxianensis	MG697493	*Triplophysa bleekeri*	KU557942
Barbatula minxianensis	MG697492	*Triplophysa bleekeri*	KU557941
Barbatula minxianensis	MG697491	*Triplophysa bleekeri*	KU557940
Barbatula minxianensis	MG697490	*Triplophysa bleekeri*	KU557939
Barbatula minxianensis	MG697489	*Triplophysa bleekeri*	KU557938
Barbatula minxianensis	MG697488	*Triplophysa bleekeri*	KU557937
Barbatula minxianensis	MG697487	*Triplophysa bleekeri*	KU557936
Barbatula minxianensis	MG697486	*Triplophysa bleekeri*	KU557935
Barbatula minxianensis	MG697485	*Triplophysa bleekeri*	KU557934
Barbatula minxianensis	MG697484	*Triplophysa bleekeri*	KU557933
Barbatula minxianensis	MG697483	*Triplophysa bleekeri*	KU557932
Barbatula minxianensis	MG697482	*Triplophysa bleekeri*	KU557931

**Species**	**GenBank *cytb***	**Species**	**GenBank *COI***
Barbatula minxianensis	MG697481	*Triplophysa bleekeri*	KU557930
Barbatula minxianensis	MG697480	*Triplophysa bleekeri*	KU557929
Barbatula minxianensis	MG697479	*Triplophysa bleekeri*	KU557928
Barbatula minxianensis	MG697478	*Triplophysa bleekeri*	KU557927
Barbatula minxianensis	MG697477	*Triplophysa bleekeri*	KU557926
Triplophysa bleekeri	FJ406605	*Triplophysa bleekeri*	KU557925
Triplophysa bleekeri	FJ406604	*Triplophysa bleekeri*	KU557924
Triplophysa bleekeri	FJ406603	*Triplophysa bleekeri*	KU557923
Triplophysa bleekeri	FJ406602	*Triplophysa bleekeri*	KU557922
Triplophysa bleekeri	FJ406601	*Triplophysa bleekeri*	KU557921
Triplophysa bleekeri	FJ406600	*Triplophysa bleekeri*	KU557920
Triplophysa bleekeri	FJ406599	*Triplophysa bleekeri*	KU557919
Triplophysa bleekeri	FJ406598	*Triplophysa bleekeri*	KU557918
Triplophysa bleekeri	FJ406597	*Triplophysa bleekeri*	KU557917
Triplophysa bleekeri	FJ406596	*Triplophysa bleekeri*	KU557916
Triplophysa bleekeri	FJ406595	*Triplophysa bleekeri*	KU557915
Triplophysa bleekeri	FJ406594	*Triplophysa bleekeri*	KU557914
Triplophysa bleekeri	FJ406593	*Triplophysa bleekeri*	KU557913
Triplophysa bleekeri	FJ406592	*Triplophysa bleekeri*	KU557912
Triplophysa bleekeri	FJ406591	*Triplophysa bleekeri*	KU557911
Triplophysa bleekeri	FJ406590	*Triplophysa bleekeri*	KU557910
Triplophysa bleekeri	FJ406589	*Triplophysa bleekeri*	KU557909
Triplophysa bleekeri	FJ406588	*Triplophysa bleekeri*	KU557908
Triplophysa bleekeri	FJ406587	*Triplophysa bleekeri*	KU557907
Triplophysa bleekeri	FJ406581	*Triplophysa bleekeri*	KU557906
Triplophysa bleekeri	FJ406580	*Triplophysa bleekeri*	KU557905
Triplophysa bleekeri	KX373847	*Triplophysa bleekeri*	KU557904
Triplophysa bleekeri	KX394258	*Triplophysa bleekeri*	KU557903
Triplophysa bleekeri	KX394257	*Triplophysa bleekeri*	KU557902
Triplophysa bleekeri	KX394256	*Triplophysa bleekeri*	KU557901
Triplophysa brevicauda	MK655354	*Triplophysa bleekeri*	KU557900
Triplophysa brevicauda	MK655353	*Triplophysa bleekeri*	KU557899
Triplophysa brevicauda	MK655352	*Triplophysa bleekeri*	KU557898
Triplophysa brevicauda	MK655351	*Triplophysa brevicauda*	MG238206
Triplophysa brevicauda	MK655350	*Triplophysa brevicauda*	MZ677092
Triplophysa brevicauda	MK655349	*Triplophysa brevicauda*	MK655045
Triplophysa brevicauda	MK655348	*Triplophysa brevicauda*	MK655044
Triplophysa brevicauda	MK655347	*Triplophysa brevicauda*	MK655043
Triplophysa brevicauda	MK655346	*Triplophysa brevicauda*	MK655042
Triplophysa brevicauda	MK655345	*Triplophysa brevicauda*	MK655041
Triplophysa brevicauda	MK655344	*Triplophysa brevicauda*	MK655040
Triplophysa brevicauda	MK655343	*Triplophysa brevicauda*	MK655039
Triplophysa brevicauda	MK655342	*Triplophysa brevicauda*	MK655038

**Species**	**GenBank *cytb***	**Species**	**GenBank *COI***
Triplophysa brevicauda	MK655341	*Triplophysa brevicauda*	MK655037
Triplophysa brevicauda	MK655340	*Triplophysa brevicauda*	MK655036
Triplophysa brevicauda	MK655339	*Triplophysa brevicauda*	MK655035
Triplophysa brevicauda	MK655338	*Triplophysa brevicauda*	MK655034
Triplophysa brevicauda	MK655337	*Triplophysa brevicauda*	MK655033
Triplophysa brevicauda	MK655336	*Triplophysa brevicauda*	MK655032
Triplophysa brevicauda	MK655335	*Triplophysa brevicauda*	MK655031
Triplophysa brevicauda	MK655334	*Triplophysa brevicauda*	MK655030
Triplophysa brevicauda	MK655333	*Triplophysa brevicauda*	MK655029
Triplophysa brevicauda	MK655332	*Triplophysa brevicauda*	MK655028
Triplophysa brevicauda	MK655331	*Triplophysa brevicauda*	MK655027
Triplophysa brevicauda	MK655330	*Triplophysa brevicauda*	MK655026
Triplophysa brevicauda	MK655329	*Triplophysa brevicauda*	MK655025
Triplophysa brevicauda	MK655328	*Triplophysa brevicauda*	MK655024
Triplophysa brevicauda	MK655327	*Triplophysa brevicauda*	MK655023
Triplophysa brevicauda	MK655326	*Triplophysa brevicauda*	MK655022
Triplophysa brevicauda	MK655325	*Triplophysa brevicauda*	MK655021
Triplophysa brevicauda	MK655324	*Triplophysa brevicauda*	MK655020
Triplophysa brevicauda	MK655323	*Triplophysa brevicauda*	MK655019
Triplophysa brevicauda	MK655322	*Triplophysa brevicauda*	MK655018
Triplophysa brevicauda	MK655321	*Triplophysa brevicauda*	MK655017
Triplophysa brevicauda	MK655320	*Triplophysa brevicauda*	MK655016
Triplophysa brevicauda	MK655319	*Triplophysa brevicauda*	MK655015
Triplophysa brevicauda	MK655318	*Triplophysa brevicauda*	MK655014
Triplophysa brevicauda	MK655317	*Triplophysa brevicauda*	MK655013
Triplophysa brevicauda	MK655316	*Triplophysa brevicauda*	MK655012
Triplophysa brevicauda	MK655315	*Triplophysa brevicauda*	MK655011
Triplophysa brevicauda	MK655314	*Triplophysa brevicauda*	MK655010
Triplophysa brevicauda	MK655313	*Triplophysa brevicauda*	MK655009
Triplophysa brevicauda	MK655312	*Triplophysa brevicauda*	MK655008
Triplophysa brevicauda	MK655311	*Triplophysa brevicauda*	MK655007
Triplophysa brevicauda	MK655310	*Triplophysa brevicauda*	MK655006
Triplophysa brevicauda	MK655309	*Triplophysa brevicauda*	MK655005
Triplophysa brevicauda	MK655308	*Triplophysa brevicauda*	MK655004
Triplophysa brevicauda	MK655307	*Triplophysa brevicauda*	MK655003
Triplophysa brevicauda	MK655306	*Triplophysa brevicauda*	MK655002
Triplophysa brevicauda	MK655305	*Triplophysa brevicauda*	MK655001
Triplophysa brevicauda	MK655304	*Triplophysa brevicauda*	MK655000
Triplophysa brevicauda	MK655303	*Triplophysa brevicauda*	MK654999
Triplophysa brevicauda	MK655302	*Triplophysa brevicauda*	MT413242
Triplophysa brevicauda	MK655301	*Triplophysa brevicauda*	MT413241
Triplophysa brevicauda	MK655300	*Triplophysa brevicauda*	MT413240
Triplophysa brevicauda	MK655299	*Triplophysa brevicauda*	MT413239

**Species**	**GenBank *cytb***	**Species**	**GenBank *COI***
Triplophysa brevicauda	MK655298	*Triplophysa brevicauda*	MT413238
Triplophysa brevicauda	MK655297	*Triplophysa brevicauda*	MT413237
Triplophysa brevicauda	MK655296	*Triplophysa brevicauda*	MT413236
Triplophysa brevicauda	MK655295	*Triplophysa brevicauda*	MT413235
Triplophysa brevicauda	MK655293	*Triplophysa brevicauda*	MT413234
Triplophysa brevicauda	MK655292	*Triplophysa brevicauda*	MT413233
Triplophysa brevicauda	MK655291	*Triplophysa brevicauda*	MT413232
Triplophysa brevicauda	MK655290	*Triplophysa brevicauda*	MT413231
Triplophysa brevicauda	MK655289	*Triplophysa brevicauda*	MW086884
Triplophysa brevicauda	MK655288	*Triplophysa brevicauda*	MW086883
Triplophysa brevicauda	MK655287	*Triplophysa brevicauda*	MF123338
Triplophysa brevicauda	MK655286	*Triplophysa brevicauda*	KU557962
Triplophysa brevicauda	MW086888	*Triplophysa brevicauda*	KU557961
Triplophysa brevicauda	MW086887	*Triplophysa brevicauda*	KU557960
Triplophysa dalaica	KY945353	*Triplophysa brevicauda*	KU557959
Triplophysa dalaica	KX373845	*Triplophysa brevicauda*	KU557958
Triplophysa dalaica	MG697586	*Triplophysa brevicauda*	KU557957
Triplophysa dalaica	MN896551	*Triplophysa brevicauda*	KU557956
Triplophysa dalaica	MN896552	*Triplophysa brevicauda*	KU557955
Triplophysa dalaica	MN896553	*Triplophysa brevicauda*	KU557954
Triplophysa dalaica	MN896554	*Triplophysa brevicauda*	KU557953
Triplophysa dalaica	MN896555	*Triplophysa brevicauda*	KU557952
Triplophysa dalaica	MN896556	*Triplophysa brevicauda*	KU557951
Triplophysa dalaica	MN896557	*Triplophysa brevicauda*	KU557950
Triplophysa dalaica	MN896558	*Triplophysa brevicauda*	KU557949
Triplophysa dalaica	MN896559	*Triplophysa brevicauda*	KU557948
Triplophysa dalaica	MN896560	*Triplophysa brevicauda*	KU557947
Triplophysa dalaica	MN896561	*Triplophysa brevicauda*	KU557946
Triplophysa dalaica	MN896562	*Triplophysa brevicauda*	KU557945
Triplophysa dalaica	MN896563	*Triplophysa brevicauda*	KU557944
Triplophysa dalaica	MN896564	*Triplophysa brevicauda*	KU557943
Triplophysa dalaica	MN896565	*Triplophysa dalaica*	KY945353
Triplophysa dalaica	MN896566	*Triplophysa dalaica*	MN896286
Triplophysa dalaica	MN896567	*Triplophysa dalaica*	MN896287
Triplophysa dalaica	MN896568	*Triplophysa dalaica*	MN896288
Triplophysa dalaica	MN896569	*Triplophysa dalaica*	MN896289
Triplophysa hsutschouensis	MG697366	*Triplophysa dalaica*	MN896290
Triplophysa hsutschouensis	MG697365	*Triplophysa dalaica*	MN896291
Triplophysa hsutschouensis	MG697364	*Triplophysa dalaica*	MN896292
Triplophysa hsutschouensis	MG697363	*Triplophysa dalaica*	MN896293
Triplophysa hsutschouensis	MG697362	*Triplophysa dalaica*	MN896294
Triplophysa hsutschouensis	MG697361	*Triplophysa dalaica*	MN896295
Triplophysa hsutschouensis	MG697360	*Triplophysa dalaica*	MN896296

**Species**	**GenBank *cytb***	**Species**	**GenBank *COI***
Triplophysa hsutschouensis	MG697359	*Triplophysa dalaica*	MN896297
Triplophysa hsutschouensis	MG697358	*Triplophysa dalaica*	MN896298
Triplophysa hsutschouensis	MG697357	*Triplophysa dalaica*	MN896299
Triplophysa hsutschouensis	MG697356	*Triplophysa dalaica*	MN896300
Triplophysa hsutschouensis	MG697355	*Triplophysa dalaica*	MN896301
Triplophysa hsutschouensis	MG697354	*Triplophysa dalaica*	MN896302
Triplophysa hsutschouensis	MG697353	*Triplophysa dalaica*	MN896303
Triplophysa hsutschouensis	MG697352	*Triplophysa dalaica*	MN896304
Triplophysa hsutschouensis	MG697351	*Triplophysa dalaica*	MK250412
Triplophysa hsutschouensis	MG697350	*Triplophysa dalaica*	MK250392
Triplophysa hsutschouensis	MG697349	*Triplophysa dalaica*	MK250393
Triplophysa hsutschouensis	MG697348	*Triplophysa dalaica*	MK250394
Triplophysa hsutschouensis	MG697347	*Triplophysa dalaica*	MK250395
Triplophysa hsutschouensis	MG697346	*Triplophysa dalaica*	MK250396
Triplophysa hsutschouensis	MG697345	*Triplophysa dalaica*	MK250397
Triplophysa hsutschouensis	MG697344	*Triplophysa dalaica*	MK250398
Triplophysa hsutschouensis	MG697343	*Triplophysa dalaica*	MK250402
Triplophysa hsutschouensis	MG697342	*Triplophysa dalaica*	MK250405
Triplophysa hsutschouensis	MG697341	*Triplophysa dalaica*	MK250408
Triplophysa hsutschouensis	MG697340	*Triplophysa dalaica*	MK250409
Triplophysa hsutschouensis	MG697339	*Triplophysa dalaica*	MK250410
Triplophysa hsutschouensis	MG697338	*Triplophysa dalaica*	KU557966
Triplophysa hsutschouensis	MG697337	*Triplophysa dalaica*	KU557967
Triplophysa hsutschouensis	MG697336	*Triplophysa dalaica*	KU557968
Triplophysa hsutschouensis	MG697335	*Triplophysa dalaica*	KU557969
Triplophysa hsutschouensis	MG697334	*Triplophysa dalaica*	MT264971
Triplophysa hsutschouensis	MG697333	*Triplophysa dalaica*	MT264972
Triplophysa hsutschouensis	MG697332	*Triplophysa dalaica*	MT264973
Triplophysa hsutschouensis	MG697331	*Triplophysa dalaica*	MT264974
Triplophysa hsutschouensis	MG697330	*Triplophysa dalaica*	MT264975
Triplophysa hsutschouensis	MG697329	*Triplophysa dalaica*	MT264976
Triplophysa hsutschouensis	MG697328	*Triplophysa dalaica*	MT264977
Triplophysa hsutschouensis	MG697327	*Triplophysa dalaica*	MT264978
Triplophysa hsutschouensis	MG697326	*Triplophysa dalaica*	MT264979
Triplophysa hsutschouensis	MG697325	*Triplophysa hsutschouensis*	OQ359317
Triplophysa hsutschouensis	MG697324	*Triplophysa hsutschouensis*	OQ359318
Triplophysa hsutschouensis	MG697323	*Triplophysa hsutschouensis*	OQ359319
Triplophysa hsutschouensis	MG697322	*Triplophysa hsutschouensis*	OQ359320
Triplophysa hsutschouensis	MG697321	*Triplophysa hsutschouensis*	OQ359321
Triplophysa hsutschouensis	MG697320	*Triplophysa hsutschouensis*	OQ359322
Triplophysa hsutschouensis	MG697319	*Triplophysa hsutschouensis*	OQ359323
Triplophysa hsutschouensis	MG697318	*Triplophysa hsutschouensis*	OQ359324
Triplophysa hsutschouensis	MG697317	*Triplophysa hsutschouensis*	MT271370

**Species**	**GenBank *cytb***	**Species**	**GenBank *COI***
Triplophysa hsutschouensis	MG697316	*Triplophysa hsutschouensis*	MT271369
Triplophysa hsutschouensis	MG697315	*Triplophysa hsutschouensis*	MT271368
Triplophysa hsutschouensis	MG697314	*Triplophysa hsutschouensis*	MT271367
Triplophysa hsutschouensis	MG697313	*Triplophysa hsutschouensis*	MT271366
Triplophysa hsutschouensis	MG697312	*Triplophysa hsutschouensis*	MT271365
Triplophysa hsutschouensis	MG697311	*Triplophysa hsutschouensis*	MT271364
Triplophysa hsutschouensis	MG697310	*Triplophysa hsutschouensis*	MT271363
Triplophysa hsutschouensis	MG697309	*Triplophysa leptosoma*	OQ359339
Triplophysa hsutschouensis	MG697308	*Triplophysa leptosoma*	OQ359341
Triplophysa hsutschouensis	MG697307	*Triplophysa leptosoma*	OQ359340
Triplophysa hsutschouensis	MG697306	*Triplophysa leptosoma*	MT413243
Triplophysa hsutschouensis	MG697305	*Triplophysa leptosoma*	MT413244
Triplophysa hsutschouensis	MG697304	*Triplophysa leptosoma*	MT413245
Triplophysa hsutschouensis	MG697303	*Triplophysa leptosoma*	MT413246
Triplophysa hsutschouensis	MG697302	*Triplophysa leptosoma*	KX213673
Triplophysa hsutschouensis	MG697301	*Triplophysa leptosoma*	KX213674
Triplophysa hsutschouensis	MG697300	*Triplophysa leptosoma*	KX213675
Triplophysa hsutschouensis	MG697299	*Triplophysa leptosoma*	KX213678
Triplophysa hsutschouensis	MG697298	*Triplophysa leptosoma*	KX213679
Triplophysa hsutschouensis	MG697297	*Triplophysa leptosoma*	KX213680
Triplophysa hsutschouensis	MG697296	*Triplophysa leptosoma*	KX213681
Triplophysa hsutschouensis	MG697295	*Triplophysa leptosoma*	KX213682
Triplophysa hsutschouensis	MG697294	*Triplophysa leptosoma*	KX213683
Triplophysa hsutschouensis	MG697293	*Triplophysa leptosoma*	KX213684
Triplophysa hsutschouensis	MG697292	*Triplophysa leptosoma*	KX213685
Triplophysa hsutschouensis	MG697291	*Triplophysa leptosoma*	KX213686
Triplophysa hsutschouensis	MG697290	*Triplophysa leptosoma*	KX213687
Triplophysa hsutschouensis	MG697289	*Triplophysa leptosoma*	KX213689
Triplophysa hsutschouensis	MG697288	*Triplophysa leptosoma*	KX213690
Triplophysa hsutschouensis	MG697287	*Triplophysa leptosoma*	KX213691
Triplophysa hsutschouensis	MG697286	*Triplophysa leptosoma*	KX213692
Triplophysa hsutschouensis	MG697285	*Triplophysa leptosoma*	MT271373
Triplophysa hsutschouensis	KX373852	*Triplophysa leptosoma*	MT271372
Triplophysa leptosoma	KX213634	*Triplophysa leptosoma*	MT271371
Triplophysa leptosoma	KX213635	*Triplophysa obscura*	MT271397
Triplophysa leptosoma	KX213636	*Triplophysa obscura*	MT271398
Triplophysa leptosoma	KX213637	*Triplophysa obscura*	MT271402
Triplophysa leptosoma	KX213638	*Triplophysa obscura*	MT271404
Triplophysa leptosoma	KX213639	*Triplophysa obscura*	MT271405
Triplophysa leptosoma	KX213640	*Triplophysa obscura*	MT271406
Triplophysa leptosoma	KX213645	*Triplophysa obscura*	MT271410
Triplophysa leptosoma	KX213646	*Triplophysa obscura*	MT271411
Triplophysa leptosoma	KX213647	*Triplophysa obscura*	MT271412

**Species**	**GenBank *cytb***	**Species**	**GenBank *COI***
Triplophysa leptosoma	KX213648	*Triplophysa obscura*	MT271413
Triplophysa leptosoma	KX213649	*Triplophysa obscura*	MT271414
Triplophysa leptosoma	KX213650	*Triplophysa obscura*	MT271415
Triplophysa leptosoma	KX213651	*Triplophysa obscura*	MT271416
Triplophysa leptosoma	KX213652	*Triplophysa obscura*	MT271417
Triplophysa leptosoma	KX213653	*Triplophysa obscura*	MT271418
Triplophysa leptosoma	KX213654	*Triplophysa obscura*	MT271419
Triplophysa leptosoma	KX213655	*Triplophysa obscura*	MT271420
Triplophysa leptosoma	KX213656	*Triplophysa obscura*	MT271423
Triplophysa leptosoma	KX213657	*Triplophysa obscura*	MT271424
Triplophysa leptosoma	KX213658	*Triplophysa obscura*	MT271426
Triplophysa leptosoma	KX213659	*Triplophysa obscura*	MT271427
Triplophysa leptosoma	KX213660	*Triplophysa obscura*	MT271428
Triplophysa leptosoma	KX213661	*Triplophysa obscura*	MT271429
Triplophysa leptosoma	KX213662	*Triplophysa orientalis*	KU558018
Triplophysa leptosoma	KX213663	*Triplophysa orientalis*	KU558019
Triplophysa leptosoma	KX213664	*Triplophysa orientalis*	KU558020
Triplophysa leptosoma	KX213665	*Triplophysa orientalis*	KU558021
Triplophysa leptosoma	KX213666	*Triplophysa orientalis*	KU558022
Triplophysa leptosoma	KX213667	*Triplophysa orientalis*	KU558023
Triplophysa leptosoma	KX213668	*Triplophysa orientalis*	KU558024
Triplophysa leptosoma	KX213669	*Triplophysa orientalis*	KU558025
Triplophysa leptosoma	KX213670	*Triplophysa orientalis*	KU558027
Triplophysa leptosoma	KX213671	*Triplophysa orientalis*	KU558029
Triplophysa leptosoma	KX213672	*Triplophysa orientalis*	KU558030
Triplophysa leptosoma	KX373839	*Triplophysa orientalis*	KU558031
Triplophysa orientalis	MK655271	*Triplophysa orientalis*	KU558032
Triplophysa orientalis	MK655272	*Triplophysa orientalis*	KU558033
Triplophysa orientalis	MK655273	*Triplophysa orientalis*	KU558034
Triplophysa orientalis	MK655274	*Triplophysa orientalis*	KU558035
Triplophysa orientalis	MK655275	*Triplophysa orientalis*	KU558036
Triplophysa orientalis	MK655276	*Triplophysa orientalis*	KU558037
Triplophysa orientalis	MK655277	*Triplophysa orientalis*	MT264970
Triplophysa orientalis	MK655278	*Triplophysa orientalis*	MT413248
Triplophysa orientalis	MK655279	*Triplophysa orientalis*	MT413249
Triplophysa orientalis	MK655280	*Triplophysa orientalis*	MT413250
Triplophysa orientalis	MK655281	*Triplophysa orientalis*	MT413251
Triplophysa orientalis	MK655282	*Triplophysa orientalis*	MT413252
Triplophysa orientalis	MK655283	*Triplophysa orientalis*	MT413253
Triplophysa robusta	KX373850	*Triplophysa orientalis*	MT413254
Triplophysa robusta	MG697367	*Triplophysa orientalis*	MT413255
Triplophysa robusta	MG697368	*Triplophysa orientalis*	MT413256
Triplophysa robusta	MG697369	*Triplophysa orientalis*	MK654996

**Species**	**GenBank *cytb***	**Species**	**GenBank *COI***
Triplophysa robusta	MG697370	*Triplophysa orientalis*	MK654997
Triplophysa robusta	MG697371	*Triplophysa orientalis*	MK654998
Triplophysa robusta	MG697372	*Triplophysa robusta*	OQ359361
Triplophysa robusta	MG697373	*Triplophysa robusta*	OQ359362
Triplophysa robusta	MG697374	*Triplophysa robusta*	OQ359363
Triplophysa robusta	MG697375	*Triplophysa robusta*	OQ359364
Triplophysa robusta	MG697376	*Triplophysa robusta*	OQ359365
Triplophysa robusta	MG697377	*Triplophysa robusta*	OQ359366
Triplophysa robusta	MG697378	*Triplophysa robusta*	OQ359367
Triplophysa robusta	MG697384	*Triplophysa robusta*	OQ359368
Triplophysa robusta	MG697386	*Triplophysa robusta*	OQ359369
Triplophysa robusta	MG697387	*Triplophysa robusta*	OQ359370
Triplophysa robusta	MG697388	*Triplophysa robusta*	OQ359371
Triplophysa robusta	MG697389	*Triplophysa robusta*	OQ359372
Triplophysa robusta	MG697390	*Triplophysa robusta*	MN896118
Triplophysa robusta	MG697391	*Triplophysa robusta*	MN896119
Triplophysa robusta	MG697392	*Triplophysa robusta*	MN896120
Triplophysa robusta	MG697393	*Triplophysa robusta*	MN896121
Triplophysa robusta	MG697394	*Triplophysa robusta*	MN896122
Triplophysa robusta	MG697395	*Triplophysa robusta*	MN896123
Triplophysa robusta	MG697396	*Triplophysa robusta*	MN896124
Triplophysa robusta	MG697397	*Triplophysa robusta*	MN896125
Triplophysa robusta	MG697398	*Triplophysa robusta*	MN896126
Triplophysa robusta	MG697399	*Triplophysa robusta*	MN896127
Triplophysa robusta	MG697400	*Triplophysa robusta*	MN896128
Triplophysa robusta	MG697401	*Triplophysa robusta*	MN896129
Triplophysa robusta	MG697402	*Triplophysa robusta*	MN896130
Triplophysa robusta	MG697403	*Triplophysa robusta*	MN896131
Triplophysa robusta	MG697404	*Triplophysa robusta*	MN896132
Triplophysa robusta	MG697405	*Triplophysa robusta*	MN896133
Triplophysa robusta	MG697406	*Triplophysa robusta*	MN896134
Triplophysa robusta	MG697407	*Triplophysa robusta*	MN896135
Triplophysa robusta	MG697408	*Triplophysa robusta*	MN896136
Triplophysa robusta	MG697409	*Triplophysa robusta*	MN896137
Triplophysa robusta	MG697410	*Triplophysa robusta*	MN896138
Triplophysa robusta	MG697411	*Triplophysa robusta*	MN896139
Triplophysa robusta	MG697412	*Triplophysa robusta*	MN896140
Triplophysa robusta	MG697413	*Triplophysa robusta*	MN896141
Triplophysa robusta	MG697414	*Triplophysa robusta*	MN896142
Triplophysa robusta	MG697415	*Triplophysa robusta*	MN896143
Triplophysa robusta	MG697416	*Triplophysa robusta*	MN896144
Triplophysa robusta	MG697417	*Triplophysa robusta*	MN896145
Triplophysa robusta	MG697418	*Triplophysa robusta*	MN896146

**Species**	**GenBank *cytb***	**Species**	**GenBank *COI***
Triplophysa robusta	MG697419	*Triplophysa robusta*	MN896147
Triplophysa robusta	MG697420	*Triplophysa robusta*	MN896148
Triplophysa robusta	MG697421	*Triplophysa robusta*	MN896149
Triplophysa robusta	MG697422	*Triplophysa robusta*	MN896150
Triplophysa robusta	MG697423	*Triplophysa robusta*	MN896151
Triplophysa robusta	MG697424	*Triplophysa robusta*	MN896152
Triplophysa robusta	MG697425	*Triplophysa robusta*	MN896153
Triplophysa robusta	MG697426	*Triplophysa robusta*	MN896154
Triplophysa robusta	MG697427	*Triplophysa robusta*	MN896155
Triplophysa robusta	MG697428	*Triplophysa robusta*	MN896156
Triplophysa robusta	MG697429	*Triplophysa robusta*	MN896157
Triplophysa robusta	MG697430	*Triplophysa robusta*	MN896158
Triplophysa robusta	MG697431	*Triplophysa robusta*	MN896159
Triplophysa robusta	MG697441	*Triplophysa robusta*	MN896160
Triplophysa robusta	MG697443	*Triplophysa robusta*	MN896161
Triplophysa robusta	MG697444	*Triplophysa robusta*	MN896162
Triplophysa robusta	MG697445	*Triplophysa robusta*	MN896163
Triplophysa robusta	MG697457	*Triplophysa robusta*	MN896164
Triplophysa robusta	MG697525	*Triplophysa robusta*	MN896165
Triplophysa robusta	MG697526	*Triplophysa robusta*	MN896166
Triplophysa robusta	MG697527	*Triplophysa robusta*	MN896167
Triplophysa robusta	MG697528	*Triplophysa robusta*	MN896168
Triplophysa robusta	MG697529	*Triplophysa robusta*	MN896169
Triplophysa robusta	MG697530	*Triplophysa robusta*	MN896170
Triplophysa robusta	MG697531	*Triplophysa robusta*	MN896171
Triplophysa robusta	MG697532	*Triplophysa robusta*	MN896172
Triplophysa robusta	MG697533	*Triplophysa robusta*	MN896173
Triplophysa robusta	MG697534	*Triplophysa robusta*	MN896174
Triplophysa robusta	MG697535	*Triplophysa robusta*	MN896175
Triplophysa robusta	MG697536	*Triplophysa robusta*	MN896176
Triplophysa robusta	MG697537	*Triplophysa robusta*	MN896177
Triplophysa robusta	MG697538	*Triplophysa robusta*	MN896178
Triplophysa robusta	MG697539	*Triplophysa robusta*	MN896179
Triplophysa robusta	MG697540	*Triplophysa robusta*	MN896180
Triplophysa robusta	MG697541	*Triplophysa robusta*	MN896181
Triplophysa robusta	MG697542	*Triplophysa robusta*	MN896182
Triplophysa robusta	MG697543	*Triplophysa robusta*	MN896183
Triplophysa robusta	MG697544	*Triplophysa robusta*	MN896184
Triplophysa robusta	MG697545	*Triplophysa robusta*	MN896185
Triplophysa robusta	MG697546	*Triplophysa robusta*	MN896186
Triplophysa robusta	MG697547	*Triplophysa robusta*	MN896187
Triplophysa robusta	MG697548	*Triplophysa robusta*	MN896188
Triplophysa robusta	MG697549	*Triplophysa robusta*	MN896189

**Species**	**GenBank *cytb***	**Species**	**GenBank *COI***
Triplophysa robusta	MG697550	*Triplophysa robusta*	MN896190
Triplophysa robusta	MG697551	*Triplophysa robusta*	MN896191
Triplophysa robusta	MG697552	*Triplophysa robusta*	MN896192
Triplophysa robusta	MG697553	*Triplophysa robusta*	MN896193
Triplophysa robusta	MG697554	*Triplophysa robusta*	MN896194
Triplophysa robusta	MG697555	*Triplophysa robusta*	MN896195
Triplophysa robusta	MG697556	*Triplophysa robusta*	MN896196
Triplophysa robusta	MG697557	*Triplophysa robusta*	MN896197
Triplophysa robusta	MG697558	*Triplophysa robusta*	MN896198
Triplophysa robusta	MG697559	*Triplophysa robusta*	MN896199
Triplophysa robusta	MG697560	*Triplophysa robusta*	MN896200
Triplophysa robusta	MG697561	*Triplophysa robusta*	MN896201
Triplophysa robusta	MG697562	*Triplophysa robusta*	MN896202
Triplophysa robusta	MG697563	*Triplophysa robusta*	MN896203
Triplophysa robusta	MG697564	*Triplophysa robusta*	MN896204
Triplophysa robusta	MG697565	*Triplophysa robusta*	MN896205
Triplophysa robusta	MG697566	*Triplophysa robusta*	MN896206
Triplophysa robusta	MG697567	*Triplophysa robusta*	MN896207
Triplophysa robusta	MG697568	*Triplophysa robusta*	MN896208
Triplophysa robusta	MG697569	*Triplophysa robusta*	MN896209
Triplophysa robusta	MG697570	*Triplophysa robusta*	MN896210
Triplophysa robusta	MG697571	*Triplophysa robusta*	MN896211
Triplophysa robusta	MG697572	*Triplophysa robusta*	MN896212
Triplophysa robusta	MG697573	*Triplophysa robusta*	MN896213
Triplophysa robusta	MG697574	*Triplophysa robusta*	MN896214
Triplophysa robusta	MG697575	*Triplophysa robusta*	MN896215
Triplophysa robusta	MG697576	*Triplophysa robusta*	MN896216
Triplophysa robusta	MG697577	*Triplophysa robusta*	MN896217
Triplophysa robusta	MG697578	*Triplophysa robusta*	MN896218
Triplophysa robusta	MG697579	*Triplophysa robusta*	MN896219
Triplophysa robusta	MG697580	*Triplophysa robusta*	MN896220
Triplophysa robusta	MG697581	*Triplophysa robusta*	MN896221
Triplophysa robusta	MN896385	*Triplophysa robusta*	MN896222
Triplophysa robusta	MN896386	*Triplophysa robusta*	MN896223
Triplophysa robusta	MN896387	*Triplophysa robusta*	MN896224
Triplophysa robusta	MN896388	*Triplophysa robusta*	MN896225
Triplophysa robusta	MN896389	*Triplophysa robusta*	MN896226
Triplophysa robusta	MN896390	*Triplophysa robusta*	MN896227
Triplophysa robusta	MN896391	*Triplophysa robusta*	MN896228
Triplophysa robusta	MN896392	*Triplophysa robusta*	MN896229
Triplophysa robusta	MN896393	*Triplophysa robusta*	MN896232
Triplophysa robusta	MN896394	*Triplophysa robusta*	MK250399
Triplophysa robusta	MN896395	*Triplophysa robusta*	MK250400

**Species**	**GenBank *cytb***	**Species**	**GenBank *COI***
Triplophysa robusta	MN896396	*Triplophysa robusta*	MK250401
Triplophysa robusta	MN896397	*Triplophysa robusta*	MK250403
Triplophysa robusta	MN896398	*Triplophysa robusta*	MK250404
Triplophysa robusta	MN896399	*Triplophysa robusta*	MK250406
Triplophysa robusta	MN896400	*Triplophysa robusta*	MK250407
Triplophysa robusta	MN896401	*Triplophysa robusta*	MK250414
Triplophysa robusta	MN896402	*Triplophysa robusta*	MK250411
Triplophysa robusta	MN896403	*Triplophysa robusta*	MT271455
Triplophysa robusta	MN896404	*Triplophysa robusta*	MT271456
Triplophysa robusta	MN896405	*Triplophysa robusta*	MT271457
Triplophysa robusta	MN896406	*Triplophysa robusta*	MT271458
Triplophysa robusta	MN896407	*Triplophysa robusta*	MT271459
Triplophysa robusta	MN896408	*Triplophysa robusta*	MT271460
Triplophysa robusta	MN896409	*Triplophysa robusta*	MT271461
Triplophysa robusta	MN896410	*Triplophysa robusta*	MT271462
Triplophysa robusta	MN896411	*Triplophysa robusta*	MT271463
Triplophysa robusta	MN896412	*Triplophysa robusta*	MT271464
Triplophysa robusta	MN896413	*Triplophysa robusta*	MT271465
Triplophysa robusta	MN896414	*Triplophysa robusta*	MT271466
Triplophysa robusta	MN896415	*Triplophysa robusta*	MT271467
Triplophysa robusta	MN896416	*Triplophysa robusta*	MT271468
Triplophysa robusta	MN896417	*Triplophysa robusta*	MT271469
Triplophysa robusta	MN896418	*Triplophysa robusta*	MT271470
Triplophysa robusta	MN896419	*Triplophysa robusta*	MT271471
Triplophysa robusta	MN896420	*Triplophysa robusta*	MT271472
Triplophysa robusta	MN896421	*Triplophysa robusta*	MT271473
Triplophysa robusta	MN896422	*Triplophysa robusta*	KU558049
Triplophysa robusta	MN896423	*Triplophysa robusta*	KU558050
Triplophysa robusta	MN896424	*Triplophysa robusta*	KU558051
Triplophysa robusta	MN896425	*Triplophysa robusta*	KU558052
Triplophysa robusta	MN896426	*Triplophysa robusta*	KU558048
Triplophysa robusta	MN896427	*Triplophysa robusta*	KU558053
Triplophysa robusta	MN896428	*Triplophysa robusta*	KU558054
Triplophysa robusta	MN896429	*Triplophysa robusta*	KU558055
Triplophysa robusta	MN896430	*Triplophysa robusta*	KU558056
Triplophysa robusta	MN896431	*Triplophysa robusta*	KU558057
Triplophysa robusta	MN896432	*Triplophysa robusta*	KU558058
Triplophysa robusta	MN896433	*Triplophysa robusta*	MT271474
Triplophysa robusta	MN896434	*Triplophysa robusta*	MT271475
Triplophysa robusta	MN896435	*Triplophysa robusta*	MT271476
Triplophysa robusta	MN896436	*Triplophysa robusta*	MT271477
Triplophysa robusta	MN896437	*Triplophysa robusta*	MT271478
Triplophysa robusta	MN896438	*Triplophysa robusta*	MT271479

**Species**	**GenBank *cytb***	**Species**	**GenBank *COI***
Triplophysa robusta	MN896439	*Triplophysa robusta*	MT271480
Triplophysa robusta	MN896440	*Triplophysa robusta*	MT271481
Triplophysa robusta	MN896441	*Triplophysa robusta*	MT271482
Triplophysa robusta	MN896442	*Triplophysa robusta*	MT271483
Triplophysa robusta	MN896443	*Triplophysa robusta*	MT271484
Triplophysa robusta	MN896444	*Triplophysa robusta*	MT271485
Triplophysa robusta	MN896445	*Triplophysa robusta*	MT271486
Triplophysa robusta	MN896446	*Triplophysa robusta*	MT271487
Triplophysa robusta	MN896447	*Triplophysa robusta*	MT271488
Triplophysa robusta	MN896448	*Triplophysa robusta*	MT271489
Triplophysa robusta	MN896449	*Triplophysa robusta*	MT271490
Triplophysa robusta	MN896450	*Triplophysa robusta*	KU558059
Triplophysa robusta	MN896451	*Triplophysa robusta*	KU558060
Triplophysa robusta	MN896452	*Triplophysa robusta*	MN896230
Triplophysa robusta	MN896453	*Triplophysa robusta*	MN896231
Triplophysa robusta	MN896454	*Triplophysa robusta*	MT271506
Triplophysa robusta	MN896455	*Triplophysa robusta*	MT271507
Triplophysa robusta	MN896456	*Triplophysa robusta*	MT271508
Triplophysa robusta	MN896457	*Triplophysa robusta*	MT271509
Triplophysa robusta	MN896458	*Triplophysa robusta*	MT271510
Triplophysa robusta	MN896459	*Triplophysa robusta*	MT271511
Triplophysa robusta	MN896460	*Triplophysa robusta*	MT271512
Triplophysa robusta	MN896461	*Triplophysa robusta*	MT271513
Triplophysa robusta	MN896462	*Triplophysa robusta*	MT271514
Triplophysa robusta	MN896463	*Triplophysa robusta*	MT271515
Triplophysa robusta	MN896464	*Triplophysa siluroides*	OQ359325
Triplophysa robusta	MN896465	*Triplophysa siluroides*	OQ359326
Triplophysa robusta	MN896466	*Triplophysa siluroides*	OQ359327
Triplophysa robusta	MN896467	*Triplophysa siluroides*	OQ359329
Triplophysa robusta	MN896468	*Triplophysa siluroides*	OQ359330
Triplophysa robusta	MN896469	*Triplophysa siluroides*	MN896108
Triplophysa robusta	MN896470	*Triplophysa siluroides*	MN896109
Triplophysa robusta	MN896471	*Triplophysa siluroides*	MN896110
Triplophysa robusta	MN896472	*Triplophysa siluroides*	MN896111
Triplophysa robusta	MN896473	*Triplophysa siluroides*	MN896112
Triplophysa robusta	MN896474	*Triplophysa siluroides*	MN896113
Triplophysa robusta	MN896475	*Triplophysa siluroides*	MN896114
Triplophysa robusta	MN896476	*Triplophysa siluroides*	MN896115
Triplophysa robusta	MN896477	*Triplophysa siluroides*	MN896116
Triplophysa robusta	MN896478	*Triplophysa siluroides*	MN896117
Triplophysa robusta	MN896479	*Triplophysa siluroides*	MK250391
Triplophysa robusta	MN896480	*Triplophysa siluroides*	OL191031
Triplophysa robusta	MN896481	*Triplophysa siluroides*	MT413261

**Species**	**GenBank *cytb***	**Species**	**GenBank *COI***
Triplophysa robusta	MN896482	*Triplophysa siluroides*	MT271535
Triplophysa robusta	MN896483	*Triplophysa siluroides*	MT271536
Triplophysa robusta	MN896484	*Triplophysa siluroides*	MT413263
Triplophysa robusta	MN896485	*Triplophysa siluroides*	MT271537
Triplophysa robusta	MN896486	*Triplophysa siluroides*	MT271538
Triplophysa robusta	MN896487	*Triplophysa siluroides*	MT271539
Triplophysa robusta	MN896488	*Triplophysa siluroides*	KU558061
Triplophysa robusta	MN896489	*Triplophysa siluroides*	KU558062
Triplophysa robusta	MN896490	*Triplophysa siluroides*	KU558063
Triplophysa robusta	MN896491	*Triplophysa siluroides*	KU558064
Triplophysa robusta	MN896492	*Triplophysa siluroides*	KU558065
Triplophysa robusta	MN896493	*Triplophysa siluroides*	KU558066
Triplophysa robusta	MN896494	*Triplophysa stenura*	KM611456
Triplophysa robusta	MN896495	*Triplophysa stenura*	KM611457
Triplophysa robusta	MN896496	*Triplophysa stenura*	KM611458
Triplophysa robusta	MN896497	*Triplophysa stenura*	KM611459
Triplophysa siluroides	EF212443	*Triplophysa stenura*	KM611460
Triplophysa siluroides	KX373842	*Triplophysa stenura*	KM611461
Triplophysa siluroides	MG697459	*Triplophysa stenura*	KM611462
Triplophysa siluroides	MG697460	*Triplophysa stenura*	KM611463
Triplophysa siluroides	MG697461	*Triplophysa stenura*	KM611464
Triplophysa siluroides	MG697462	*Triplophysa stenura*	KM611465
Triplophysa siluroides	MG697463	*Triplophysa stenura*	KU558076
Triplophysa siluroides	MG697464	*Triplophysa stenura*	KU558077
Triplophysa siluroides	MG697465	*Triplophysa stenura*	KU558078
Triplophysa siluroides	MG697466	*Triplophysa stenura*	KU558079
Triplophysa siluroides	MG697467	*Triplophysa stenura*	KU558080
Triplophysa siluroides	MG697468	*Triplophysa stenura*	KU558081
Triplophysa siluroides	MG697469	*Triplophysa stenura*	KU558082
Triplophysa siluroides	MG697470	*Triplophysa stenura*	KU558083
Triplophysa siluroides	MG697471	*Triplophysa stenura*	KU558084
Triplophysa siluroides	MG697472	*Triplophysa stenura*	KU558085
Triplophysa siluroides	MG697473	*Triplophysa stenura*	KU558086
Triplophysa siluroides	MG697474	*Triplophysa stenura*	KU558087
Triplophysa siluroides	MN896374	*Triplophysa stenura*	KU558088
Triplophysa siluroides	MN896375	*Triplophysa stenura*	KU558089
Triplophysa siluroides	MN896376	*Triplophysa stenura*	KU558090
Triplophysa siluroides	MN896377	*Triplophysa stenura*	KU558091
Triplophysa siluroides	MN896378	*Triplophysa stenura*	KU558092
Triplophysa siluroides	MN896379	*Triplophysa stenura*	KU558093
Triplophysa siluroides	MN896380	*Triplophysa stenura*	KU558094
Triplophysa siluroides	MN896381	*Triplophysa stenura*	KU558095
Triplophysa siluroides	MN896382	*Triplophysa stenura*	KU558096

Species	GenBank *cytb*	Species	GenBank *COI*
Triplophysa siluroides	MN896383	*Triplophysa stenura*	KU558097
Triplophysa siluroides	MN896384	*Triplophysa stenura*	KU558098
Triplophysa stenura	JN837657	*Triplophysa stenura*	KU558099
Triplophysa stenura	DQ105246	*Triplophysa stenura*	KU558100
Triplophysa stenura	DQ105247	*Triplophysa stenura*	KU558101
Triplophysa stenura	MG697583	*Triplophysa stenura*	KU558102
Triplophysa stenura	MK655187	*Triplophysa stenura*	KU558103
Triplophysa stenura	MK655188	*Triplophysa stenura*	KU558104
Triplophysa stenura	MK655189	*Triplophysa stenura*	KU558105
Triplophysa stenura	MK655190	*Triplophysa stenura*	KU558106
Triplophysa stenura	MK655191	*Triplophysa stenura*	KU558107
Triplophysa stenura	MK655192	*Triplophysa stenura*	MF123353
Triplophysa stenura	MK655193	*Triplophysa stenura*	MT413264
Triplophysa stenura	MK655194	*Triplophysa stenura*	MT413265
Triplophysa stenura	MK655195	*Triplophysa stenura*	MT413266
Triplophysa stenura	MK655196	*Triplophysa stenura*	MT413267
Triplophysa stenura	MK655197	*Triplophysa stenura*	MT413268
Triplophysa stenura	MK655198	*Triplophysa stenura*	MT413269
Triplophysa stenura	MK655199	*Triplophysa stenura*	MT413270
Triplophysa stenura	MK655200	*Triplophysa stenura*	MT413271
Triplophysa stenura	MK655201	*Triplophysa stenura*	MT413272
Triplophysa stenura	MK655202	*Triplophysa stenura*	MT413273
Triplophysa stenura	MK655203	*Triplophysa stenura*	MT413274
Triplophysa stenura	MK655204	*Triplophysa stenura*	MT413275
Triplophysa stenura	MK655205	*Triplophysa stenura*	MK654971
Triplophysa stenura	MK655206	*Triplophysa stenura*	MK654972
Triplophysa stenura	MK655207	*Triplophysa stenura*	MK654973
Triplophysa stenura	MK655208	*Triplophysa stenura*	MK654974
Triplophysa stenura	MK655209	*Triplophysa stenura*	MK654975
Triplophysa stenura	MK655210	*Triplophysa stenura*	MK654976
Triplophysa stenura	MK655211	*Triplophysa stenura*	MK654977
Triplophysa stenura	MK655212	*Triplophysa stenura*	MK654978
Triplophysa stenura	MK655213	*Triplophysa stenura*	MK654979
Triplophysa stenura	MK655214	*Triplophysa stenura*	MK654980
Triplophysa stenura	MK655215	*Triplophysa stenura*	MK654981
Triplophysa stenura	MK655216	*Triplophysa stenura*	MK654982
Triplophysa stenura	MK655217	*Triplophysa stenura*	MK654983
Triplophysa stenura	MK655218	*Triplophysa stenura*	MK654984
Triplophysa stenura	MK655219	*Triplophysa stenura*	MK654985
Triplophysa stenura	MK655220	*Triplophysa stenura*	MK654986
Triplophysa stenura	MK655221	*Triplophysa stenura*	MK654987
Triplophysa stenura	MK655222	*Triplophysa stenura*	MK654988
Triplophysa stenura	MK655223	*Triplophysa stenura*	MK654989

Species	GenBank *cytb*	Species	GenBank *COI*
Triplophysa stenura	MK655224	*Triplophysa stenura*	MK654990
Triplophysa stenura	MK655225	*Triplophysa stenura*	MK654991
Triplophysa stenura	MK655226	*Triplophysa stenura*	MK654992
Triplophysa stenura	MK655227	*Triplophysa stenura*	MK654993
Triplophysa stenura	MK655228	*Triplophysa stenura*	MK654994
Triplophysa stenura	MK655229	*Triplophysa stenura*	MK654995
Triplophysa stenura	MK655230	*Triplophysa stenura*	MT681383
Triplophysa stenura	MK655231	*Triplophysa stenura*	MT681384
Triplophysa stenura	MK655232	*Triplophysa stenura*	MT681385
Triplophysa stenura	MK655233	*Triplophysa stenura*	MT681386
Triplophysa stenura	MK655234	*Triplophysa stenura*	MT681387
Triplophysa stenura	MK655235	*Triplophysa stenura*	MT681388
Triplophysa stenura	MK655236	*Triplophysa stenura*	MT681389
Triplophysa stenura	MK655237	*Triplophysa stenura*	MT681390
Triplophysa stenura	MK655238	*Triplophysa stenura*	MT681391
Triplophysa stenura	MK655239	*Triplophysa stenura*	MT681392
Triplophysa stenura	MK655240	*Triplophysa stenura*	MT681393
Triplophysa stenura	MK655241	*Triplophysa stenura*	MT681394
Triplophysa stenura	MK655242	*Triplophysa stenura*	MT681395
Triplophysa stenura	MK655243	*Triplophysa stenura*	MT681396
Triplophysa stenura	MK655244	*Triplophysa stenura*	MT681397
Triplophysa stenura	MK655245	*Triplophysa stenura*	MT681398
Triplophysa stenura	MK655246	*Triplophysa stenura*	MT681399
Triplophysa stenura	MK655247	*Triplophysa stenura*	MT681400
Triplophysa stenura	MK655248	*Triplophysa stenura*	MT681401
Triplophysa stenura	MK655249	*Triplophysa stenura*	MT681402
Triplophysa stenura	MK655250	*Triplophysa stenura*	MT681403
Triplophysa stenura	MK655251	*Triplophysa stenura*	MT681404
Triplophysa stenura	MK655252	*Triplophysa stenura*	MT681405
Triplophysa stenura	MK655253	*Triplophysa stenura*	MT681406
Triplophysa stenura	MK655254	*Triplophysa stenura*	MT681407
Triplophysa stenura	MK655255	*Triplophysa stenura*	MT681408
Triplophysa stenura	MK655256	*Triplophysa stenura*	MT681409
Triplophysa stenura	MK655257	*Triplophysa stenura*	MT681410
Triplophysa stenura	MK655258	*Triplophysa stenura*	MT681411
Triplophysa stenura	MK655259	*Triplophysa stenura*	MT681412
Triplophysa stenura	MK655260	*Triplophysa stenura*	MT681413
Triplophysa stenura	MK655261	*Triplophysa stenura*	MT681414
Triplophysa stenura	MK655262	*Triplophysa stenura*	MT681415
Triplophysa stenura	MK655263	*Triplophysa stenura*	MT681416
Triplophysa stenura	MK655264	*Triplophysa stenura*	MT681417
Triplophysa stenura	MK655265	*Triplophysa stenura*	MT681418
Triplophysa stenura	MK655266	*Triplophysa stenura*	MT681419

**Species**	**GenBank *cytb***	**Species**	**GenBank *COI***
Triplophysa stenura	MK655267	*Triplophysa stenura*	MT681420
Triplophysa stenura	MK655268	*Triplophysa stenura*	MT681421
Triplophysa stenura	KJ650344	*Triplophysa stenura*	MT681422
Triplophysa stenura	KJ650345	*Triplophysa stenura*	MT681423
Triplophysa stenura	KJ650346	*Triplophysa stenura*	MT681424
Triplophysa stenura	KJ650347	*Triplophysa stenura*	MT681425
Triplophysa stenura	KJ650348	*Triplophysa stenura*	MT681426
Triplophysa stenura	KJ650349	*Triplophysa stenura*	MT681427
Triplophysa stenura	KJ650350	*Triplophysa stenura*	MT681428
Triplophysa stenura	KJ650351	*Triplophysa stenura*	MT681429
Triplophysa stenura	KJ650352	*Triplophysa stenura*	MT681430
Triplophysa stenura	KJ650353	*Triplophysa stenura*	MT681431
Triplophysa stenura	KJ650354	*Triplophysa stenura*	MT681432
Triplophysa stenura	KJ650355	*Triplophysa stoliczkai*	OQ359331
Triplophysa stenura	KJ650356	*Triplophysa stoliczkai*	OQ359332
Triplophysa stenura	KJ650357	*Triplophysa stoliczkai*	OQ359333
Triplophysa stenura	KJ650358	*Triplophysa stoliczkai*	OQ359334
Triplophysa stenura	KJ650359	*Triplophysa stoliczkai*	OQ359335
Triplophysa stenura	KJ650360	*Triplophysa stoliczkai*	OQ359337
Triplophysa stenura	KJ650361	*Triplophysa stoliczkai*	OQ359338
Triplophysa stenura	KJ650362	*Triplophysa stoliczkai*	MF123373
Triplophysa stenura	KJ650363	*Triplophysa stoliczkai*	MF123374
Triplophysa stenura	KJ650364	*Triplophysa stoliczkai*	MF123375
Triplophysa stenura	KJ650365	*Triplophysa stoliczkai*	MF123376
Triplophysa stenura	KJ650366	*Triplophysa stoliczkai*	MF123377
Triplophysa stenura	KJ650367	*Triplophysa stoliczkai*	MF123368
Triplophysa stenura	KJ650368	*Triplophysa stoliczkai*	MF123367
Triplophysa stenura	KJ650369	*Triplophysa stoliczkai*	MF123366
Triplophysa stenura	KJ650370	*Triplophysa stoliczkai*	MF123365
Triplophysa stenura	KJ650371	*Triplophysa stoliczkai*	MF123364
Triplophysa stenura	KJ650372	*Triplophysa stoliczkai*	MF123363
Triplophysa stenura	KJ650373	*Triplophysa stoliczkai*	MF123391
Triplophysa stenura	MT681433	*Triplophysa stoliczkai*	MF123390
Triplophysa stenura	MT681434	*Triplophysa stoliczkai*	MF123389
Triplophysa stenura	MT681435	*Triplophysa stoliczkai*	MF123388
Triplophysa stenura	MT681436	*Triplophysa stoliczkai*	MF123387
Triplophysa stenura	MT681437	*Triplophysa stoliczkai*	MF123386
Triplophysa stenura	MT681438	*Triplophysa stoliczkai*	MF123385
Triplophysa stenura	MT681439	*Triplophysa stoliczkai*	MF123384
Triplophysa stenura	MT681440	*Triplophysa stoliczkai*	MF123383
Triplophysa stenura	MT681441	*Triplophysa stoliczkai*	MF123382
Triplophysa stenura	MT681442	*Triplophysa stoliczkai*	MF123362
Triplophysa stenura	MT681443	*Triplophysa stoliczkai*	MF123361

**Species**	**GenBank *cytb***	**Species**	**GenBank *COI***
Triplophysa stenura	MT681444	*Triplophysa stoliczkai*	MF123360
Triplophysa stenura	MT681445	*Triplophysa stoliczkai*	MF123359
Triplophysa stenura	MT681446	*Triplophysa stoliczkai*	MF123358
Triplophysa stenura	MT681447	*Triplophysa stoliczkai*	MF123357
Triplophysa stenura	MT681448	*Triplophysa stoliczkai*	MF123356
Triplophysa stenura	MT681449	*Triplophysa stoliczkai*	MF123355
Triplophysa stenura	MT681450	*Triplophysa stoliczkai*	MF123354
Triplophysa stenura	MT681451	*Triplophysa stoliczkai*	MF123381
Triplophysa stenura	MT681452	*Triplophysa stoliczkai*	MF123380
Triplophysa stenura	MT681453	*Triplophysa stoliczkai*	MF123379
Triplophysa stenura	MT681454	*Triplophysa stoliczkai*	MF123378
Triplophysa stenura	MT681455	*Triplophysa stoliczkai*	MT271542
Triplophysa stenura	MT681456	*Triplophysa stoliczkai*	MK654915
Triplophysa stenura	MT681457	*Triplophysa stoliczkai*	MT413291
Triplophysa stenura	MT681458	*Triplophysa stoliczkai*	MK654924
Triplophysa stenura	MT681459	*Triplophysa stoliczkai*	MT271543
Triplophysa stenura	MT681460	*Triplophysa stoliczkai*	MT271544
Triplophysa stenura	MT681461	*Triplophysa stoliczkai*	MT271545
Triplophysa stenura	MT681462	*Triplophysa stoliczkai*	MK654925
Triplophysa stenura	MT681463	*Triplophysa stoliczkai*	MT271546
Triplophysa stenura	MT681464	*Triplophysa stoliczkai*	MT271547
Triplophysa stenura	MT681465	*Triplophysa stoliczkai*	MT271548
Triplophysa stenura	MT681466	*Triplophysa stoliczkai*	MT271549
Triplophysa stenura	MT681467	*Triplophysa stoliczkai*	MT271550
Triplophysa stenura	MT681468	*Triplophysa stoliczkai*	MT271551
Triplophysa stenura	MT681469	*Triplophysa stoliczkai*	MT271552
Triplophysa stenura	MT681470	*Triplophysa stoliczkai*	MT271553
Triplophysa stenura	MT681471	*Triplophysa stoliczkai*	MT271554
Triplophysa stenura	MT681472	*Triplophysa stoliczkai*	MT271555
Triplophysa stenura	MT681473	*Triplophysa stoliczkai*	MT271556
Triplophysa stenura	MT681474	*Triplophysa stoliczkai*	MK654916
Triplophysa stenura	MT681475	*Triplophysa stoliczkai*	MT413292
Triplophysa stenura	MT681476	*Triplophysa stoliczkai*	MT271557
Triplophysa stenura	MT681477	*Triplophysa stoliczkai*	MT271558
Triplophysa stenura	MT681478	*Triplophysa stoliczkai*	MK654917
Triplophysa stenura	MT681479	*Triplophysa stoliczkai*	MT413293
Triplophysa stenura	MT681480	*Triplophysa stoliczkai*	MK654918
Triplophysa stenura	MT681481	*Triplophysa stoliczkai*	MT413294
Triplophysa stenura	MT681482	*Triplophysa stoliczkai*	MK654919
Triplophysa stenura	MT681483	*Triplophysa stoliczkai*	MT413295
Triplophysa stenura	MT681484	*Triplophysa stoliczkai*	MK654920
Triplophysa stenura	MT681485	*Triplophysa stoliczkai*	MK654921
Triplophysa stenura	MT681486	*Triplophysa stoliczkai*	MK654922

**Species**	**GenBank *cytb***	**Species**	**GenBank *COI***
Triplophysa stenura	MT681487	*Triplophysa stoliczkai*	MK654923
Triplophysa stenura	MT681488	*Triplophysa stoliczkai*	MT271559
Triplophysa stenura	MT681489	*Triplophysa stoliczkai*	MT271560
Triplophysa stenura	MT681490	*Triplophysa stoliczkai*	MT271561
Triplophysa stenura	MT681491	*Triplophysa stoliczkai*	MT271562
Triplophysa stenura	MT681492	*Triplophysa stoliczkai*	MT271563
Triplophysa stenura	MT681493	*Triplophysa stoliczkai*	KU558110
Triplophysa stenura	MT681494	*Triplophysa stoliczkai*	KU558111
Triplophysa stenura	MT681495	*Triplophysa stoliczkai*	KU558112
Triplophysa stenura	MT681496	*Triplophysa stoliczkai*	KU558113
Triplophysa stenura	MT681497	*Triplophysa stoliczkai*	KU558114
Triplophysa stenura	MT681498	*Triplophysa stoliczkai*	KU558115
Triplophysa stenura	MT681499	*Triplophysa stoliczkai*	KU558116
Triplophysa stenura	MT681500	*Triplophysa stoliczkai*	KU558117
Triplophysa stenura	MT681501	*Triplophysa stoliczkai*	KU558118
Triplophysa stenura	MT681502	*Triplophysa stoliczkai*	KU558119
Triplophysa stenura	MT681503	*Triplophysa stoliczkai*	MT271564
Triplophysa stenura	MT681504	*Triplophysa stoliczkai*	MT271565
Triplophysa stenura	MT681505	*Triplophysa stoliczkai*	MT271566
Triplophysa stenura	MT681506		
Triplophysa stenura	MT681507		
Triplophysa stenura	MT681508		
Triplophysa stenura	MT681509		
Triplophysa stenura	MT681510		
Triplophysa stenura	MT681511		
Triplophysa stenura	MT681512		
Triplophysa stenura	MT681513		
Triplophysa stenura	MT681514		
Triplophysa stenura	MT681515		
Triplophysa stenura	MT681516		
Triplophysa stenura	MT681517		
Triplophysa stenura	MT681518		
Triplophysa stenura	MT681519		
Triplophysa stenura	MT681520		
Triplophysa stenura	MT681521		
Triplophysa stenura	MT681522		
Triplophysa stenura	MT681523		
Triplophysa stenura	MT681524		
Triplophysa stenura	MT681525		
Triplophysa stenura	MT681526		
Triplophysa stenura	MT681527		
Triplophysa stenura	MT681528		
Triplophysa stenura	MT681529		

**Species**	**GenBank *cytb***	**Species**	**GenBank *COI***
Triplophysa stenura	MT681530		
Triplophysa stenura	MT681531		
Triplophysa stenura	MT681532		
Triplophysa stenura	MT681533		
Triplophysa stenura	MT681534		
Triplophysa stenura	MT681535		
Triplophysa stenura	MT681536		
Triplophysa stenura	MT681537		
Triplophysa stenura	MT681538		
Triplophysa stenura	MT681539		
Triplophysa stenura	MT681540		
Triplophysa stenura	MT681541		
Triplophysa stenura	MT681542		
Triplophysa stenura	MT681543		
Triplophysa stenura	MT681544		
Triplophysa stenura	MT681545		
Triplophysa stenura	MT681546		
Triplophysa stenura	MT681547		
Triplophysa stenura	MT681548		
Triplophysa stenura	MT681549		
Triplophysa stenura	MT681550		
Triplophysa stenura	MT681551		
Triplophysa stenura	MT681552		
Triplophysa stenura	MT681553		
Triplophysa stenura	MT681554		
Triplophysa stenura	MT681555		
Triplophysa stenura	MT681556		
Triplophysa stenura	MT681557		
Triplophysa stenura	MT681558		
Triplophysa stenura	MT681559		
Triplophysa stenura	MT681560		
Triplophysa stenura	MT681561		
Triplophysa stenura	MT681562		
Triplophysa stenura	MT681563		
Triplophysa stenura	MT681564		
Triplophysa stenura	MT681565		
Triplophysa stenura	MT681566		
Triplophysa stenura	MT681567		
Triplophysa stenura	MT681568		
Triplophysa stenura	MT681569		
Triplophysa stenura	MT681570		
Triplophysa stenura	MT681571		
Triplophysa stenura	MT681572		

**Species**	**GenBank *cytb***	**Species**	**GenBank *COI***
Triplophysa stenura	MT681573		
Triplophysa stenura	MT681574		
Triplophysa stenura	MT681575		
Triplophysa stenura	MT681576		
Triplophysa stenura	MT681577		
Triplophysa stenura	MT681578		
Triplophysa stenura	MT681579		
Triplophysa stoliczkai	KX373851		
Triplophysa stoliczkai	MG697582		
Triplophysa stoliczkai	MG725380		
Triplophysa stoliczkai	MG725381		
Triplophysa stoliczkai	MG725382		
Triplophysa stoliczkai	MG725394		
Triplophysa stoliczkai	MG725395		
Triplophysa stoliczkai	MG725398		
Triplophysa stoliczkai	MG725399		
Triplophysa stoliczkai	MG725396		
Triplophysa stoliczkai	MG725397		
Triplophysa stoliczkai	MG725400		
Triplophysa stoliczkai	MG725404		
Triplophysa stoliczkai	MG725405		
Triplophysa stoliczkai	MG725406		
Triplophysa stoliczkai	MG725407		
Triplophysa stoliczkai	MG725409		
Triplophysa stoliczkai	MG725410		
Triplophysa stoliczkai	MG725388		
Triplophysa stoliczkai	MG725389		
Triplophysa stoliczkai	MG725390		
Triplophysa stoliczkai	MG725391		
Triplophysa stoliczkai	MG725392		
Triplophysa stoliczkai	MG725393		
Triplophysa stoliczkai	MG725408		
Triplophysa stoliczkai	MG725383		
Triplophysa stoliczkai	MG725384		
Triplophysa stoliczkai	MK655046		
Triplophysa stoliczkai	MK655055		
Triplophysa stoliczkai	MK655056		
Triplophysa stoliczkai	MK655057		
Triplophysa stoliczkai	MK655058		
Triplophysa stoliczkai	MK655059		
Triplophysa stoliczkai	MK655060		
Triplophysa stoliczkai	MK655061		
Triplophysa stoliczkai	MK655062		

**Species**	**GenBank *cytb***	**Species**	**GenBank *COI***
Triplophysa stoliczkai	MK655063		
Triplophysa stoliczkai	MK655064		
Triplophysa stoliczkai	MK655047		
Triplophysa stoliczkai	MK655065		
Triplophysa stoliczkai	MK655066		
Triplophysa stoliczkai	MK655067		
Triplophysa stoliczkai	MK655068		
Triplophysa stoliczkai	MK655069		
Triplophysa stoliczkai	MK655070		
Triplophysa stoliczkai	MK655071		
Triplophysa stoliczkai	MK655072		
Triplophysa stoliczkai	MK655073		
Triplophysa stoliczkai	MK655074		
Triplophysa stoliczkai	MK655048		
Triplophysa stoliczkai	MK655075		
Triplophysa stoliczkai	MK655049		
Triplophysa stoliczkai	MK655050		
Triplophysa stoliczkai	MK655051		
Triplophysa stoliczkai	MK655052		
Triplophysa stoliczkai	MK655053		
Triplophysa stoliczkai	MK655054		

Table S3. Geographic occurrence records (longitude and latitude) of 12 *Triplophysa* species used for MaxEnt modeling.

Species	Longitude	Latitude
T. bleekeri	104.2878	33.1516
T. bleekeri	105.4132	33.4480
T. bleekeri	106.4269	33.9323
T. bleekeri	106.1483	34.2816
T. bleekeri	104.8730	32.8490
T. bleekeri	101.0194	30.0701
T. bleekeri	105.3600	28.7600
T. bleekeri	103.5614	29.7418
T. bleekeri	105.2300	32.5700
T. bleekeri	102.9800	29.9800
T. bleekeri	103.0833	29.9833
T. bleekeri	102.1842	28.5781
T. bleekeri	104.2842	34.1559
T. bleekeri	104.9210	33.3987
T. bleekeri	104.6791	32.9487
T. bleekeri	108.8746	31.3866
T. bleekeri	110.1094	32.0076
T. bleekeri	103.1716	30.6751
T. bleekeri	104.2204	30.5825
T. bleekeri	102.8100	30.3600
T. bleekeri	104.6911	38.0733
T. bleekeri	103.3100	31.5500
T. bleekeri	102.9928	29.7767
T. bleekeri	102.8858	30.0419
T. bleekeri	103.7925	31.2417
T. brevicauda	86.8990	28.6100
T. brevicauda	98.8200	31.2100
T. brevicauda	101.7932	30.9113
T. brevicauda	86.4583	28.6590
T. brevicauda	91.9694	29.9585
T. brevicauda	91.3894	31.9911
T. brevicauda	91.6128	30.0744
T. brevicauda	79.8987	33.4995
T. brevicauda	81.3310	31.4973
T. brevicauda	85.6214	28.8901
T. brevicauda	83.3972	30.0288
T. brevicauda	94.3532	29.6669
T. brevicauda	94.4362	32.3141
T. brevicauda	88.8751	29.3231
T. brevicauda	98.5900	29.6800

Species	Longitude	Latitude
T. brevicauda	101.8000	30.9000
T. brevicauda	104.6911	38.0733
T. dalaica	107.0460	35.1795
T. dalaica	108.2514	35.8519
T. dalaica	108.4963	36.2124
T. dalaica	108.4590	36.2455
T. dalaica	108.1697	36.3926
T. dalaica	106.6139	35.3100
T. dalaica	105.7407	35.4141
T. dalaica	106.5076	35.6484
T. dalaica	107.7414	35.3491
T. dalaica	107.6733	35.4312
T. dalaica	108.4074	35.4399
T. dalaica	107.1053	35.7356
T. dalaica	113.8388	41.7039
T. dalaica	113.8388	41.7039
T. dalaica	113.8388	41.7039
T. dalaica	116.4309	40.3562
T. dalaica	116.5706	40.2078
T. dalaica	116.4444	40.2796
T. dalaica	116.5359	40.2979
T. dalaica	116.4682	40.2749
T. dalaica	116.4904	40.3907
T. dalaica	116.4193	40.3485
T. dalaica	114.1279	36.0450
T. dalaica	113.2745	39.5799
T. dalaica	113.3391	39.7992
T. dalaica	113.0954	39.5375
T. dalaica	114.1011	39.9758
T. dalaica	112.3109	39.0060
T. dalaica	114.0419	39.9747
T. dalaica	112.3127	39.0570
T. dalaica	113.2494	40.3793
T. dalaica	113.1902	40.4322
T. dalaica	113.2104	40.5217
T. dalaica	113.6393	39.7039
T. dalaica	112.5602	39.3874
T. dalaica	113.9193	40.0008
T. dalaica	112.5912	39.3677
T. dalaica	113.1168	40.1160
T. dalaica	113.3008	40.3166
T. dalaica	114.1304	39.9952
T. dalaica	113.1816	39.6875

Species	Longitude	Latitude
T. dalaica	115.0367	40.3316
T. dalaica	115.6013	39.6405
T. hsutschouensis	102.9274	37.3761
T. hsutschouensis	102.2191	37.9185
T. hsutschouensis	100.3973	39.1396
T. hsutschouensis	102.4318	37.5160
T. hsutschouensis	102.0450	38.0360
T. hsutschouensis	101.3935	38.0599
T. hsutschouensis	102.0272	38.3315
T. hsutschouensis	96.7497	39.8683
T. hsutschouensis	97.4840	40.2072
T. hsutschouensis	103.1833	34.0853
T. hsutschouensis	94.3460	39.9410
T. hsutschouensis	95.9480	40.5100
T. hsutschouensis	96.9910	40.4630
T. hsutschouensis	96.7630	39.9980
T. hsutschouensis	97.9770	39.1450
T. hsutschouensis	99.5230	38.7750
T. hsutschouensis	99.5890	38.8130
T. hsutschouensis	100.1390	38.3390
T. hsutschouensis	102.8320	38.0550
T. hsutschouensis	102.5570	37.8270
T. leptosoma	98.0112	38.7025
T. leptosoma	96.7497	39.8683
T. leptosoma	95.3950	34.5540
T. leptosoma	101.6680	37.3790
T. leptosoma	103.1500	37.3000
T. leptosoma	100.3833	38.0667
T. leptosoma	99.6500	38.4000
T. leptosoma	100.1000	38.7500
T. leptosoma	98.4167	38.6667
T. leptosoma	98.8333	38.8333
T. leptosoma	96.5167	40.5333
T. leptosoma	96.0000	39.5000
T. leptosoma	94.8333	36.5000
T. leptosoma	99.0667	36.7667
T. leptosoma	95.4833	37.6333
T. leptosoma	96.1833	36.5000
T. leptosoma	94.8667	36.4167
T. leptosoma	94.3833	36.4333
T. minxianensis	102.8514	36.3766
T. minxianensis	108.1697	36.3926
T. minxianensis	104.8696	33.8313

Species	Longitude	Latitude
T. minxianensis	104.7210	34.8741
T. minxianensis	105.9917	34.4011
T. minxianensis	104.0678	34.4027
T. minxianensis	105.9604	34.6093
T. minxianensis	104.1938	34.7877
T. minxianensis	105.5867	34.1240
T. minxianensis	105.9628	34.2228
T. minxianensis	105.2823	34.5314
T. minxianensis	105.1811	34.5789
T. minxianensis	105.3650	34.6284
T. minxianensis	104.2159	35.0830
T. minxianensis	104.2132	35.0148
T. minxianensis	104.8286	34.5676
T. minxianensis	104.9923	34.6277
T. minxianensis	104.7237	34.7138
T. minxianensis	103.1519	35.8272
T. minxianensis	106.5117	34.8041
T. minxianensis	104.5024	34.6525
T. minxianensis	104.3771	34.8687
T. minxianensis	105.9582	35.3236
T. minxianensis	103.8153	34.9932
T. minxianensis	105.9604	34.6093
T. minxianensis	104.1938	34.7877
T. minxianensis	105.2823	34.5314
T. minxianensis	105.1811	34.5789
T. minxianensis	105.3650	34.6284
T. minxianensis	106.5117	34.8041
T. minxianensis	105.9582	35.3236
T. obscura	105.6483	33.7006
T. obscura	105.7662	33.7131
T. obscura	105.1801	34.7598
T. obscura	103.0074	34.6742
T. obscura	106.2103	33.6343
T. obscura	105.8997	33.8873
T. obscura	105.4132	33.4480
T. obscura	104.8696	33.8313
T. obscura	105.0795	33.9334
T. obscura	105.2657	34.2119
T. obscura	106.1483	34.2816
T. obscura	104.1938	34.7877
T. obscura	105.5867	34.1240
T. obscura	105.5856	34.2207
T. obscura	105.9011	34.2587

Species	Longitude	Latitude
T. obscura	105.6076	34.3193
T. obscura	105.1811	34.5789
T. obscura	105.3650	34.6284
T. obscura	104.3814	33.9884
T. obscura	104.2269	34.9883
T. obscura	104.9923	34.6277
T. obscura	105.3810	33.8090
T. obscura	104.5024	34.6525
T. obscura	103.8153	34.9932
T. orientali	103.0113	34.7564
T. orientali	103.0350	33.3290
T. orientali	96.4284	36.5327
T. orientali	91.7622	29.2641
T. orientali	91.2823	29.6760
T. orientali	79.7662	33.4815
T. orientali	78.9035	33.9863
T. orientali	102.9426	33.6059
T. orientali	102.4804	33.5537
T. orientali	101.5396	30.3103
T. orientali	101.8042	30.9067
T. orientali	100.3624	29.6991
T. orientali	101.0140	32.1517
T. orientali	101.2933	32.2179
T. orientali	101.5000	30.5000
T. orientali	95.2979	37.8111
T. orientali	101.3300	32.2400
T. orientali	100.3700	29.7020
T. orientali	101.2984	30.4073
T. orientali	101.2907	30.3805
T. orientali	101.8000	30.9000
T. orientali	95.7000	34.0000
T. orientali	96.4000	33.9000
T. orientali	96.2000	34.1000
T. orientali	91.1000	30.2000
T. orientali	90.7000	29.0000
T. orientali	104.7000	38.1000
T. robusta	107.0460	35.1795
T. robusta	103.4118	34.0113
T. robusta	103.1738	34.1589
T. robusta	103.8743	34.1880
T. robusta	102.9274	37.3761
T. robusta	102.6396	37.5004
T. robusta	103.0074	34.6742

**Species**	**Longitude**	**Latitude**
T. robusta	103.0113	34.7564
T. robusta	104.2878	33.1516
T. robusta	103.6053	34.9552
T. robusta	103.0220	35.4600
T. robusta	105.0512	33.7885
T. robusta	105.0795	33.9334
T. robusta	105.2657	34.2119
T. robusta	104.0678	34.4027
T. robusta	104.0826	34.4940
T. robusta	104.4219	33.9608
T. robusta	104.2132	35.0148
T. robusta	104.8545	33.0405
T. robusta	105.1896	33.2732
T. robusta	102.1320	34.8521
T. robusta	103.4173	36.3116
T. robusta	102.0272	38.3315
T. robusta	104.0138	35.8643
T. robusta	103.9566	33.9185
T. robusta	103.5851	34.5398
T. robusta	103.1844	34.6185
T. robusta	103.8153	34.9932
T. robusta	102.1220	37.1770
T. robusta	100.8810	37.5780
T. robusta	101.0000	36.9000
T. robusta	101.6540	36.9770
T. robusta	103.2780	36.6970
T. robusta	103.8250	35.3350
T. robusta	104.0570	34.4730
T. robusta	102.3440	34.6040
T. robusta	103.5220	33.9730
T. robusta	104.3250	33.7950
T. robusta	104.3670	33.0990
T. robusta	104.8720	32.8490
T. robusta	105.7760	33.1280
T. robusta	112.2400	35.2150
T. robusta	110.9520	34.4030
T. robusta	111.4230	34.0430
T. robusta	111.6970	33.9390
T. robusta	111.9800	34.0640
T. robusta	111.2110	34.2400
T. robusta	112.0160	34.3760
T. robusta	112.0730	34.6100
T. robusta	111.0550	33.9510

**Species**	**Longitude**	**Latitude**
T. robusta	110.8520	34.0640
T. robusta	103.6700	33.5600
T. robusta	108.2514	35.8519
T. robusta	108.4590	36.2455
T. robusta	108.1697	36.3926
T. robusta	106.6139	35.3100
T. robusta	107.4151	35.2268
T. robusta	107.5415	35.3827
T. robusta	106.5076	35.6484
T. robusta	107.5955	35.0711
T. robusta	107.7414	35.3491
T. robusta	107.6733	35.4312
T. robusta	108.4923	35.3846
T. robusta	108.4074	35.4399
T. robusta	107.1053	35.7356
T. siluroides	103.0373	35.4713
T. siluroides	102.1164	33.7788
T. siluroides	101.4249	37.4574
T. siluroides	104.0678	34.4027
T. siluroides	104.3948	37.3418
T. siluroides	103.1519	35.8272
T. siluroides	103.8153	34.9932
T. siluroides	100.3070	33.7460
T. siluroides	110.8275	34.4046
T. siluroides	102.2833	33.4167
T. siluroides	95.0939	38.8554
T. siluroides	94.1754	39.0394
T. siluroides	101.4249	37.4574
T. siluroides	100.5825	38.0286
T. siluroides	99.5826	38.4250
T. siluroides	100.3973	39.1396
T. siluroides	98.0112	38.7025
T. siluroides	100.1243	38.6352
T. siluroides	98.4117	38.8145
T. siluroides	102.0272	38.3315
T. siluroides	96.7497	39.8683
T. stenura	93.9700	35.5100
T. stenura	90.8700	32.0300
T. stenura	91.5500	31.5000
T. stenura	89.8400	32.1300
T. stenura	91.1500	29.6400
T. stenura	92.6800	29.1900
T. stenura	85.1500	29.3200

Species	Longitude	Latitude
T. stenura	83.9800	29.7600
T. stenura	89.1600	28.0300
T. stenura	89.0300	27.3900
T. stenura	92.3400	28.4100
T. stenura	87.8700	28.1700
T. stenura	86.6100	28.5800
T. stenura	86.4500	28.6300
T. stenura	85.5900	28.7700
T. stenura	82.3000	30.6100
T. stenura	82.2100	31.3100
T. stenura	83.9800	31.0500
T. stenura	92.4300	34.2100
T. stenura	99.9800	27.9990
T. stenura	99.7940	27.3420
T. stenura	99.7510	28.9700
T. stenura	99.2090	30.1550
T. stenura	98.8400	31.2100
T. stenura	99.9080	26.8280
T. stenura	91.8280	32.0680
T. stenura	98.6090	27.7990
T. stenura	96.9390	30.0550
T. stenura	92.0420	31.3690
T. stenura	97.2100	31.1400
T. stenura	100.0230	25.6290
T. stenura	96.4290	32.3970
T. stenura	83.4171	30.0116
T. stenura	87.1426	28.6122
T. stenura	91.2740	29.6756
T. stenura	101.1871	32.9896
T. stenura	98.9030	26.4860
T. stenura	98.7280	27.6680
T. stenura	98.6300	28.0300
T. stenura	96.2051	34.0988
T. stenura	89.0440	27.6121
T. stenura	97.0000	32.9900
T. stenura	97.0100	32.9900
T. stenura	97.5000	29.8700
T. stenura	98.6600	27.7400
T. stoliczkae	107.0460	35.1795
T. stoliczkae	105.1801	34.7598
T. stoliczkae	102.9274	37.3761
T. stoliczkae	103.0074	34.6742
T. stoliczkae	102.8514	36.3766

Species	Longitude	Latitude
T. stoliczkae	106.6139	35.3100
T. stoliczkae	106.5076	35.6484
T. stoliczkae	103.0373	35.4713
T. stoliczkae	102.2004	34.1814
T. stoliczkae	103.1844	34.6185
T. stoliczkae	104.0678	34.4027
T. stoliczkae	104.0826	34.4940
T. stoliczkae	104.1938	34.7877
T. stoliczkae	105.5856	34.2207
T. stoliczkae	105.5055	34.3672
T. stoliczkae	104.4219	33.9608
T. stoliczkae	104.2132	35.0148
T. stoliczkae	102.1320	34.8521
T. stoliczkae	102.8789	35.3639
T. stoliczkae	99.3404	37.1755
T. stoliczkae	100.1100	37.3639
T. stoliczkae	103.4173	36.3116
T. stoliczkae	102.0272	38.3315
T. stoliczkae	104.0138	35.8643
T. stoliczkae	107.4762	35.6132
T. stoliczkae	104.3516	33.7833
T. stoliczkae	104.0393	33.8289
T. stoliczkae	105.9582	35.3236
T. stoliczkae	106.3939	33.8235
T. stoliczkae	106.2049	33.7126
T. stoliczkae	97.8010	33.4040
T. stoliczkae	97.1950	33.7130
T. stoliczkae	101.0140	36.8900
T. stoliczkae	100.6080	37.9250
T. stoliczkae	102.6080	37.9250
T. stoliczkae	82.5110	43.3330
T. stoliczkae	82.4740	43.7550
T. stoliczkae	83.3400	43.7550
T. stoliczkae	80.3130	42.7560
T. stoliczkae	81.4540	30.7490
T. stoliczkae	78.6500	35.7710
T. stoliczkae	76.2350	37.4370
T. stoliczkae	79.0600	34.0990

Table S4. The optimal nucleotide substitution model is supported by nine genes, with AICc, and BIC values calculated accordingly. −lnL, AICc, and BIC values were obtained from PartitionFinder v2.1.1 and that AICc was used for model selection.

**Genes**	**Model**	**- lnL**	**AICc**	**BIC**
COI	TVM+F+R6	58652.061	119305.784	119479.222
cytb	TVM+F+R6	50882.810	103992.375	104314.617
COII	TPM2u+F+I+G4	23126.832	50383.722	47808.432
COIII	TPM2u+F+R5	27273.204	57690.566	55761.768
ATP6	GTR+F+I+I+R7	87841.317	65519.739	64634.176
ATP8	JC	6974.024	541468.261	11876.082
ND1	GTR+F+I+R5	47290.041	97161.952	97347.436
ND2	GTR+F+R6	56130.332	114642.267	114583.719
ND3	TPM2u+F+I+G4	28068.528	562919.519	33215.202
ND4	GTR+F+R7	66610.195	135387.092	135450.956
ND4L	TPM2u+F+I+G4	11582.044	553708.331	24029.969
ND5	GTR+F+I+R7	88440.640	178939.125	179360.985
ND6	JC	32548.114	581286.196	51241.569
12S rRNA	TIM2+F+R5	18010.771	38214.362	39750.854
16S rRNA	GTR+F+I+I+R5	43823.584	89212.847	91831.183

Table S5. Pairwise genetic differentiation (F_{ST}) among 12 *Triplophysa* species based on *COI* (lower triangle) and *cytb* (upper triangle) sequences.

	1	2	3	4	5	6	7	8	9	10	11	12
1 *T. hsutschouensis*		0.9678	—	0.9494	0.7591	0.9629	0.9673	0.8447	0.9824	0.9486	0.9675	0.8478
2 *T. dalaica*	0.8639		—	0.9090	0.9271	0.9535	0.9649	0.5180	0.9265	0.8757	0.8875	0.6390
3 *T. obscura*	0.9333	0.7521		—	—	—	—	—	—	—	—	—
4 *T. stenura*	0.9211	0.8262	0.8906		0.9375	0.9434	0.9479	0.7935	0.9266	0.8265	0.9211	0.7092
5 *T. robusta*	0.5814	0.8733	0.8867	0.8924		0.8775	0.8863	0.8689	0.9434	0.9243	0.9428	0.8643
6 *T. siluroides*	0.9273	0.8726	0.9478	0.9238	0.7918		0.5912	0.7198	0.9881	0.9422	0.9485	0.7760
7 *T. minxianensis*	0.9307	0.8635	0.9429	0.9215	0.7976	0.4710		0.7635	0.9918	0.9513	0.9595	0.8113
8 *T. leptosoma*	0.7787	0.4962	0.6477	0.7630	0.8504	0.7998	0.7761		0.4422	0.6584	0.6011	0.3702
9 *T. orientalis*	0.7355	0.4802	0.5563	0.7237	0.8200	0.7703	0.7406	0.2012		0.9009	0.8969	0.6685
10 *T. brevicauda*	0.7018	0.5499	0.6149	0.4759	0.7949	0.7018	0.6851	0.4312	0.3938		0.8969	0.5924
11 *T. bleekeri*	0.7663	0.6959	0.7377	0.7973	0.8347	0.8207	0.8013	0.5442	0.4470	0.5416		0.7024
12 *T. stoliczkae*	0.7098	0.4385	0.3813	0.6103	0.7900	0.7158	0.7014	0.2303	0.2666	0.3822	0.5744	

Table S6. Mean pairwise genetic distances (K2P) among 12 *Triplophysa* species based on *COI* gene. Intraspecific distances (mean±SE) are shown on the diagonal (bold), and interspecific distances (mean±SE) are shown below the diagonal. SE means the Standard Error.

	T. hsutschouensis	*T. leptosoma*	*T. obscura*	*T. orientalis*	*T. siluroides*	*T. stoliczkae*	*T. bleekeri*	*T. brevicauda*	*T. dalaica*	*T. stenura*	*T. robusta*	*T. minxianensis*
T. hsutschouensis	**0.0058±0.0018**											
T. leptosoma	0.1451±0.0132	**0.0581±0.0063**										
T. obscura	0.1231±0.0148	0.1006±0.0110	**0.0139±0.0031**									
T. orientalis	0.1339±0.0123	0.0769±0.0077	0.0879±0.0103	**0.0614±0.0071**								
T. siluroides	0.0589±0.0114	0.1324±0.0129	0.1251±0.0156	0.1282±0.0130	**0.0046±0.0016**							
T. stoliczkae	0.1346±0.0132	0.0744±0.0075	0.0643±0.0073	0.0817±0.0085	0.1283±0.0134	**0.0575±0.0064**						
T. bleekeri	0.1351±0.0132	0.1067±0.0117	0.1212±0.0144	0.0923±0.0107	0.1433±0.0154	0.1157±0.0127	**0.0436±0.0046**					
T. brevicauda	0.1329±0.0139	0.1014±0.0110	0.1016±0.0122	0.0947±0.0107	0.1262±0.0144	0.0922±0.0102	0.1070±0.0126	**0.0539±0.0046**				
T. dalaica	0.1409±0.0151	0.0809±0.0084	0.0911±0.0111	0.0832±0.0083	0.1299±0.0155	0.0797±0.0086	0.1144±0.0135	0.0998±0.0117	**0.0307±0.0039**			
T. stenura	0.1221±0.0147	0.0978±0.0109	0.0951±0.0136	0.0907±0.0108	0.1212±0.0152	0.0836±0.0099	0.1072±0.0139	0.0628±0.0074	0.0964±0.0124	**0.0135±0.0028**		
T. robusta	0.0351±0.0058	0.1365±0.0131	0.1237±0.0146	0.1256±0.0121	0.0657±0.0098	0.1296±0.0132	0.1303±0.0142	0.1336±0.0149	0.1312±0.0144	0.1238±0.0154	**0.0181±0.0034**	
T. minxianensis	0.0619±0.0116	0.1344±0.0131	0.1239±0.0157	0.1272±0.0128	0.0076±0.0021	0.1282±0.0136	0.1426±0.0154	0.1202±0.0137	0.1332±0.0157	0.1147±0.0150	0.0704±0.0106	**0.0040±0.0008**
